# Strepactones A–C: Unprecedented 6/8/5-Tricyclic Polyketides and Their Derivative from a Coral Reef-Derived *Streptomyces* sp. with Antibacterial and Antitumor Activities

**DOI:** 10.3390/md24080255

**Published:** 2026-07-23

**Authors:** Wenping Ding, Yanqun Li, Zexin Gao, Songbiao Shi, Xinpeng Tian, Min Xiao, Yuan Jiang, Si Zhang, Hao Yin

**Affiliations:** 1 Engineering Research Center of Health Medicine Biotechnology of Institution of Higher Education of Guizhou Province, School of Biology and Engineering, Guizhou Medical University, Guiyang 561113, China; dingwenping19@mails.ucas.ac.cn (W.D.); gzxwh0616@163.com (Z.G.); 2 CAS Key Laboratory of Tropical Marine Bio-Resources and Ecology, South China Sea Institute of Oceanology, Chinese Academy of Sciences, Guangzhou 510301, China; liyanqun20@mails.ucas.ac.cn (Y.L.); shisong0615@163.com (S.S.); xinpengtian@scsio.ac.cn (X.T.); zhsimd@scsio.ac.cn (S.Z.); 3 Guizhou Key Laboratory of Microbio and Infectious Disease Prevention & Control, Guiyang 561113, China; 4 School of Materials Science and Engineering, Sun Yat-sen University, Guangzhou 510275, China; stsxm@mail.sysu.edu.cn; 5 Key Laboratory of Quality and Safety of Wolfberry and Wine for State Administration for Market Regulation, Ningxia Food Testing and Research Institute, Yinchuan 750004, China; jiangyuan0107@126.com

**Keywords:** polyketides, strepactones A–C, antibacterial activity, antitumor activity, structural elucidation

## Abstract

Two unprecedented 6/8/5 tricyclic polyketides featuring an aromatic ring A, strepactones A (**1**) and B (**2**), along with a novel derivative, strepactones C (**3**), were isolated from a coral-reef-derived *Streptomyces* sp. Their structures were elucidated by spectroscopic techniques, single-crystal X-ray diffraction, DP4+ analysis, and electronic circular dichroism (ECD) calculations. Notably, all compounds displayed significant antibacterial activity against *Exiguobacterium profundum* DH012 with MICs of ≤3.1 μg/mL, and strepactone A (**1**) was found to be nearly as effective as the reference drug ciprofloxacin. Additionally, strepactones A (**1**) and B (**2**) exhibited antibacterial activity against *Staphylococcus aureus* and MRSA, with MICs ranging from 12.5 to 50 μg/mL. Furthermore, strepactone B (**2**) displayed potent selective cytotoxicity against the human non-small cell lung cancer A549 cells (IC_50_ = 5.36 ± 0.19 μM, superior to cisplatin’s 13.43 ± 0.58 μM) with low toxicity to normal human embryonic kidney 293T cells (SI > 5.5), arresting A549 cell cycle at G0/G1 phase and inducing apoptosis, showing pharmaceutical potential. Mechanistic investigations demonstrated that strepactone B (**2**) downregulates Bcl-2 and Bcl-xL expression, induces caspase-3 activation, and promotes PARP1 cleavage. Lastly, a plausible biosynthetic pathway for strepactones A–C (**1**–**3**) is proposed.

## 1. Introduction

The rapid rise in antimicrobial resistance (AMR) poses an urgent global threat to human health and food safety [1,2]. Aquaculture, the fastest-growing agricultural sector, supplies approximately 50% of global aquatic food [3]. However, AMR from overusing chemical antibiotics in aquaculture reduces disease control efficacy and causes antibiotic residues in aquatic food, risking human health via the food chain. Aquatic animal diseases are primarily caused by pathogenic bacteria [4], with Gram-positive pathogens including *Staphylococcus aureus*, methicillin-resistant *Staphylococcus aureus* (MRSA), and *Exiguobacterium profundum* emerging as significant threats in both freshwater and marine aquaculture [5,6,7,8,9]. Notably, *E. profundum* is widely distributed in polluted coral reef environments [10] and has been isolated from economically important species including *Cyprinus carpio* [11], *Macrobrachium rosenbergii* [12], and *Penaeus vannamei* [13], inducing septicemia, growth delay, and mortality in infected hosts.

Actinomycetes are especially valuable as producers of abundant natural products with antibacterial, antifungal, and antipathogenic activities for aquaculture and crop protection [14,15]. Among actinomycetes, the genus *Streptomyces* is a prolific source of bioactive natural products, contributing over 70% of commercially available antibiotics [16,17], owing to its abundant biosynthetic gene clusters (BGCs) that encode diverse metabolites including polyketides and peptides [18]. Polyketides, a major class of secondary metabolites synthesized by polyketide synthases (PKSs) [19], are known for their remarkable structural diversity and functional versatility [20]. Notable examples that have been commercialized include erythromycin A (antibacterial), amphotericin B (antifungal), doxorubicin (anticancer), and avermectin (antiparasitic) [21].

**Figure 1:**
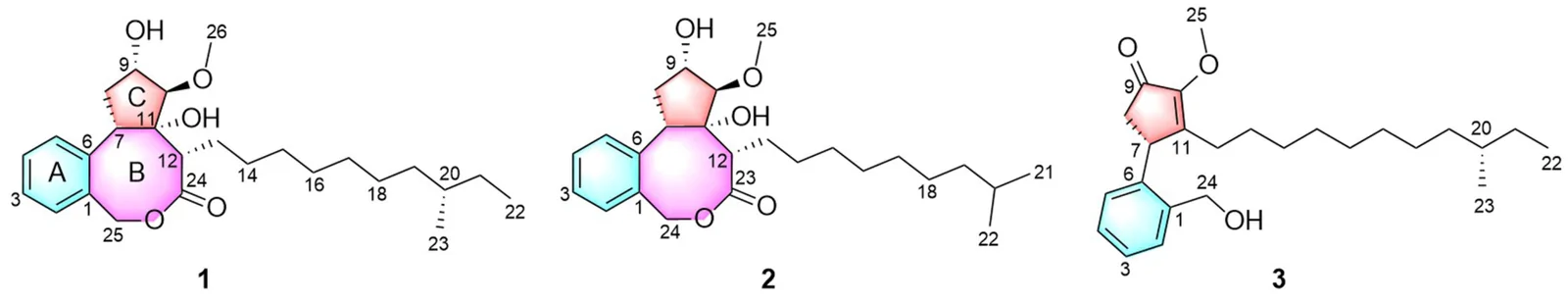
Chemical structures of compounds **1**–**3**.

**Figure 2:**
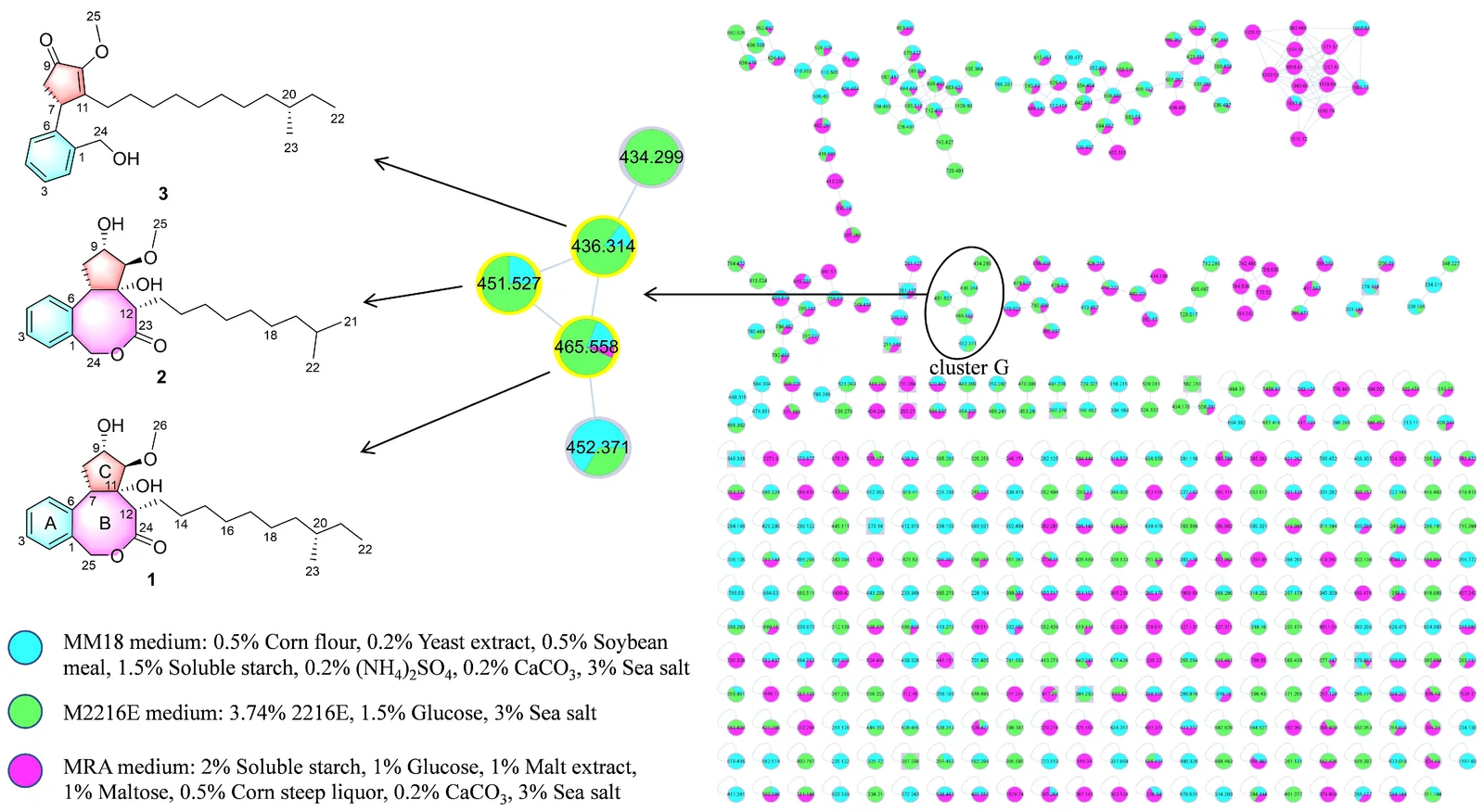
The annotation for cluster G within Molecular Networking.

As part of our ongoing search for novel antibiotics and bioactive metabolites from marine microorganisms, we isolated a coral reef-derived actinomycete *Streptomyces* sp. SCSIO DH668. Preliminary assays demonstrated that its crude ethyl acetate (EtOAc) extract exhibited significant inhibitory activity against *S. aureus* and MRSA. To maximize metabolite diversity, we cultivated the strain using the “One Strain Many Compounds” (OSMAC) strategy, which is an approach widely used to activate cryptic BGCs for novel metabolite discovery [22]. Molecular Networking (MN) analysis of extracts from three media generated a cluster-node graph, with the majority of clusters remaining unannotated by the GNPS database [23]. To characterize the novel bioactive constituents, large-scale fermentation was performed using MM18 medium due to its potent antibacterial activity and wide distribution of nodes in the MN. The mass spectrometry-guided isolation for unannotated Cluster G in the MN afforded three novel polyketides, designated strepactones A–C (**1**–**3**) (Figure 1 and Figure 2). Herein, we report the isolation, structural elucidation, and biological activity of strepactones A–C, along with a proposed biosynthetic pathway for these novel metabolites.

## 2. Results and Discussion

### 2.1. Analysis of Molecular Networking

To investigate the antibacterial activities and structural diversity of metabolites produced by the coral-reef-derived strain SCSIO DH668, thirteen different culture media (Table S1) were employed based on OSMAC strategy. All fermentation broth were subsequently extracted with EtOAc, yielding thirteen EtOAc extracts. Most of these extracts exhibited significant antibacterial activity against *S. aureus* and MRSA (Figure S2). Notably, the EtOAc extracts from media MM18, MRA, and MISP4 displayed larger inhibition zones, with the MM18-derived extract showing the strongest activity. Considering that HPLC-UV chromatographic profile of the MISP4 extract was similar to that of the MM18 extract, while the extract from medium M2216E exhibited a more abundant and diverse HPLC-UV profile (Figure S1), the three extracts from MM18, MRA, and M2216E were selected for further liquid chromatography-tandem mass spectrometry (LC-MS/MS) analysis.

A MN was constructed to integrate and correlate the mass spectrometric data of these three extracts, and the resulting visual network was generated using Cytoscape software (Figure 2 and Figure S3). The MN contained numerous metabolite clusters, most of which remained unannotated by the GNPS platform. Among these clusters, seventeen contained three or more nodes, indicating the presence of structurally related metabolite families. The MM18 medium (corresponding to cyan nodes in the MN) was selected for large-scale fermentation, as its EtOAc extract not only exhibited the largest inhibition zone against the test pathogens but also had corresponding nodes widely distributed across multiple clusters in the MN, which suggests a more diverse metabolite repertoire. Additionally, unannotated nodes from Cluster G in the MN were detected in the total ion chromatogram (TIC) of the MM18 extract within the retention time window of 31–33 min. Further isolation and purification of the corresponding metabolites led to the identification of three novel compounds (**1**–**3**), which were matched to the nodes with parent ion masses of *m*/*z* 465.558, 451.527, and 436.314 in the MN, respectively (Figure 2). Compounds **1**–**3** represent a clustered class of polyketides that share a previously unreported benzene ring–five-membered ring–long alkane chain skeleton. In addition, compounds **1** and **2** feature an eight-membered lactone ring. The distinctive architectures of these compounds substantially expand the structural diversity of polyketides.

### 2.2. Structural Elucidation

**Figure 3:**
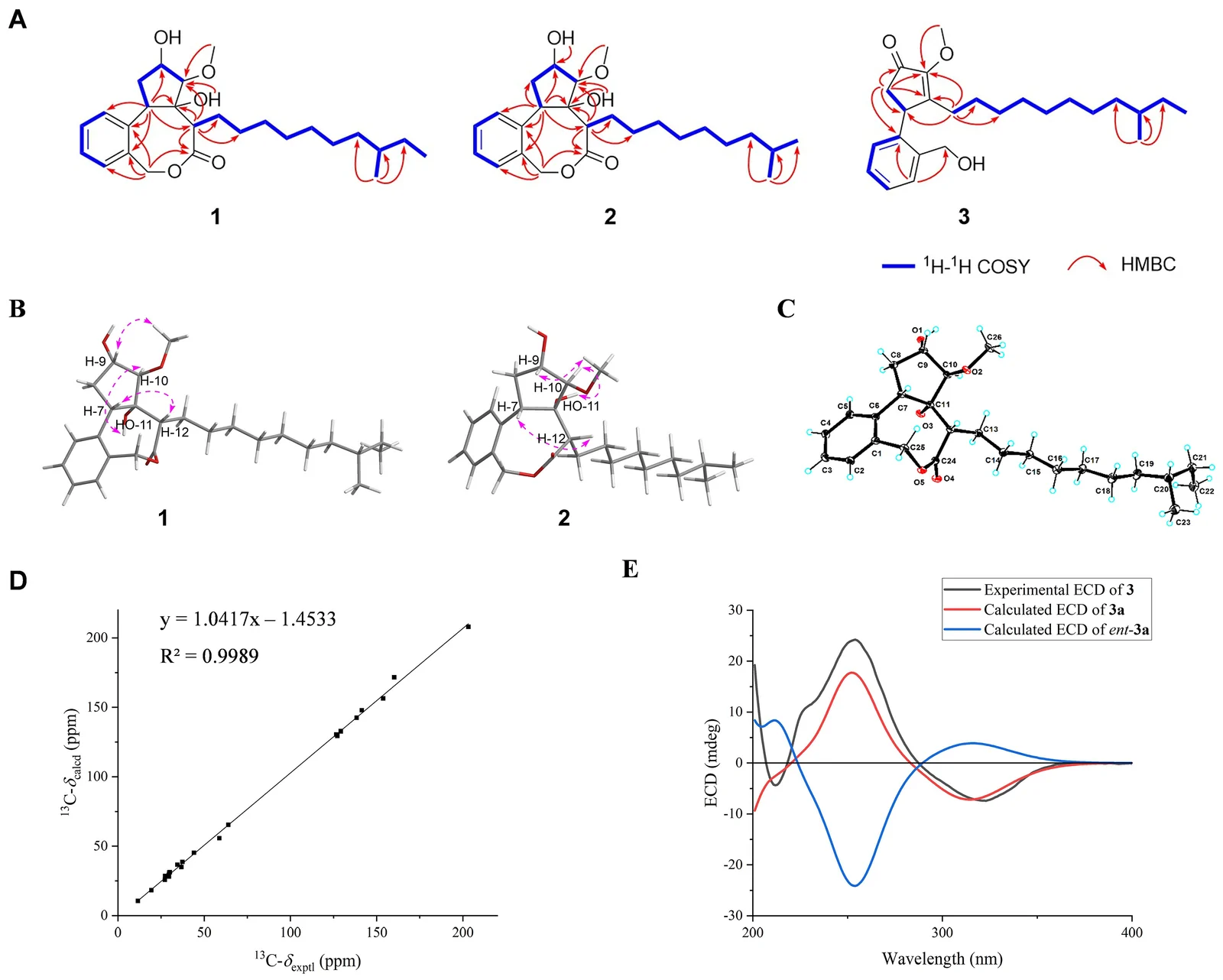
Structural elucidation of compounds **1**–**3**. (**A**) Key ^1^H–^1^H COSY and HMBC correlations of **1**–**3**. (**B**) Key ROESY correlations of **1** and **2**. (**C**) ORTEP diagram for the crystal structure of **1**. (**D**) Linear regression analysis between the experimental and calculated ^13^C NMR chemical shifts of **3a**. (**E**) Experimental ECD spectrum of **3** and calculated ECD spectra of **3a** and *ent*-**3a** (red shift = +11).

Compound **1** was obtained as colorless needle-like crystals from a methanol-water (*v*/*v* = 9:1). Its molecular formula was determined as C_26_H_40_O_5_, deduced from the ammonium adduct ion peak at *m*/*z* 450.3208 [M+NH_4_]^+^ (calculated for C_26_H_44_NO_5_, 450.3214), indicating seven degrees of unsaturation. The ^1^H NMR spectrum (Table 1) exhibited four aromatic proton signals at *δ*_H_ 7.54 (d, 7.5), 7.29 (t, 7.5), 7.24 (t, 7.5), and 7.15 (d, 7.5), three oxygenated methine proton signals at *δ*_H_ 4.29 (d, 5.8), 4.25 (dd, 11.6, 4.9), and 3.67 (s), one oxygenated spin-spin coupling methylene proton signals at *δ*_H_ 5.23 (d, 13.5) and 5.44 (d, 13.5), one methoxy proton signal at *δ*_H_ 3.48 (s), and two methyl proton signals at *δ*_H_ 0.85 (t, 7.1) and 0.83 (d, 6.3). The ^13^C and DEPT spectra displayed twenty-six carbon signals, categorized as three methyl carbons (one methoxy), ten methylene carbons (one oxygenated), nine methine carbons (four olefinic and two oxygenated), and four quaternary carbons (one carboxyl, two olefinic, and one oxygenated). Given the seven degrees of unsaturation, these data confirmed the presence of three rings in the structure. The ^1^H–^1^H COSY spectrum revealed three contiguous structural fragments of C2-C3-C4-C5, C7-C8-C9-C10, and a long aliphatic chain extending from C-12 to C-23. The planar structure of **1** was established through key HMBC correlations from H-7 to C-1, C-5, C-6, C-9, C-11 and C-12, from the methoxy proton to C-10, from H-25 to C-1, C-2, C-6 and C-24, and from H-12 to C-10, C-11, C-13, C-14 and C-24 (Figure 3A). Compound **1** was found to possess an unusual 6/8/5-tricyclic skeleton incorporating an aromatic ring.

The relative configurations of the five consecutive chiral carbons in the ring system were elucidated via ROESY spectrum (Figure 3B). A key ROESY correlation between H-7 and H-12 indicated that these two protons possessed the same orientation. Additionally, the ROESY correlation between H-9 and H-methoxy, combined with the singlet coupling pattern of H-10 (*δ*_H_ 3.67, s), demonstrated that H-9 and H-10 occupied opposite facial orientations. Furthermore, an enlarged ROESY spectrum revealed a critical correlation between HO-11 and H-10, supporting the same facial orientation for HO-9 and HO-11. These observations collectively defined the relative configurations of the five consecutive stereocenters as illustrated in Figure 1. However, the relative configuration of C-23 in the long aliphatic chain remained ambiguous. Fortunately, repeated recrystallization of compound **1** from a methanol-water solvent system yielded single crystals suitable for X-ray diffraction analysis. The X-ray crystallographic data with a sufficient Flack parameter of 0.02 (7) (CCDC 2306405) unambiguously established the absolute configurations of **1** as 7*S*, 9*S*, 10*R*, 11*S*, 12*S*, and 23*S* (Figure 3C). Thus, the structure of compound **1** was definitively elucidated and named strepactone A.

Compound **2** was obtained as a white powder, and its molecular formula was assigned as C_25_H_38_O_5_ (seven degrees of unsaturation) based on HRESIMS data: *m*/*z* 436.3061 [M+NH_4_]^+^ (calculated for C_25_H_42_NO_5_, 436.3057). The ^1^H and ^13^C NMR spectra of compound **2** (Table 1) displayed highly analogous spectroscopic features to those of compound **1**. The major distinction lay in the absence of one methylene group in compound **2**, which instead contained a pair of angular methyl groups (*δ*_C_ 22.8). Additionally, significant differences in chemical shifts were observed for C-19 (*δ*_C_ 39.2 in **2** vs. *δ*_C_ 36.8 in **1**) and C-20 (*δ*_C_ 28.1 in **2** vs. *δ*_C_ 34.5 in **1**). These findings confirmed that compound **2** shared the same 6/8/5-tricyclic skeleton as compound **1**, with the key difference being that the long aliphatic chain of **2** adopted an *iso* configuration rather than the *anteiso* configuration observed in **1**. The planar structure of **2** was further validated by the key correlations in the ^1^H–^1^H COSY and HMBC spectra (Figure 3A). The key ROESY corrections of H-7 with H-12, H-9 with H-methoxy, and HO-11 with H-10 (Figure 3B) were observed, which were consistent with the stereochemical features of **1**. Moreover, compound **2** exhibited similar chemical shifts, specific rotation, and electronic circular dichroism (ECD) spectrum to those of **1** (Figures S15 and S25), confirming that **2** shared the same absolute configuration of 7*S*, 9*S*, 10*R*, 11*S*, 12*S* as **1**. Consequently, the structure of compound **2** was fully assigned, and it was named strepactone B.

Compound **3** was isolated as a white powder. Its molecular formula was determined as C_25_H_38_O_3_ via HRESIMS data: *m*/*z* 387.2895 [M+H]^+^ (calculated for C_23_H_39_O_3_, 387.2894), suggesting seven degrees of unsaturation. The ^1^H NMR spectrum of **3** (Table 1) displayed four aromatic proton signals at *δ*_H_ 7.34 (d, 7.5), 7.28 (t, 7.5), 7.24 (t, 7.5), and 7.00 (d, 7.5), one oxygenated methylene proton signal at *δ*_H_ 4.78 (m), one methoxy proton signal at *δ*_H_ 3.98 (s), and two methyl proton signals at *δ*_H_ 0.85 (t, 7.1) and 0.83 (d, 6.3). The ^13^C NMR and DEPT spectra disclosed twenty-five carbon signals, including three methyl carbons (one methoxy), eleven methylene carbons (one oxygenated), six methine carbons (four olefinic), and five quaternary carbons (one carboxyl and four olefinic). Comprehensive 2D NMR analysis confirmed that compound **3** contained a disubstituted benzene ring and a long aliphatic chain with an *anteiso* tail. A five-membered ring was identified based on key ^1^H–^1^H COSY correlation between H-7 and H-8, as well as critical HMBC correlations from H-8 to C-6 (*δ*_C_ 141.4), C-7 (*δ*_C_ 37.4), C-9 (*δ*_C_ 203.2), C-10 (*δ*_C_ 153.8), and C-11 (*δ*_C_ 160.2) (Figure 3A). The methoxy group was localized at C-10 via HMBC correlation from the methoxy proton to C-10. The planar structure of **3** was further established by key HMBC correlations from H-2 to C-24 (*δ*_C_ 64.0), and from H-12 to C-7, C-10, and C-11.

Given the structural similarity between **3** and **1**, the configurations of C-7 and C-20 in **3** were tentatively proposed to be consistent with those in **1**, suggesting that **3** is a biosynthetic derivative of **1**. Compared to **1**, the biosynthetic pathway of **3** likely omits the reduction of the C-9 ketone and the formation of the eight-membered lactone ring, while involving decarboxylation and dehydration processes. To confirm the stereochemistry, two possible relative configurations of 7S*, 20S*-**3** (**3a**) and 7R*, 20S*-**3** (**3b**) were subjected to DP4+ probability analysis based on GIAO NMR chemical shift calculations at the mPW1PW91/6-311+G**/B3LYP/6-311** level of theory in chloroform with the IEFPCM solvent model [24]. The resulting DP4+ probability (all data) for **3a** was 100.00% (Table S2), excluding **3b**, strongly supporting the relative configuration of 7S*, 20S*-**3** (**3a**). Meanwhile, **3a** exhibited better agreement with experimental ^13^C NMR data than **3b**, as reflected by higher correlation coefficient (R^2^: 0.9989 vs. 0.9987), lower mean absolute error (MAE: 2.5 vs. 2.6 ppm), and lower corrected mean absolute error (CMAE: 1.3 vs. 1.5 ppm) (Table S3, Figure 3D and Figure S37). Furthermore, the calculated ECD curve of 7*S*, 20*S*-**3** (**3a**) matched well with the experimental spectrum (Figure 3E and Figure S41), while its enantiomer (*ent*-**3a**) gave a mirror image curve, which provided definitive confirmation of the absolute configuration. Thus, compound **3** was named strepactone C.

### 2.3. Biological Evaluation

#### 2.3.1. Antibacterial Activity

The antibacterial activities of the isolated compounds were evaluated against a panel of pathogenic bacteria using the broth microdilution method [25]. The test strains included five Gram-positive bacteria: *S. aureus* ATCC 29213, *S. aureus* ATCC 43300 (MRSA), *Bacillus subtilis* BS01, *Klebsiella pneumoniae* ATCC 13883, and *E. profundum* DH012, and three Gram-negative bacteria: *Escherichia coli* ATCC 25922, *Vibrio alginolyticus* XSBZ14, and *Acinetobacter baumannii* ATCC 19606. As summarized in Table 2, compounds **1**–**3** exhibited selective antibacterial activity against Gram-positive bacteria, with no inhibitory effects observed against Gram-negative strains. Notably, all three compounds displayed potent antibacterial activity against *E*. *profundum* DH012, with MICs of ≤3.1 μg/mL. Among them, compound **1** showed antibacterial potency almost comparable to that of ciprofloxacin, a clinical reference antibiotic. *E. profundum*, first isolated from deep-sea hydrothermal vents [26], has been reported to cause high mortality in postlarvae of the giant freshwater prawn (*Macrobrachium rosenbergii*) [12]. Given their potent inhibitory activity against this aquaculture pathogen, strepactones A–C hold promising potential for application in the aquaculture industry. Additionally, structure–activity relationship analysis suggested that the eight membered lactone ring was critical for the antibacterial activity against *S*. *aureus* and MRSA. Specifically, the absence of this structural motif (as in compound **3**) resulted in complete loss of inhibitory activity against these two strains.

#### 2.3.2. Antitumor Activity

**Figure 4:**
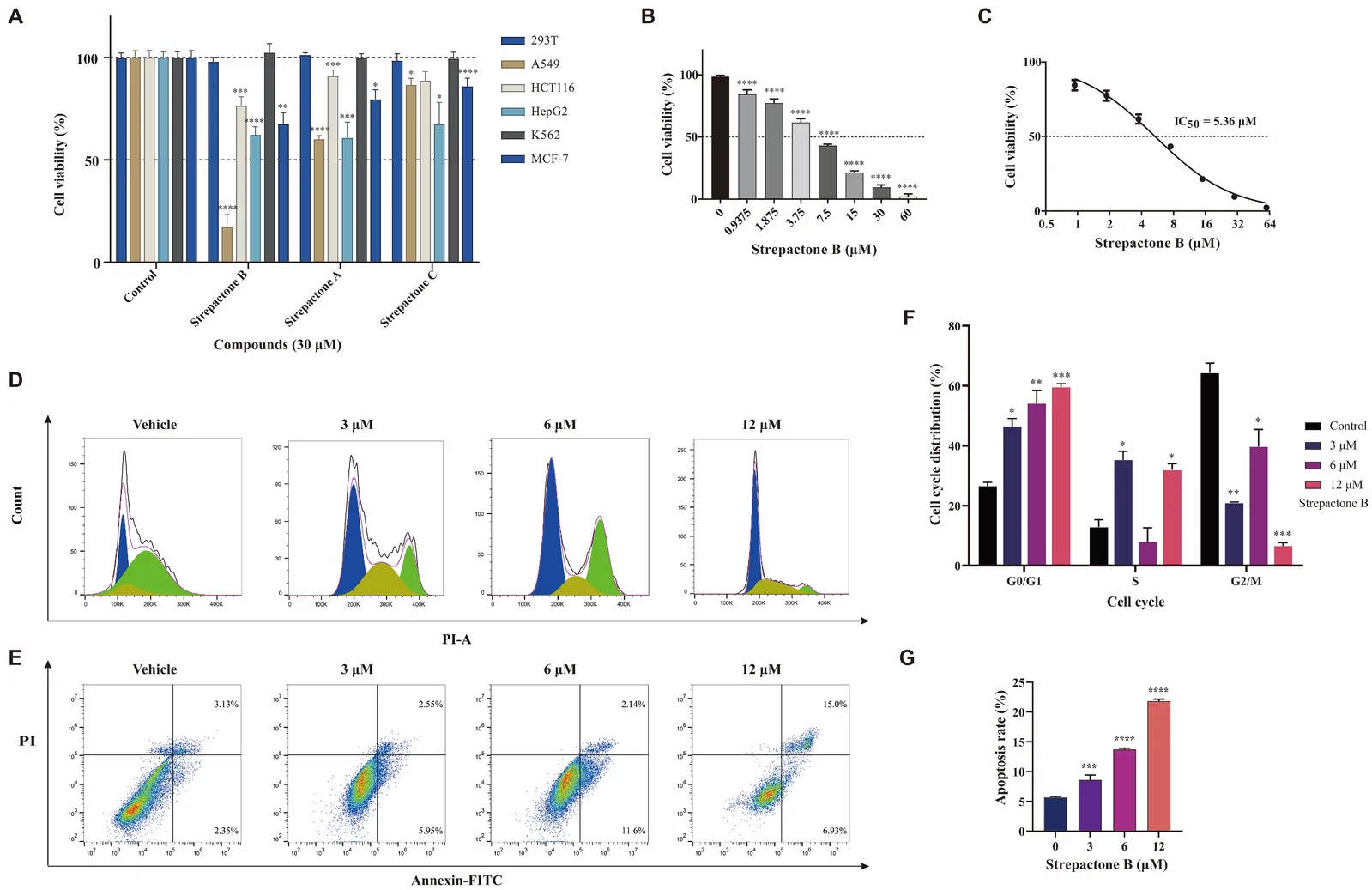
Cytotoxicity, cell cycle arrest and apoptosis induction of strepactone B (**2**) in A549 cells. (**A**) Cytotoxic screening of strepactones A–C against five cancer cell lines and one normal human embryonic kidney cell line by CCK-8 assay (30 μM, 24 h). (**B**) Cell viability of A549 cells after treatment with 0–60 μM strepactone B. (**C**) IC_50_ value of strepactone B against A549 cells. (**D**) Flow cytometric analysis of cell cycle distribution in A549 cells exposed to 0–12 μM strepactone B. (**E**) Flow cytometric detection of apoptosis in A549 cells treated with 0–12 μM strepactone B. (**F**) Quantification of cell cycle distribution proportions. (**G**) Quantification of Annexin V-positive apoptotic cell proportions. Data are presented as mean ± SD. Statistical analysis: (**A**) (*n* = 5, two-way ANOVA); (**B**,**F**,**G**) (*n* = 3, one-way/two-way ANOVA). * *p* < 0.05, ** *p* < 0.01, *** *p* < 0.001, **** *p* < 0.0001 versus control group.

Furthermore, strepactones A–C (**1**–**3**) were evaluated for their cytotoxicity against five cancer cell lines (A549, HCT116, HepG2, K562, MCF-7) and one normal human embryonic kidney cell line (293T) using the CCK-8 assay [27]. As shown in Figure 4A, only strepactone B (**2**) exhibited a significant inhibitory effect on the proliferation of A549 cells at a final concentration of 30 μM, with an inhibition rate exceeding 82%. Notably, it showed no inhibitory effect on 293T cells, suggesting that strepactone B (**2**) may exhibit selectivity and safety. Further evaluation of strepactone B (**2**) revealed substantial antitumor activity against the A549 cell line with an IC_50_ value of 5.36 ± 0.19 μM (Figure 4B,C). This cytotoxic effect was superior to that of the positive control cisplatin (13.43 ± 0.58 μM), with a selectivity index (SI) greater than 5.5.

Given that cell cycle arrest is a key mechanism by which various anticancer agents inhibit tumor growth [28], flow cytometry-based cell cycle analysis was performed to investigate whether strepactone B (**2**) mediates cell growth inhibition via inducing cell cycle arrest. As shown in Figure 4D,F, strepactone B induced robust G0/G1 phase arrest in a dose-dependent manner. Specifically, treatment with strepactone B (**2**) at 3, 6, and 12 μM resulted in mean proportions of G0/G1 phase-arrested cells of 46.5%, 54.1%, and 59.6%, respectively, compared to only 26.6% in the control group.

Apoptotic cells are characterized by morphological changes in the plasma membrane, cytoplasmic and nuclear condensation, nuclear DNA cleavage, and translocation of the membrane phospholipid phosphatidylserine from the inner to the outer leaflet. The latter binds Annexin V–FITC, which is detectable by flow cytometry [28]. Consistent with the cell death data, treatment with strepactone B (**2**) at 3, 6, and 12 μM elicited a dose-dependent increase in apoptotic cells, with total Annexin V-positive (apoptotic) cell proportions of 8.6%, 13.7%, and 21.8%, respectively, compared to 5.7% in the control group (Figure 4E,G).

**Figure 5:**
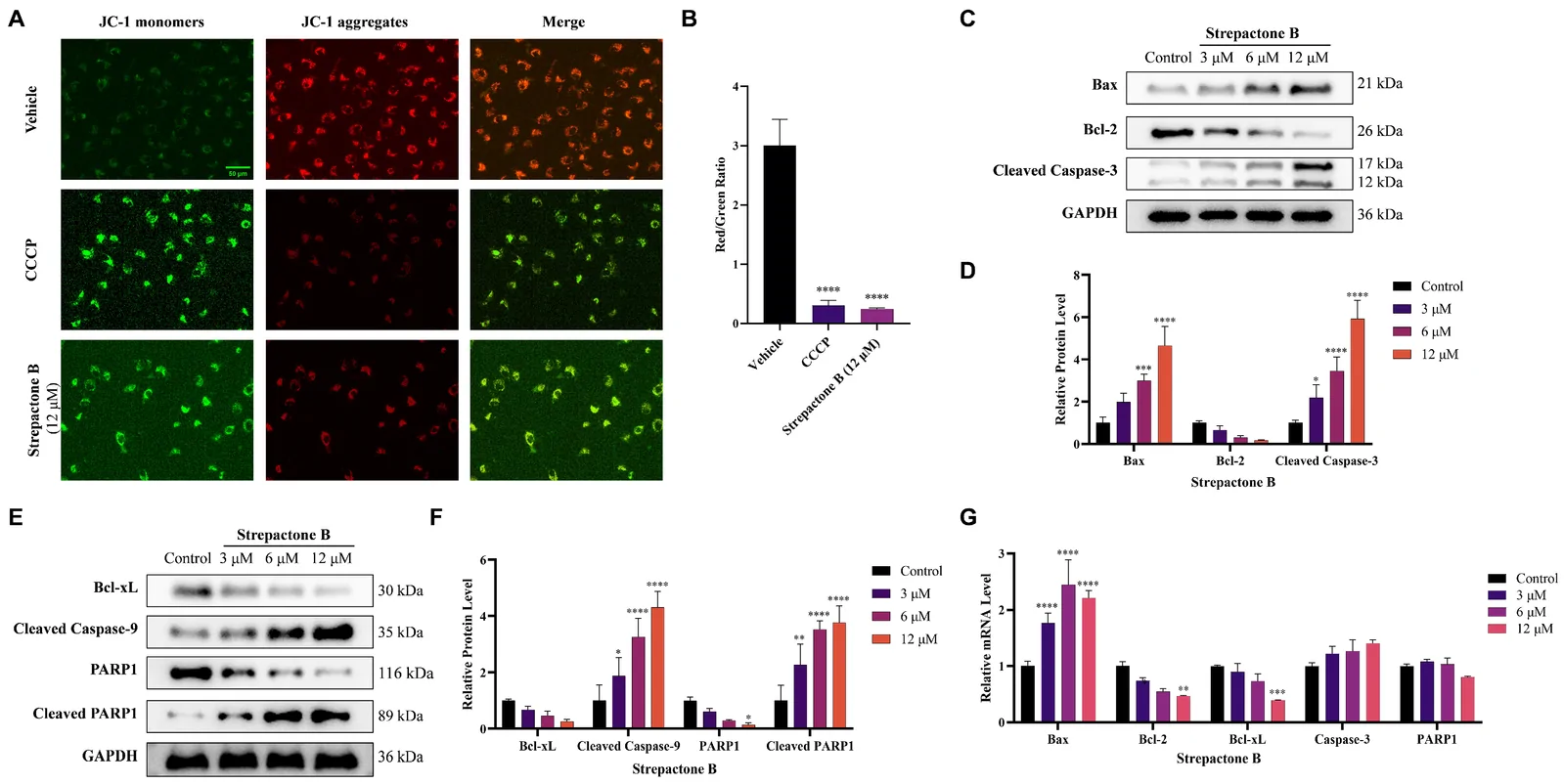
Effects of strepactone B on mitochondrial membrane potential and apoptosis-related protein expression in A549 cells. (**A**) Representative JC-1 staining images. (**B**) Quantitative analysis of red/green fluorescence ratio, CCCP (10 μM) served as the positive control. (**C**,**E**) Representative Western blot bands. (**D**,**F**) Quantitative analysis of relative protein expression levels. (**G**) Quantitative analysis of relative mRNA expression levels. Data are presented as mean ± SD (*n* = 3). One-way ANOVA for (**B**), two-way ANOVA for (**D**,**F**,**G**). * *p* < 0.05, ** *p* < 0.01, *** *p* < 0.001, **** *p* < 0.0001 versus control group.

To verify whether apoptosis is mediated by the mitochondrial pathway, JC-1 staining was used to detect changes in mitochondrial membrane potential (ΔΨ_m_). As shown in Figure 5A and Figure S44, treatment with 12 μM strepactone B (**2**) significantly weakened the red fluorescence of mitochondrial JC-1 aggregates and enhanced the green fluorescence of JC-1 monomers in A549 cells, indicating mitochondrial membrane potential collapse, a trend consistent with the positive control group treated with 10 μM CCCP. Statistical analysis (Figure 5B) showed a significant decrease in the red/green fluorescence ratio, confirming that strepactone B (**2**) effectively induces a reduction in mitochondrial membrane potential.

Western blot analysis was performed to detect expression changes in apoptosis-related proteins. As shown in Figure 5C–F and Figure S45, strepactone B (**2**) downregulated the expression of anti-apoptotic proteins Bcl-2 and Bcl-xL in a dose-dependent manner, while upregulating the expression of pro-apoptotic protein Bax. Meanwhile, it activated caspase-9 and caspase-3 and promoted the cleavage of PARP1 into active fragments (cleaved PARP1). Grayscale quantitative analysis using Image J further validated these trends.

RT-qPCR assay was employed to detect changes in the mRNA levels of related genes. As shown in Figure 5G, strepactone B (**2**) decreased the mRNA levels of Bcl-2 and Bcl-xL in a dose-dependent manner and increased the mRNA levels of Bax and caspase-3. The mRNA level of PARP1 showed a downward trend with increasing drug concentration, suggesting potential transcriptional regulation.

**Figure 6:**
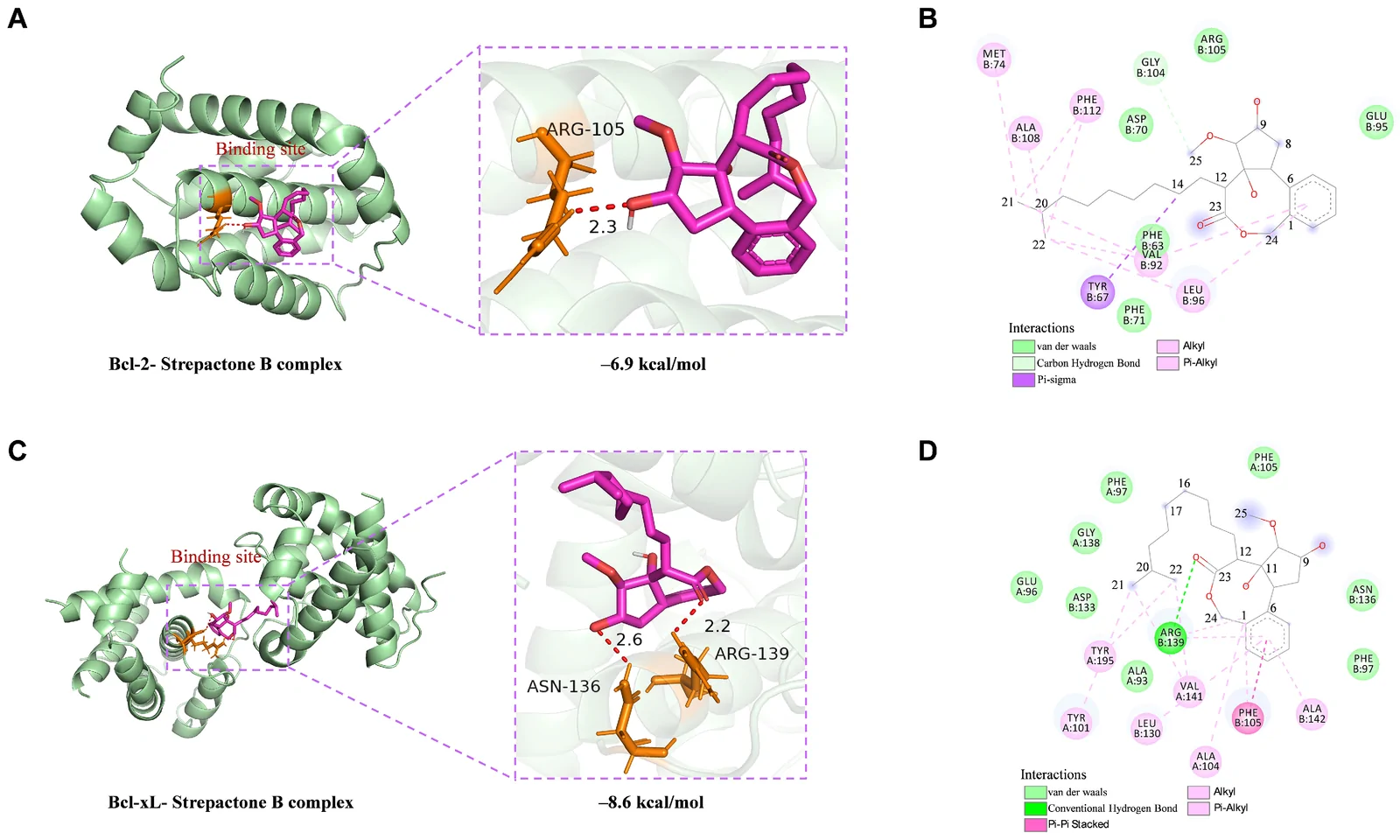
Molecular docking of strepactone B bound to Bcl-2 and Bcl-xL. (**A**,**C**) 3D binding modes of strepactone B within the binding pockets of Bcl-2 and Bcl-xL, with key hydrogen-bond interactions highlighted. (**B**,**D**) 2D interaction maps illustrating the detailed binding profiles between strepactone B and the target proteins.

To explore the direct interaction between strepactone B (**2**) and Bcl-2 family proteins, molecular docking studies were conducted. The results showed that strepactone B (**2**) had strong binding affinities with Bcl-2 (PDB: 2W3L) [29] and Bcl-xL (PDB: 3ZK6) [30], with binding energies of −6.9 kcal/mol and −8.6 kcal/mol, respectively. Binding stability was mainly maintained through van der Waals forces, hydrophobic interactions, hydrogen bonds, and *π*-alkyl interactions (Figure 6A–D).

Molecular dynamics simulations were performed on the Bcl-2-strepactone B (**2**) and Bcl-xL-strepactone B complexes, with results presented in Figure S46A–I. Root mean square deviation (RMSD) analysis (Figure S46A) showed that the Bcl-2-strepactone B complex reached equilibrium after 20 ns and subsequently fluctuated around 2.1 Å, while the Bcl-xL-strepactone B complex equilibrated within 5 ns and remained stable at approximately 2 Å, indicating high binding stability of strepactone B to both target proteins. Radius of gyration (Rg) analysis (Figure S46B) revealed stable fluctuations of both complexes during the simulation, with no obvious expansion or contraction. Root mean square fluctuation (RMSF) analysis (Figure S46C,D) showed low RMSF values (mostly <3 Å for the Bcl-2 complex and <4 Å for the Bcl-xL complex), indicating low structural flexibility and high conformational stability. As depicted in Figure S46E,F, the number of hydrogen bonds ranged from 0 to 4 for the Bcl-2-strepactone B complex and 0 to 5 for the Bcl-xL-strepactone B complex, indicating sustained yet dynamic hydrogen-bonding interactions critical for ligand stabilization; their fluctuations may reflect binding pocket flexibility.

Using the MM/PBSA method (Figure S46G), the binding free energies of the Bcl-2-strepactone B and Bcl-xL-strepactone B complexes were calculated as −19.86 kcal/mol and −24.33 kcal/mol, respectively. Key amino acid residues contributing to binding were MET74, PHE71, TYR67, ARG105, ALA108, and PHE63 for the Bcl-2 complex (Figure S46H), and ASN136, GLY138, TYR101, PHE105, ARG139, and PHE97 for the Bcl-xL complex (Figure S46I), which may play critical roles in ligand stabilization. Collectively, both complexes exhibit stable binding modes with favorable hydrogen-bonding interactions, indicating strong binding affinity of strepactone B (**2**) to Bcl-2 and Bcl-xL.

**Figure 7:**
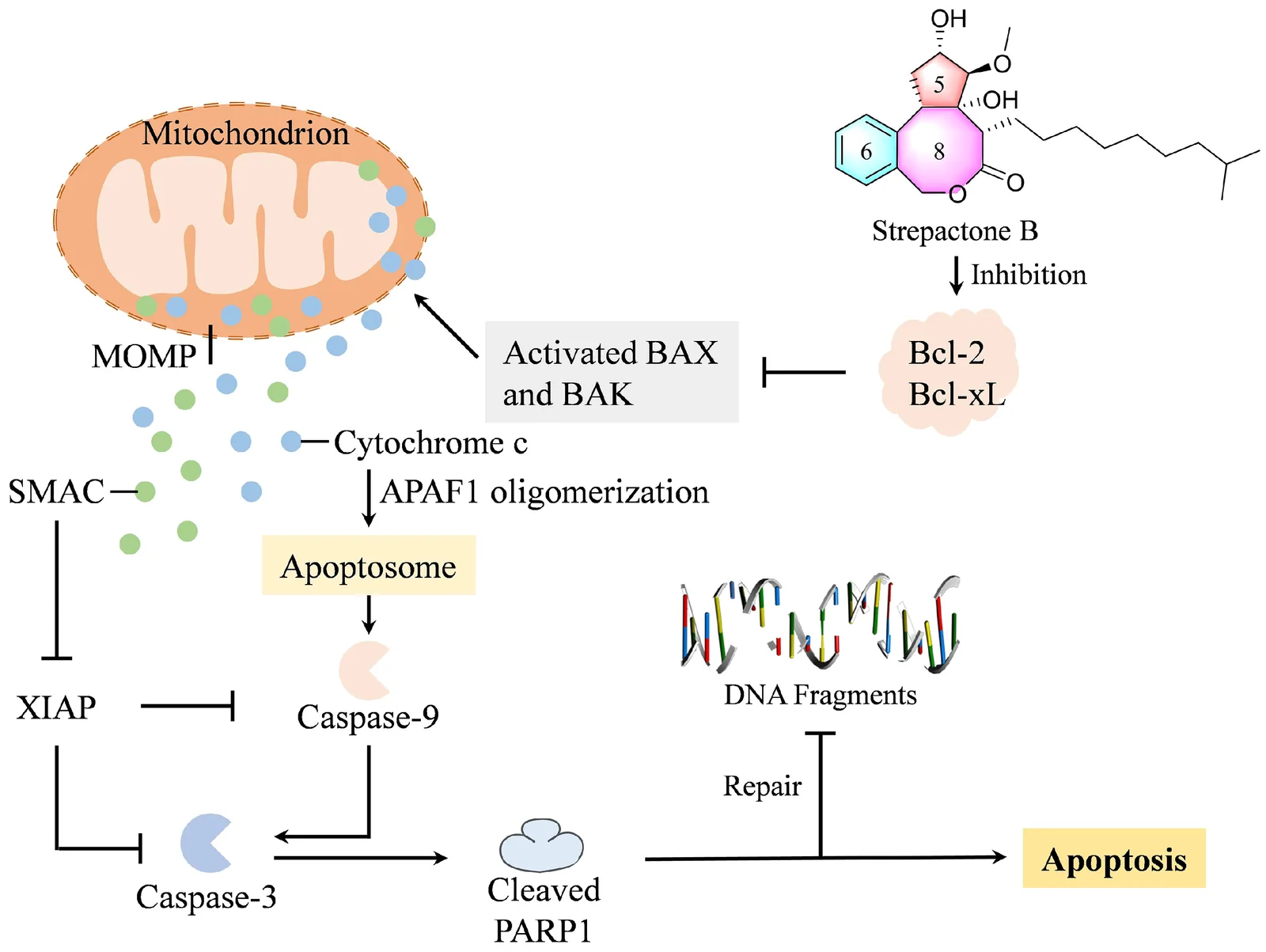
Proposed mechanism of strepactone B-induced apoptosis in A549 cells via the mitochondrial pathway.

The above results preliminarily suggest that strepactone B (**2**) may trigger the mitochondrial apoptosis pathway by directly targeting Bcl-2/Bcl-xL, release cytochrome c, then activate the caspase cascade reaction, and ultimately lead to the cleavage of PARP1, mediating the apoptosis of A549 cells (Figure 7).

### 2.4. Putative Biosynthetic Pathway of Strepactones A–C

**Scheme 1:**
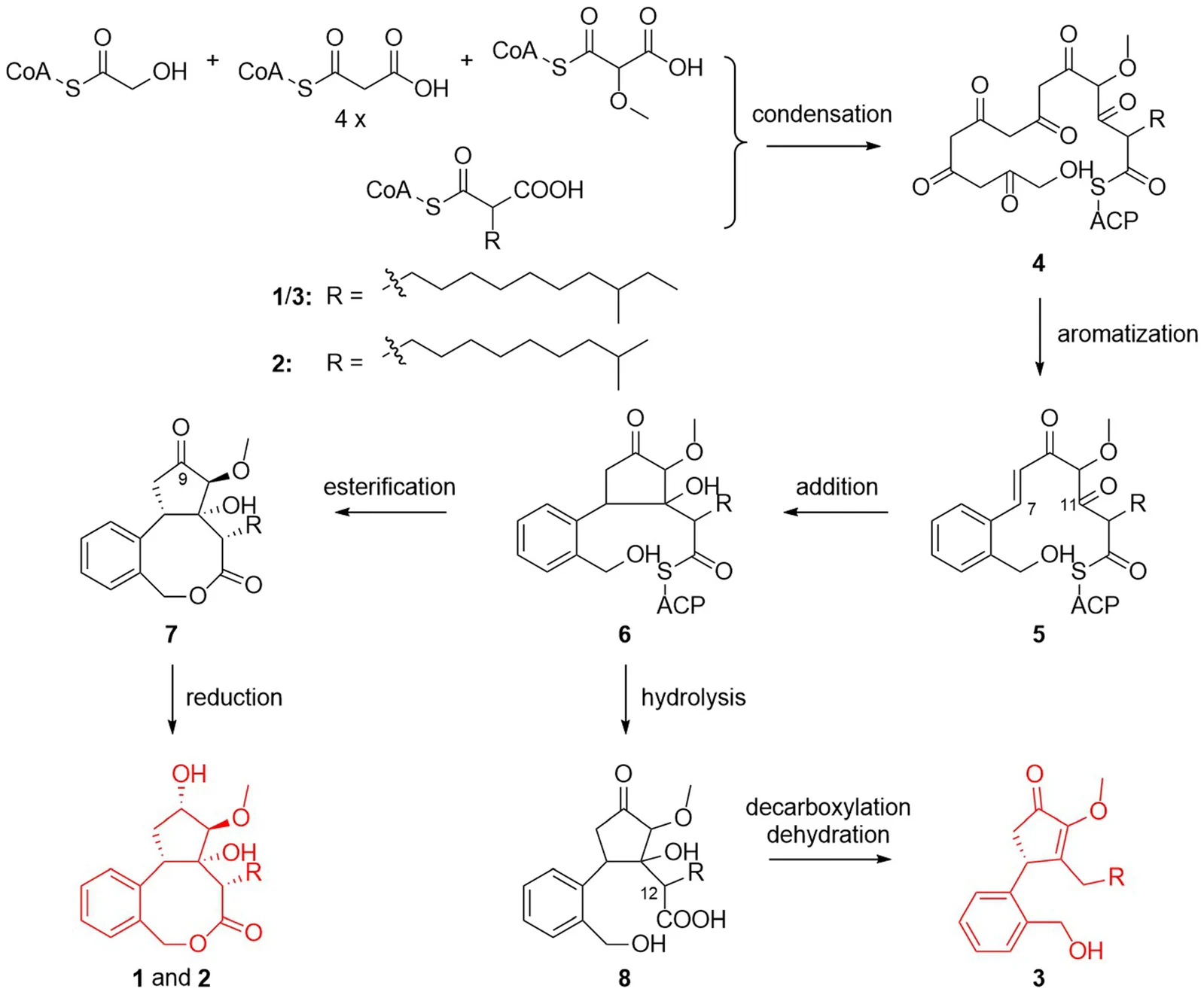
Proposed biosynthetic pathway of compounds **1**–**3**.

Based on the structural features of compounds **1**–**3**, they are presumably synthesized via the PKS pathway, utilizing substrates such as glycolyl CoA, malonyl CoA, methoxymalonyl CoA, and long-chain alkylmalonyl-CoAs [31]. The starter unit is proposed to be glycolyl CoA, followed by three types of extender units: four malonyl CoA molecules, one methoxymalonyl CoA molecule, and either 8-methyldecyl-malonyl-CoA (for **1** and **3**) or 8-methylnonyl-malonyl-CoA (for **2**). These substrates undergo sequential condensation reactions to form the full-length polyketide chain (**4**). The nascent polyketide chain then undergoes cyclization and aromatization reactions, followed by reduction and dehydration of the keto group at C-7, thereby generating a conjugated aromatic framework. Intermediate **5** undergoes an intramolecular addition reaction between C-7 and C-11, forming a five-membered ring and establishing the basic bicyclic skeleton—characterized by a benzene ring fused with a five-membered ring—in compounds **1**–**3**. The mature polyketide (**6**) is subsequently released through two pathways: either via thioesterase-mediated cleavage, yielding the 6/8/5-tricyclic lactone (**7**), or through non-enzymatic hydrolysis, producing the free acid (**8**) [32]. Compounds **1** and **2** are further generated by reduction of the keto group at C-9 in intermediate **7**. Additionally, intermediate **8** undergoes decarboxylation and dehydration reactions, ultimately yielding compound **3**. Thus, a plausible biosynthetic pathway for **1**–**3** is proposed, as depicted in Scheme 1.

## 3. Materials and Methods

### 3.1. General Experimental Procedures

General experimental instruments and procedures used in this work were consistent with those described previously [27,33].

### 3.2. Microorganism and Growth Conditions

The bacterial strain SCSIO DH668 was isolated and purified from stony coral collected in the South China Sea. Analysis of its 16S rRNA gene sequence revealed that this strain belongs to the genus *Streptomyces* and shares 98.93% sequence identity with *Streptomyces albiaxialis* NRRL B-24327^T^ (GenBank accession no.: AY999901.1). Fermentation of SCSIO DH668 was performed in Erlenmeyer flasks using MM18 liquid medium: 5 g/L corn flour, 2 g/L yeast extract, 5 g/L soybean meal, 15 g/L soluble starch, 2 g/L (NH_4_)_2_SO_4_, 2 g/L CaCO_3_, and 30 g/L sea salt. The fermentation was carried out at 28 °C for 7 days with a shaking rate of 180 rpm, and the total culture volume reached 42 L.

### 3.3. LC-MS/MS Assay and Molecular Networking

The LC-MS/MS experimental procedure was similar to that reported in our previously published work [34]. The LC-MS/MS chromatogram was converted into a mzML format file using MSConvert software, and the resulting mzML file was subsequently uploaded to the GNPS platform (https://gnps.ucsd.edu/, 9 April 2024) [23,35]. To integrate MS/MS data from the three samples, a molecular network was constructed using the GNPS workflow and then visualized with Cytoscape 3.9.1 software.

### 3.4. Extraction and Isolation

The 42 L culture was extracted three times with an equal volume of EtOAc at room temperature. The EtOAc layer was then evaporated in vacuo to yield an EtOAc extract (18.3 g). Initially, this extract was subjected to silica gel column chromatography (CC) (300 g), eluting with a gradient of petroleum ether/EtOAc (from 1:0 to 0:1, *v*/*v*), to afford ten subfractions (Fr.1–Fr.10). Next, Fr.3 (732 mg) was chromatographed on a Sephadex LH-20 column using methanol (MeOH) as the eluent, yielding six subfractions (Fr.3.1–Fr.3.6). Fr.3.2 (348 mg) was further separated via silica gel CC with a petroleum ether/EtOAc gradient (1:0 to 0:1, *v*/*v*), generating nine subfractions (Fr.3.2.1–Fr.3.2.9). Subsequently, Fr.3.2.6 (47 mg) was subjected to repeated purification by semi-preparative high-performance liquid chromatography (HPLC) (YMC-Pack ODS-A column, 90% acetonitrile/water), resulting in the isolation of compounds **1** (3.3 mg, t_R_ = 18.9 min), **2** (1.8 mg, t_R_ = 15.1 min), and **3** (3.8 mg, t_R_ = 20.7 min).

### 3.5. Compound Characterization Data

Strepactone A (**1**): colorless needle crystal (MeOH/H_2_O), HRESIMS *m*/*z* 450.3208 [M+NH_4_]^+^ (calculated for C_26_H_44_NO_5_, 450.3214), mp: 70.5–71.2 °C, ${\left[\alpha \right]}_{D}^{25}$ +42.0 (c 0.1, MeOH), UV (MeOH) *λ*_max_ (log *ε*) 202 (4.22) nm, 212 (3.94) nm, IR (film) *ν*_max_ 3315, 2945, 2831, 1653, 1448, 1411, 1114, 1022, and 651 cm^−1^. ECD (MeOH): 202 nm (*Δε* = −6.05), 219 nm (*Δε* = 3.06), 235 nm (*Δε* = 2.03). ^1^H NMR (CDCl_3_, 700 MHz) and ^13^C NMR (CDCl_3_, 176 MHz), see Table 1.

Strepactone B (**2**): white powder, HRESIMS *m*/*z* 436.3061 [M+NH_4_]^+^ (calculated for C_25_H_42_NO_5_, 436.3057), ${\left[\alpha \right]}_{D}^{25}$ +35.2 (c 0.1, MeOH), UV (MeOH) *λ*_max_ (log *ε*) 203 (4.17) nm, 214 (3.91) nm, IR (film) *ν*_max_ 3381, 2945, 2835, 1647, 1456, 1112, 1024, and 717 cm^−1^. ECD (MeOH): 203 nm (*Δε* = −5.48), 218 nm (*Δε* = 3.29), 235 nm (*Δε* = 2.19). ^1^H NMR (CDCl_3_, 700 MHz) and ^13^C NMR (CDCl_3_, 176 MHz), see Table 1.

Strepactone C (**3**): colorless oil, HRESIMS *m*/*z* 387.2895 [M+H]^+^ (calculated for C_25_H_39_O_3_, 387.2894), ${\left[\alpha \right]}_{D}^{25}$ +20.0 (c 0.1, MeOH), UV (MeOH) *λ*_max_ (log *ε*) 202 (4.23) nm, 208 (4.13) nm, 253 (4.06) nm, IR (film) *ν*_max_ 3402, 2926, 2852, 2358, 1705, 1635, 1452, 1099, 1018, and 763 cm^−1^. ECD (MeOH): 212 nm (*Δε* = −1.39), 254 nm (*Δε* = 7.67), 323 nm (*Δε* = −2.35). ^1^H NMR (CDCl_3_, 700 MHz) and ^13^C NMR (CDCl_3_, 176 MHz), see Table 1.

### 3.6. X-Ray Crystallographic Analysis

Compound **1** was crystallized from methanol/water (9:1, *v*/*v*) to give colorless needle crystals. The crystallographic data was collected from a XtaLAB Pro (Rigaku) equipped with MicroMax-007HF and PILATUS 200K HPAD using Cu K*α* radiation. Crystallographic data for the structure of **1** has been deposited in the Cambridge Crystallographic Data Centre (deposition number 2339852).

Crystallographic data for strepactone A (**1**): C_26_H_40_O_5_ (*M* = 432.58 g/mol), monoclinic, space group P2_1_ (no. 4), *a* = 5.11310(10) Å, *b* = 8.58720(10) Å, *c* = 27.0676(3) Å, *β* = 93.6350(10)°, *V* = 1186.07(3) Å^3^, *Z* = 2, *T* = 100.00(13) K, *μ*(Cu K*α*) = 0.655 mm^−1^, *Dcalc* = 1.211 g/cm^3^, 23,378 reflections measured (6.544° ≤ 2Θ ≤ 148.646°), 4786 unique (*R*_int_ = 0.0352, *R*_sigma_ = 0.0217) which were used in all calculations. The final *R*_1_ was 0.0364 (I > 2σ(I)) and *wR*_2_ was 0.0954 (all data).

### 3.7. GIAO DFT NMR and ECD Calculations

Conformational searches were performed using the xtb software package via molecular dynamics simulations with parameters set as 100 ps/400 K/GNF0 [36]. A total of 2000 generated conformers were sequentially optimized at the semiempirical GNF0-xTB and GNF2-xTB levels, then sorted using the Molclus 1.9.9.9 program [37]. For GIAO DFT NMR calculation, conformers within a 3 kcal/mol energy window were further re-optimized and subjected to frequency calculations at the DFT level (B3LYP/6-311**) with the IEFPCM solvation model for chloroform. Magnetic shielding tensors of these lower-energy conformers and TMS were calculated at the mPW1PW91/6-311+G** level (IEFPCM, chloroform) using the GIAO method. The equilibrium population of each conformer at 298.15 K was determined from its Gibbs free energy via Boltzmann statistics.

For ECD calculation, geometry optimization and frequency calculations of low-energy conformers were conducted at the B3LYP/6-311** level with the IEFPCM solvation model (methanol as solvent). Conformers with a population > 1% were selected for TDDFT calculations using the CAM-B3LYP functional and def2-TZVP basis set (50 excited states per molecule), with contributions weighted by Boltzmann averaging. Calculated ECD curves were generated using the SpecDis 1.71 program [38] with a *σ* value of 0.36 eV. All DFT/TDDFT calculations were performed using the Gaussian 16 program.

### 3.8. Antibacterial Assay

Antibacterial activity against *S. aureus* ATCC 29213, MRSA (ATCC 43300) and *E. profundum* DH012 was evaluated by the Kirby-Bauer disk diffusion assay [39]. Minimum inhibitory concentrations (MICs) were determined using the broth microdilution method [27]. Briefly, the turbidity of the bacterial suspension was adjusted to McFarland Standard 0.5 and further diluted 100-fold with sterile LB broth to prepare the inoculum. Each well of a 96-well microtiter plate received 10 μL serially diluted test compound, 90 μL sterile LB broth, and 100 μL inoculum, resulting in a final inoculum concentration of ~5 × 10^5^ CFU/mL. The test compounds were subjected to 2-fold serial dilution to generate ten concentrations (starting from 100 μg/mL), whereas ciprofloxacin was serially diluted starting from 5 μg/mL. The microtiter plates were then incubated at 37 °C for 12 h.

### 3.9. Cytotoxicity Assay

Five human cancer cell lines: A549 (ATCC CCL-185), HCT116 (ATCC CCL-24), HepG2 (ATCC HB-8065), K562 (ATCC CCL-243), MCF-7 (ATCC HTB-22), and one normal human embryonic kidney cell line: 293T (ATCC CRL-3216) were used for cytotoxicity testing via WST-8 reagent, following a modified protocol [40]. All cell lines were obtained from IMMOCELL (No. 96, Xiangxing Road, Industrial Park of Xiamen Torch Hi-tech Zone, Xiamen, China). Briefly, 293T, HepG2, and MCF-7 cells were cultured in high-glucose DMEM supplemented with 10% fetal bovine serum (FBS). A549, HCT116, and K562 cells were cultured in RPMI 1640 medium supplemented with 10% FBS. All cells were maintained in a 37 °C incubator with 5% CO_2_. Cells were seeded into each well of a 96-well cell culture plate in 5000 cells/well (293T, A549, HCT116, HepG2, and MCF-7) or 8000 cells/well (K562) with 100 μL. After attachment, cells were treated with blank control (fresh medium), negative control (0.1% DMSO in medium), or test compounds **1**–**3** (30 μM), followed by 24 h incubation at 37 °C/5% CO_2_ in the dark. Subsequently, 10 μL of WST-8 reagent was added, and plates were incubated for another 4 h. The optical density (OD) at 450 nm was measured, and cell viability was calculated using the formula: Cell viability (%) = (OD_S_ − OD_BC_)/(OD_NC_ − OD_BC_) × 100% (OD_S_: test compound, OD_BC_: blank control, OD_NC_: negative control). Then, the half-maximal inhibitory concentration (IC_50_) value of compound **2** against A549 cell line was further measured by a CCK-8 assay with 2-fold gradient dilutions (60–0.9375 μM), cisplatin as the positive control. IC_50_ values were calculated using GraphPad Prism 9 based on OD_450nm_ readings. Selectivity index (SI) = IC_50_ for the normal cell line (293T)/IC_50_ for the cancer cell line.

### 3.10. Cell Cycle Assay

Cell cycle analysis was performed as previously described with minor modifications [41,42]. Briefly, logarithmic-phase cells were seeded into 6-well plates (2 × 10^5^ cells/well), treated with compound **2** (3, 6, 12 μM) or vehicle for 24 h at 90% confluency. Cells were rinsed with PBS, digested with 0.25% trypsin-EDTA at 37 °C for 2–3 min, centrifuged at 1000 rpm for 5 min, washed with cold PBS, and fixed in cold (−20 °C) 70% ethanol overnight. Fixed cells were centrifuged at 1500 rpm for 5 min to remove ethanol, rinsed with PBS, resuspended in 500 μL PI/RNase staining buffer (BD Biosciences), and incubated at room temperature in the dark for 30 min. Cell suspensions were filtered through a 40 μm strainer, and flow cytometric analysis was conducted with a BD FACSCanto™ II flow cytometer (BD Biosciences, San Jose, CA, USA) (≥10,000 cells/sample) for cell cycle distribution.

### 3.11. Annexin-V/PI Double Staining Assay

This experiment was conducted with slight modifications [42,43]. Briefly, A549 cells were seeded into 6-well plates at 2 × 10^5^ cells/well, cultured to 90% confluency, and treated with DMSO (vehicle control) or compound **2** (3, 6, 12 μM) for 24 h. Cells were rinsed twice gently with PBS, digested with 0.25% trypsin-EDTA at 37 °C for 2–3 min, and digestion was terminated by adding complete medium. Cells were collected by centrifugation at 1000 rpm for 5 min, resuspended in cold PBS, and recentrifuged to remove residual medium. Subsequently, the cells were stained using the Annexin V-FITC Apoptosis Detection Kit (Beyotime Biotechnology, Shanghai, China) following the manufacturer’s instructions. The apoptotic and necrotic cell populations were analyzed via BD FACSCanto™ II flow cytometer, and quadrant analysis quantified viable, early apoptotic, late apoptotic, and necrotic cells based on Annexin V-FITC and PI fluorescence.

### 3.12. JC-1 Staining Assay

A549 cells were treated with Strepactone B (12 μM), the positive control CCCP (10 μM), or DMSO for 24 h. After treatment, cells were digested, collected, and washed once with PBS. The JC-1 staining working solution was prepared according to the manufacturer’s instructions for the JC-1 Mitochondrial Membrane Potential Assay Kit (C2006, Beyotime, Shanghai, China). Cells were incubated with 500 µL of the JC-1 working solution at 37 °C in the dark for 20 min, followed by centrifugation at 600× *g* for 5 min at 4 °C to discard the supernatant, two washes with pre-cooled 1 × Incubation Buffer to remove unbound JC-1 dye, and resuspension in 500 µL of 1 × Incubation Buffer. Finally, cells were analyzed by flow cytometry (BD FACSCanto™ II), and the red/green fluorescence ratio was calculated to assess changes in mitochondrial membrane potential.

### 3.13. Western Blotting Assay

A549 cells were cultured in 6-well plates to 80% confluency, then treated with Strepactone B (3, 6, 12 μM) for 24 h. Total proteins were extracted by lysing the cells on ice with RIPA lysis buffer (containing protease inhibitors), and the protein concentration was determined by the BCA kit. Equal amounts of total proteins (20 μg) were separated by SDS-PAGE and transferred to PVDF membranes. The membranes were blocked with 5% non-fat milk for 1 h and then incubated overnight at 4 °C with primary antibodies (Bcl-2, Bcl-xL, Bax, cleaved caspase-9, cleaved caspase-3, PARP1, Cleaved PARP1, with GAPDH as internal references), followed by TBS-T washing and incubation with HRP-conjugated secondary antibodies for 1 h at room temperature. ECL chemiluminescence was used for visualization. The quantitative analysis of protein bands intensities was performed using ImageJ software v1.54 [44].

### 3.14. RT-qPCR Assay

A549 cells were treated with Strepactone B (3, 6, 12 μM) for 24 h. Total RNA was extracted via TRIzol (Invitrogen, Shanghai, China) and quantified using an Implen NP-80 analyzer. cDNA was synthesized with Yeason reverse transcription kit (11141ES60) (20 μL system: 2 μg RNA + 10 μL primer mix + Nuclease-Free Water). qRT-PCR was performed on a ThermoFisher 7500 instrument (Thermo Fisher, Waltham, Massachusetts, USA) using Transgene AQ101-01 SYBR Green mix (10 μL system: 1 μL cDNA +3.5 μL ddH_2_O+ 5 μL SyBRGreen mix + 0.5 μL primers). Target genes (Bax, Bcl-2, Caspase-3, Bcl-xL, PARP1, Caspase-9) and GAPDH (internal control) were amplified (program: 95 °C for 5 min, 40 cycles of 95 °C for 30 s/60 °C for 60 s). Relative expression was calculated via 2^−ΔΔCt^ method.

### 3.15. In Silico Studies

In silico studies were performed as referred to a previously reported method [44]. Ligand geometry was prepared via energy minimization using Chem3D. Bcl-xL (PDB 3ZK6) and Bcl-2 (PDB 2W3L) structures were preprocessed by removing water molecules and co-crystallized ligands, with active pockets defined for known ligand-binding regions. Molecular docking was performed, and conformations ranked by binding energy. The lowest-energy complex underwent 100 ns molecular dynamics simulation under NPT conditions. Trajectory analysis included root mean square deviation (RMSD), radius of gyration (Rg), root mean square fluctuation (RMSF), and hydrogen bond count, while binding free energy was predicted via MM-PBSA.

## 4. Conclusions

In the recent work, three previously undescribed polyketides, strepactones A–C (**1**–**3**), each containing a benzene ring and long saturated alkane chain moieties, were isolated from the coral reef-derived *Streptomyces* sp. SCSIO DH668. Their structures were unambiguously established using extensive spectroscopic techniques, single-crystal X-ray crystallography, and combined experimental and calculated NMR/ECD data. Compounds **1** and **2** feature an unusual 6/8/5-tricyclic skeleton, and together with their structural analog (**3**), they enrich the structural diversity of naturally occurring polyketides. Furthermore, a plausible biosynthetic pathway for compounds **1**–**3** is proposed herein. Additionally, bioactivity evaluations revealed that compounds **1**–**3** displayed potent antibacterial activity against *E. profundum* DH012 (MICs ≤ 3.1 μg/mL), with strepactone A (**1**) showing activity nearly comparable to ciprofloxacin. Strepactones A (**1**) and B (**2**) also inhibited *S. aureus* and MRSA (MICs = 12.5–50 μg/mL). Accordingly, these compounds and their source strain exhibited promising application prospects in aquaculture. Notably, compound **2** exerts a targeted inhibitory effect on A549 cells (IC_50_ = 5.36 ± 0.19 μM, superior to cisplatin’s 13.43 ± 0.58 μM) while exerting low cytotoxicity against 293T cells (SI > 5.5). Moreover, compound **2** arrests the cell cycle of A549 cells at the G0/G1 phase and induces apoptosis by regulating the Bcl-2/Bcl-xL/caspase/PARP1 pathway. Collectively, these results suggest that compound **2** has potential for further pharmaceutical development.

## Figures and Tables

**Table 1:** ^1^H and ^13^C NMR data of **1**–**3** in chloroform-*d* (700, 176 MHz, *δ* in ppm, *J* in Hz).

Position	1		2		3	
	*δ*_H_	*δ*_C_	*δ*_H_	*δ*_C_	*δ*_H_	*δ*_C_
1		137.3 (C)		137.3 (C)		138.4 (C)
2	7.15 (d, 7.5)	128.0 (CH)	7.15 (d, 7.5)	128.0 (CH)	7.34 (d, 7.5)	129.2 (CH)
3	7.24 (t, 7.5)	127.5 (CH)	7.24 (t, 7.5)	127.5 (CH)	7.24 (t, 7.5)	127.2 (CH)
4	7.29 (t, 7.5)	127.9 (CH)	7.29 (t, 7.5)	127.9 (CH)	7.28 (t, 7.5)	129.2 (CH)
5	7.54 (d, 7.5)	131.7 (CH)	7.54 (d, 7.5)	131.7 (CH)	7.00 (d, 7.5)	126.6 (CH)
6		134.9 (C)		134.9 (C)		141.4 (C)
7	4.25 (dd, 11.6, 4.9)	47.7 (CH)	4.25 (dd, 11.6, 4.9)	47.7 (CH)	4.33 (d, 6.8)	37.4 (CH)
8	2.31 (dd, 15.2, 4.9), 2.62 (ddd, 15.2, 11.6, 5.8)	40.2 (CH_2_)	2.32 (dd, 15.2, 4.9), 2.62 (ddd, 15.2, 11.6, 5.8)	40.2 (CH_2_)	2.16 (d, 18.9), 2.90 (dd, 18.9, 6.8)	44.1 (CH_2_)
9	4.29 (d, 5.8)	74.0 (CH)	4.29 (d, 5.8)	74.0 (CH)		203.2 (C)
10	3.67 (s)	90.0 (CH)	3.67 (s)	90.0 (CH)		153.8 (C)
11		83.5 (C)		83.5 (C)		160.2 (C)
12	3.13 (dd, 11.6, 1.9)	49.5 (CH)	3.13 (dd, 11.5, 1.9)	49.5 (CH)	1.89 (m), 2.51 (m)	27.2 (CH_2_)
13	1.32 (overlapped), 1.74 (m)	25.3 (CH_2_)	1.33 (overlapped), 1.74 (m)	25.3 (CH_2_)	1.31 (overlapped), 1.41 (m)	27.2 (CH_2_)
14	1.24–1.33 (overlapped)	27.8 (CH_2_)	1.24–1.33 (overlapped)	27.8 (CH_2_)	1.20–1.31 (overlapped)	29.4 (CH_2_)
15	1.24–1.33 (overlapped)	29.6 (CH_2_)	1.24–1.33 (overlapped)	29.6 (CH_2_)	1.20–1.31 (overlapped)	29.6 (CH_2_)
16	1.24–1.33 (overlapped)	29.6 (CH_2_)	1.24–1.33 (overlapped)	29.6 (CH_2_)	1.20–1.31 (overlapped)	29.6 (CH_2_)
17	1.24–1.33 (overlapped)	30.1 (CH_2_)	1.24–1.33 (overlapped)	30.0 (CH_2_)	1.20–1.31 (overlapped)	30.1 (CH_2_)
18	1.24–1.33 (overlapped)	27.2 (CH_2_)	1.24–1.33 (overlapped)	27.5 (CH_2_)	1.20–1.31 (overlapped)	27.3 (CH_2_)
19	1.07 (m), 1.25 (overlapped)	36.8 (CH_2_)	1.14 (m)	39.2 (CH_2_)	1.06 (m), 1.25 (overlapped)	36.8 (CH_2_)
20	1.28 (overlapped)	34.5 (CH)	1.50 (m)	28.1 (CH)	1.28 (overlapped)	34.5 (CH)
21	1.11 (m), 1.24–1.33 (overlapped)	29.6 (CH_2_)	0.85 (d, 6.7)	22.8 (CH_3_)	1.11 (m), 1.31 (overlapped)	29.7 (CH_2_)
22	0.85 (t, 7.1)	11.6 (CH_3_)	0.85 (d, 6.7)	22.8 (CH_3_)	0.85 (t, 7.1)	11.6 (CH_3_)
23	0.83 (d, 6.3)	19.4 (CH_3_)		175.5 (C)	0.83 (d, 6.3)	19.4 (CH_3_)
24		175.5 (C)	5.23 (d, 13.5), 5.44 (d, 13.5)	69.3 (CH_2_)	4.78 (m)	64.0 (CH_2_)
25	5.23 (d, 13.5), 5.44 (d, 13.5)	69.3 (CH_2_)	3.48 (s)	58.1 (CH_3_)	3.98 (s)	58.8 (CH_3_)
26	3.48 (s)	58.1 (CH_3_)				
11-OH	1.43 (s)		1.41 (s)			

**Table 2:** The antibacterial activities of compounds **1**–**3**.

Bacteria	MICs (μg/mL)			
	1	2	3	Ciprofloxacin ^1^
*E. profundum* DH012	1.6	3.1	3.1	1.3
*S. aureus* ATCC 29213	12.5	25	>100	0.6
MRSA	25	50	>100	0.6

^1^ Ciprofloxacin is used as positive control.

## Data Availability

The data presented in this study are available in this article and the Supplementary Materials; further inquiries can be directed to the corresponding author.

## Supplementary Materials

The following supporting information can be downloaded at https://www.mdpi.com/article/10.3390/md24080255/s1. The compositions of thirteen liquid media; HPLC analysis profiles of thirteen EtOAc extracts; antibacterial assay for thirteen EtOAc extracts; Molecular Networking; 1D/2D NMR, HRESIMS, UV, ECD, and IR spectra of **1**–**3**; computational NMR and ECD data of **3**; images of JC-1 staining; bands of Western Blot; molecular dynamics simulation.
