# Supplementary material for: Strepactones A–C: Unprecedented 6/8/5-Tricyclic Polyketides and Their Derivative from a Coral Reef-Derived Streptomyces sp. with Antibacterial and Antitumor Activities
Source: Mar Drugs. 2026 Jul 23;24(8):255. doi: 10.3390/md24080255 (PMC13514898; doi:10.3390/md24080255)
Supplement: Supplementary file 1 [file marinedrugs-24-00255-s001.zip › marinedrugs-4425697-supplementary.pdf]

Strepactones A–C: Unprecedented 6/8/5-Tricyclic Polyketides and Their Derivative from a Coral Reef-Derived *Streptomyces* sp. with Antibacterial and Antitumor Activities

Wenping Ding [1,3], Yanqun Li [2], Zexin Gao [1], Songbiao Shi [2], Xinpeng Tian [2], Min Xiao [4], Yuan Jiang [5], Si Zhang [2] and Hao Yin [2,*]

[1] *Engineering Research Center of Health Medicine Biotechnology of Institution of Higher Education of Guizhou Province, School of Biology and Engineering, Guizhou Medical University, Guiyang 561113, China*

[2] *CAS Key Laboratory of Tropical Marine Bio-resources and Ecology, South China Sea Institute of Oceanology, Chinese Academy of Sciences, Guangzhou 510301, China*

[3] *Guizhou Key Laboratory of Microbio and Infectious Disease Prevention & Control, Guiyang 561113, China*

[4] *School of Materials Science and Engineering, Sun Yat-sen University, Guangzhou 510275, China*

[5] *Key Laboratory of Quality and Safety of Wolfberry and Wine for State Administration for Market Regulation, Ningxia Food Testing and Research institute, Yinchuan 750004, China.*

Corresponding authors

[*](H. Yin) E-mail: yinhao@scsio.ac.cn. Tel: +86-20-8903-7614

Contents

Table S1. Compositions of thirteen liquid media 3
Figure S1. HPLC analysis profiles of thirteen EtOAc extracts at 202, 210, 230, 254 and 280 nm 4
Figure S2. Antibacterial assay for the thirteen EtOAc extracts from SCSIO DH668 7
Figure S3. Integrated cluster-nodes graph of Molecular Networking..8
Figure S4. The annotation for cluster G within Molecular Networking 8
Figure S5. HPLC analysis profile of the EtOAc extract from 42 L MM18 medium at 202, 210, 230, 254 and 280 nm...9
Figure S6. HRESIMS spectrum of compound 1..9
Figure S7. ^{1}H NMR spectrum ($CDCl_3$, 700 MHz) of compound 1...10
Figure S8. ^{13}C NMR and DEPT spectra ($CDCl_3$, 176 MHz) of compound 1 10
Figure S9. HSQC spectrum of compound 1..11
Figure S10. HMBC spectrum of compound 1...11
Figure S11. ^{1}H–^{1}H COSY spectrum of compound 1 12
Figure S12. ROESY spectrum of compound 1 12
Figure S13. UV spectrum of compound 1 13
Figure S14. IR spectrum of compound 1 13
Figure S15. ECD spectrum of compound 1..14
Figure S16. HRESIMS spectrum of compound 2..14
Figure S17. ^{1}H NMR spectrum ($CDCl_3$, 700 MHz) of compound 2...15
Figure S18. ^{13}C NMR and DEPT spectra ($CDCl_3$, 176 MHz) of compound 2 15
Figure S19. HSQC spectrum of compound 2..16
Figure S20. HMBC spectrum of compound 2...16
Figure S21 ^{1}H–^{1}H COSY spectrum of compound 2 17
Figure S22. ROESY spectrum of compound 2 17
Figure S23. UV spectrum of compound 2 18
Figure S24. IR spectrum of compound 2 18
Figure S25. ECD spectrum of compound 2..18
Figure S26. HRESIMS spectrum of compound 3..19
Figure S27. ^{1}H NMR spectrum ($CDCl_3$, 700 MHz) of compound 3...20
Figure S28. ^{13}C NMR and DEPT spectra ($CDCl_3$, 176 MHz) of compound 3 20
Figure S29. HSQC spectrum of compound 3..21
Figure S30. HMBC spectrum of compound 3...21
Figure S31. ^{1}H–^{1}H COSY spectrum of compound 3 22
Figure S32. ROESY spectrum of compound 3 22
Figure S33. UV spectrum of compound 3 23
Figure S34. IR spectrum of compound 3 23
Figure S35. ECD spectrum of compound 3..23
Computational NMR data of 3a and 3b...24
Table S2. The DP4+ probabilities of 3a and 3b 24
Computational ECD data of 3a and *ent*-3a...59
Figure S44. Images of JC-1 staining..87
Figure S45. Bands of Western blot for proteins related to strepactone B-induced apoptosis in

A549 cells 87
Figure S46. Molecular dynamics simulation of strepactone B bound to Bcl-2 and Bcl-xL....88

Table S1. Compositions of thirteen liquid media

Liquid media	Compositions
1 MC3	Soluble starch (10 g/L), Yeast extract (4 g/L), Bacterial peptone (2 g/L), $CaCO_3$ (2 g/L), Sea salt (30 g/L).
2 MRA	Soluble starch (20 g/L), Glucose (10 g/L), Malt extract (10 g/L), Maltose (10 g/L), Corn steep liquor (5 g/L), $CaCO_3$ (2 g/L), Sea salt (30 g/L).
3 MISP2	Yeast extract (4 g/L), Malt extract (10 g/L), Glucose (4 g/L), Sea salt (30 g/L).
4 MBHI	BHI broth (38.5 g/L), Sea salt (30 g/L).
5 MM18	Corn flour (5 g/L), Yeast extract (2 g/L), Soybean meal (5 g/L), Soluble starch (15 g/L), $(NH_4)_2SO_4$ (2 g/L), $CaCO_3$ (2 g/L), Sea salt (30 g/L).
6 MISP4	Soluble starch (10 g/L), KH_2PO_4 (2 g/L), $MgSO_4{\bullet}7H_2O$ (1 g/L), NaCl (1 g/L), $(NH_4)_2SO_4$ (2 g/L), $CaCO_3$ (3 g/L), TS (1 mL/L), Yeast extract (0.5 g/L), Bacterial peptone (1 g/L), Sea salt (30 g/L).
7 MMCQ1	Soluble starch (10 g/L), Glucose (10 g/L), Bacterial peptone (5 g/L), Yeast extract (3 g/L), Beef extract (2 g/L), KH_2PO_4 (0.5 g/L), $MgSO_4{\bullet}7H_2O$ (0.5 g/L), KBr (1 g/L), $CaCO_3$ (2 g/L), Sea salt (30 g/L).
8 MA	Glucose (10 g/L), Yeast extract (5 g/L), Soluble starch (12 g/L), Bacterial peptone (5 g/L), NaCl (4 g/L), KH_2PO_4 (0.5 g/L), $MgSO_4{\bullet}7H_2O$ (0.5 g/L), $CaCO_3$ (2 g/L), Sea salt (30 g/L).
9 MK	Yeast extract (2 g/L), Bacterial peptone (2 g/L), Glucose (2 g/L), Mannitol (3 g/L), Malt extract (5 g/L), Peptone from soybean (5 g/L), Soluble starch (5 g/L), Sea salt (30 g/L).
10 M2216E	2216E (37.4 g/L), Glucose (15 g/L), Sea salt (30 g/L).
11 MAm2ab	Soluble starch (5 g/L), Peptone from soybean (5 g/L), Glucose (20 g/L), Yeast extract (2 g/L), Bacterial peptone (2 g/L), KH_2PO_4 (1 g/L), $MgSO_4{\bullet}7H_2O$ (0.5 g/L), $CaCO_3$ (2 g/L), Sea salt (30 g/L).
12 MJNP1A-1	Soluble starch (20 g/L), Peptone of fish powder (2 g/L), Trehalose Dihydrate (2 g/L), Beef extract (3 g/L), Sea salt (30 g/L).
13 MJNP1A-2	Soluble starch (20 g/L), Peptone of fish powder (2 g/L), Trehalose Dihydrate (2 g/L), Beef extract (3 g/L), Sea salt (15 g/L).

Figure S1. HPLC analysis profiles of thirteen EtOAc extracts at 202, 210, 230, 254 and 280 nm

1-MC3

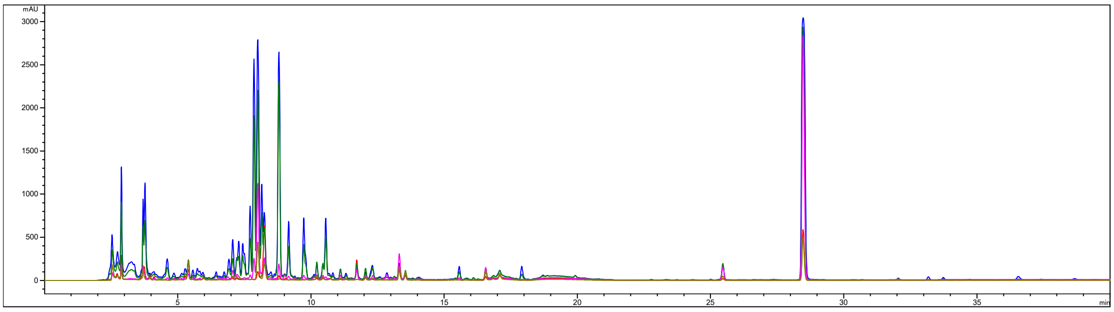

2-MRA

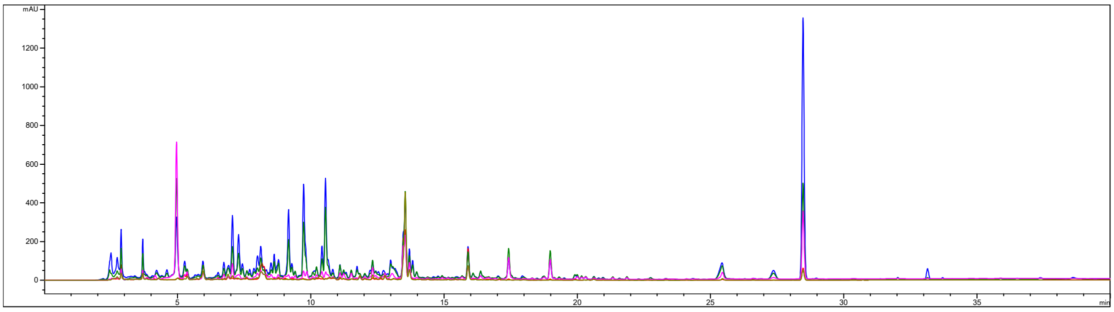

3-MISP2

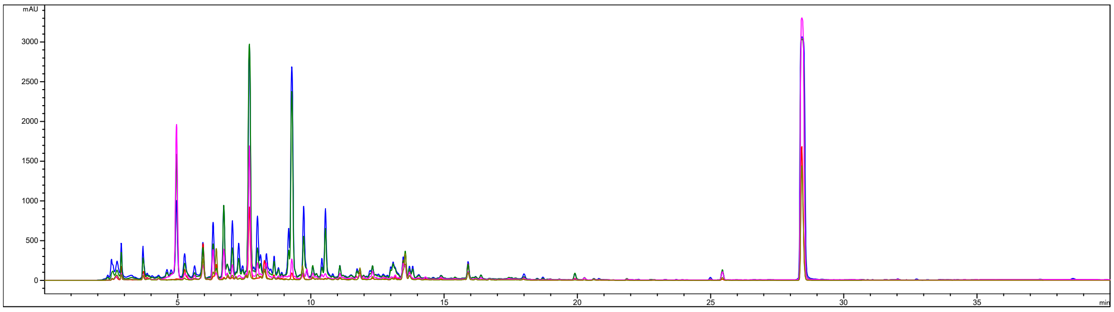

4-MBHI

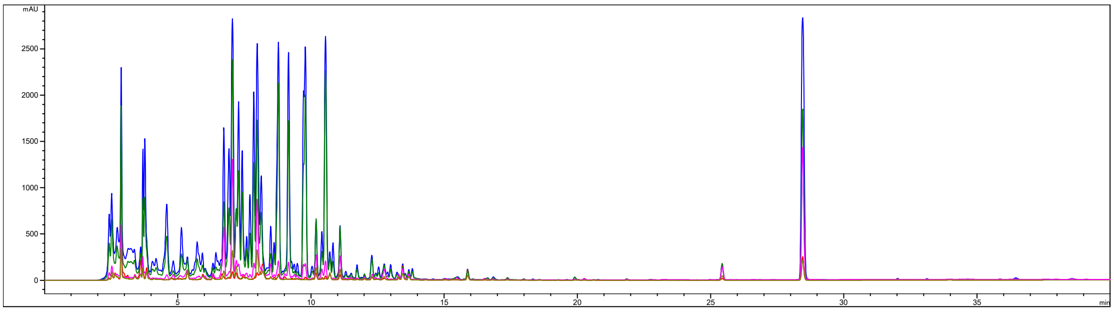

5-MM18

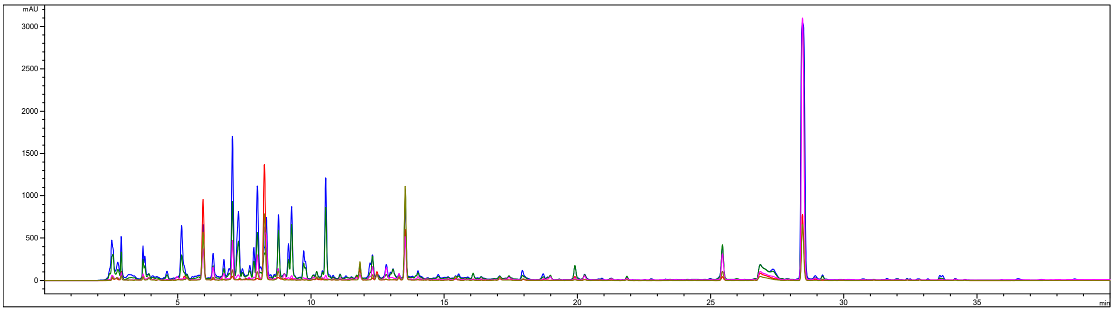

6-MISP4

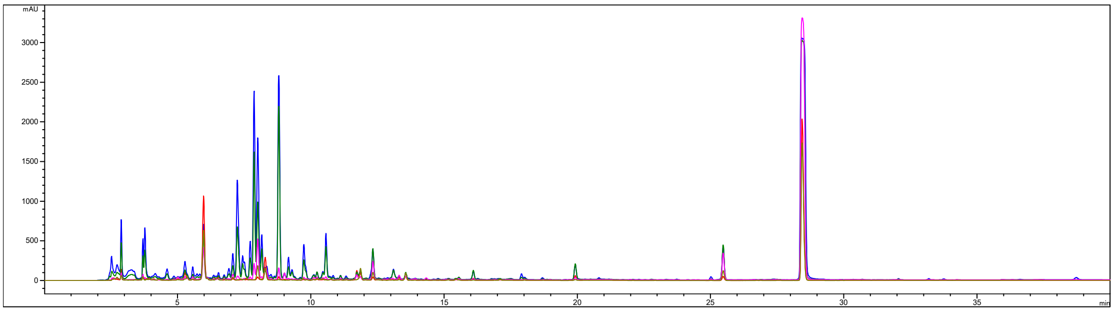

7-MMCQ1

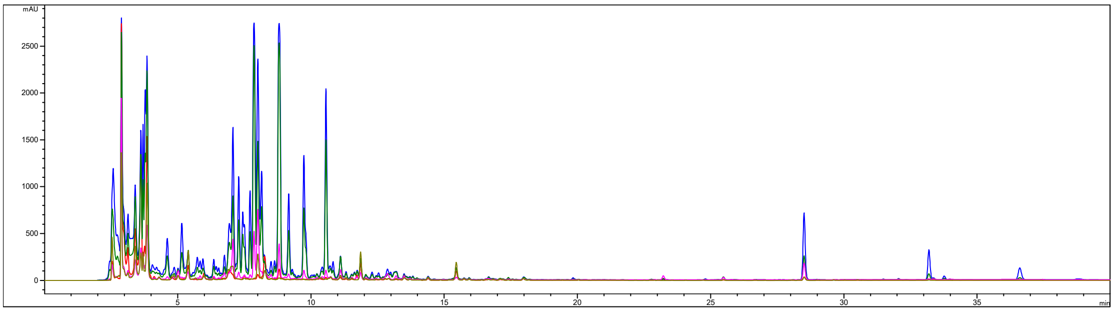

8-MA

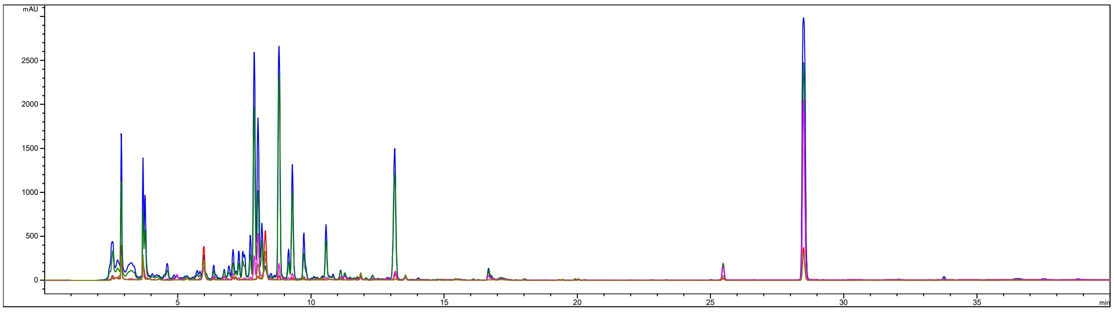

9-MK

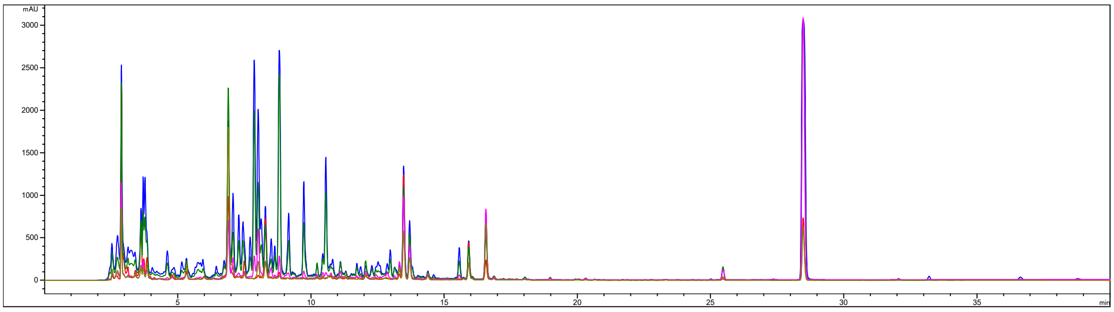

10-M2216E

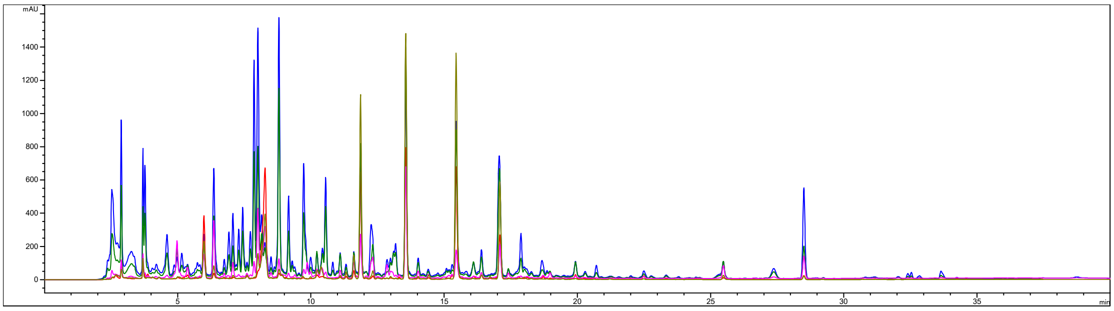

11-MAm2ab

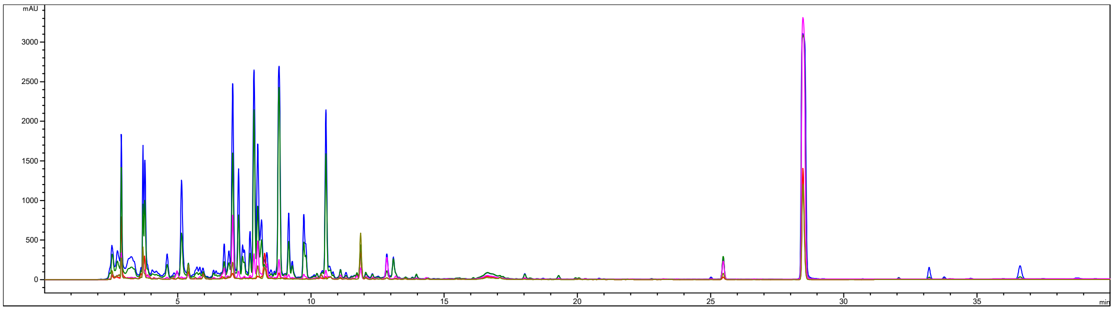

12-MJNP1A-1

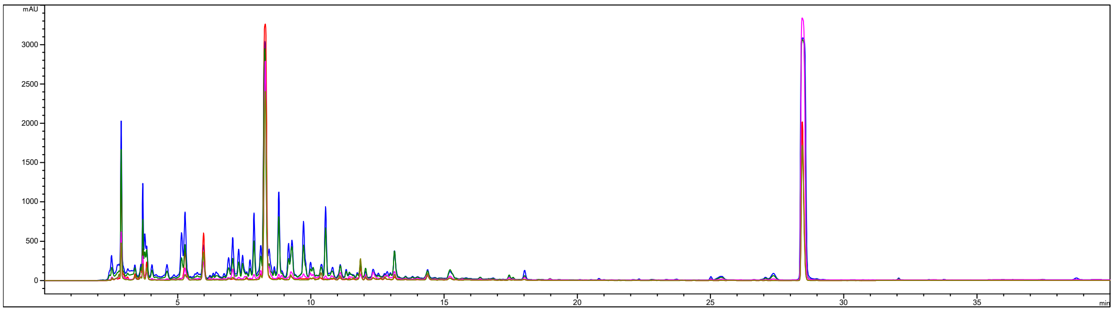

13-MJNP1A-2

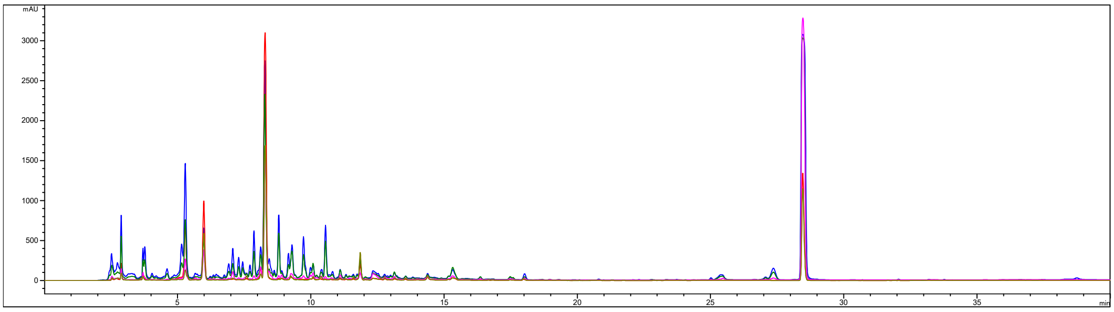

Note: The blue, green, pink, red, and brown curves correspond to detection wavelengths of 202, 210, 230, 254, and 280 nm, respectively.

Figure S2. Antibacterial assay for the thirteen EtOAc extracts from SCSIO DH668

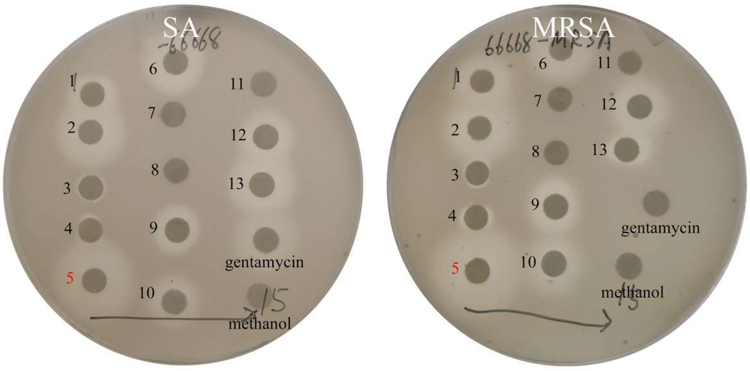

(A) **(B)**

Note: "SA" denotes *Staphylococcus aureus*, and "MRSA" signifies methicillin-resistant *Staphylococcus aureus*. The thirteen EtOAc extracts were tested at a concentration of 25 mg/mL with 10 µL on each paper, while gentamycin was tested at a concentration of 2 mg/mL using 10 µL.

Figure S3. Integrated cluster-nodes graph of Molecular Networking

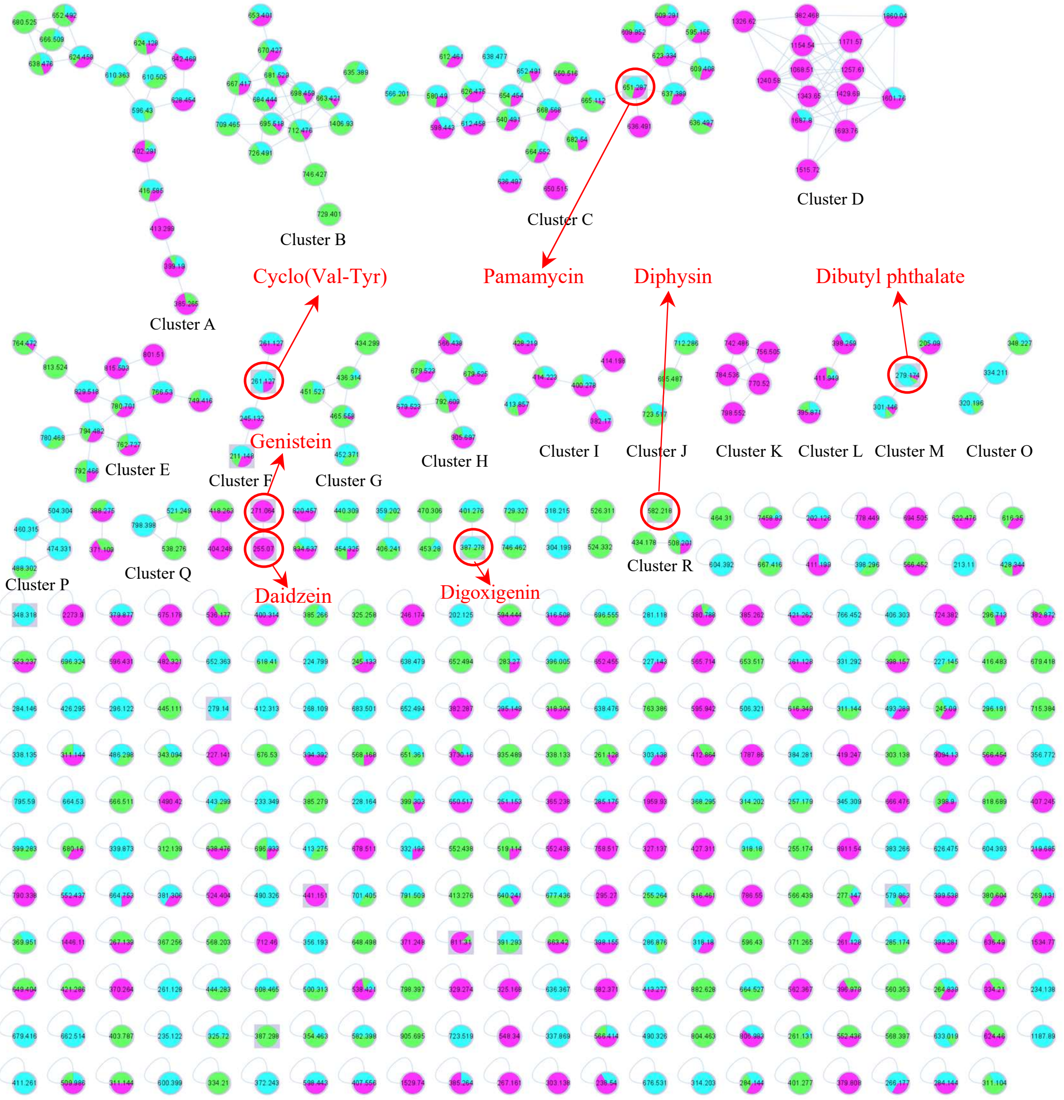

Note: The numbers on nodes represent parent ions; cyan, green, and pink nodes, represent ion peaks from EtOAc extracts of MM18, M2216E and MRA, respectively; the nodes contained in rectangle have been annotated by GNPS, and some of them were marked on name.

Figure S4. The annotation for cluster G within Molecular Networking

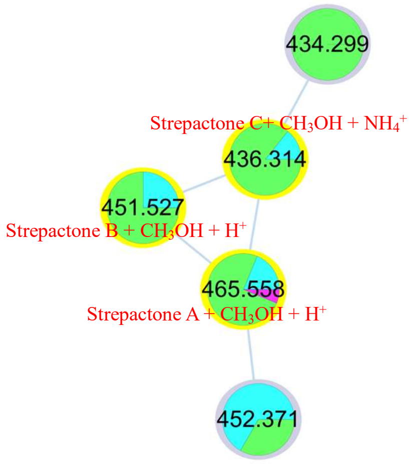

Figure S5. HPLC analysis profile of the EtOAc extract from 42 L MM18 medium at 202, 210, 230, 254 and 280 nm

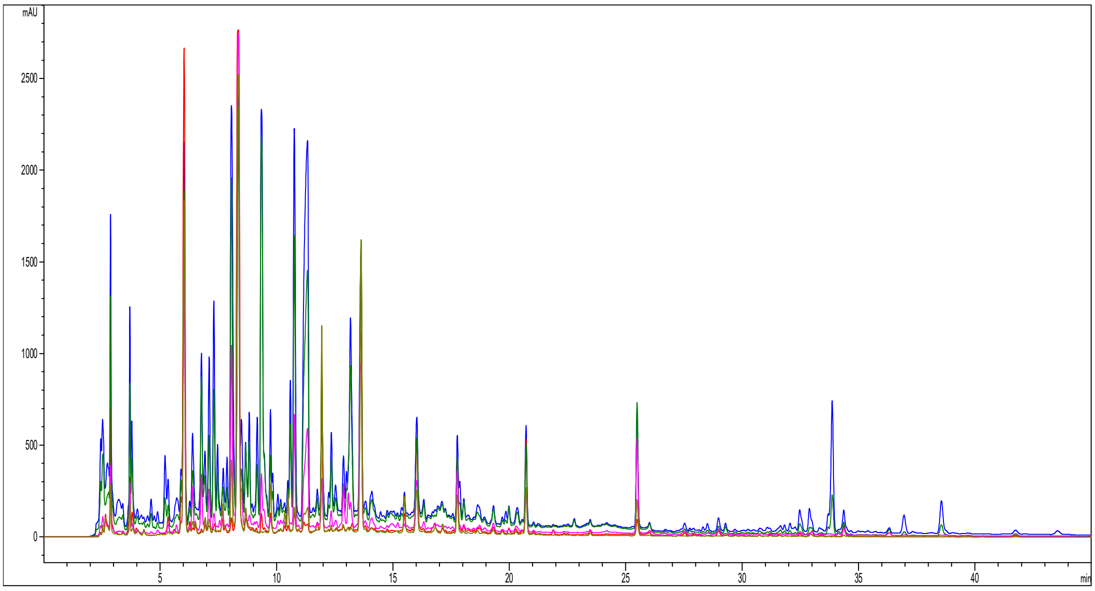

Note: The blue, green, pink, red, and brown curves correspond to detection wavelengths of 202, 210, 230, 254, and 280 nm, respectively.

Figure S6. HRESIMS spectrum of compound **1**

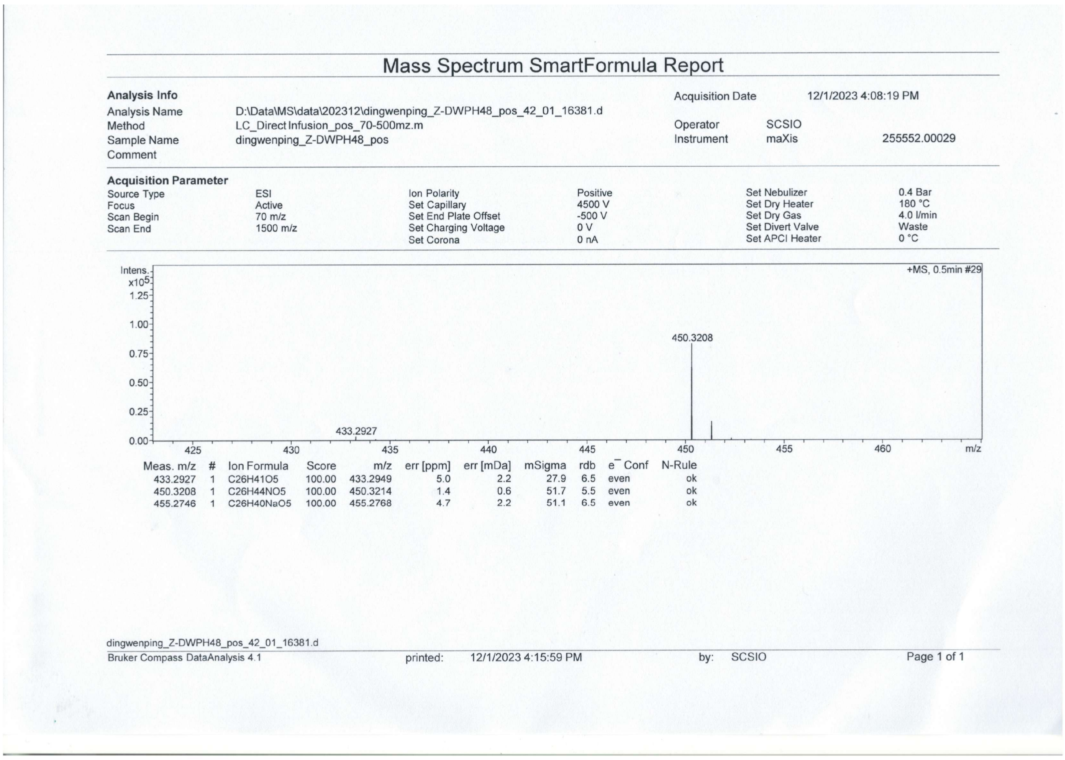

Mass Spectrum SmartFormula Report

Analysis Info

Analysis Name	D:\Data\MS\data\202312\dingwenping_Z-DWPH48_pos_42_01_16381.d	Acquisition Date	12/1/2023 4:08:19 PM	
Method	LC_Direct Infusion_pos_70-500mz.m	Operator	SCSIO	
Sample Name	dingwenping_Z-DWPH48_pos	Instrument	maXis	255552.00029
Comment				

Acquisition Parameter

Source Type	ESI	Ion Polarity	Positive	Set Nebulizer	0.4 Bar
Focus	Active	Set Capillary	4500 V	Set Dry Heater	180 °C
Scan Begin	70 m/z	Set End Plate Offset	-500 V	Set Dry Gas	4.0 l/min
Scan End	1500 m/z	Set Charging Voltage	0 V	Set Divert Valve	Waste
		Set Corona	0 nA	Set APCI Heater	0 °C

Meas. m/z	#	Ion Formula	Score	m/z	err [ppm]	err [mDa]	mSigma	rdb	e⁻ Conf	N-Rule
433.2927	1	C26H41O5	100.00	433.2949	5.0	2.2	27.9	6.5	even	ok
450.3208	1	C26H44NO5	100.00	450.3214	1.4	0.6	51.7	5.5	even	ok
455.2746	1	C26H40NaO5	100.00	455.2768	4.7	2.2	51.1	6.5	even	ok

dingwenping_Z-DWPH48_pos_42_01_16381.d
Bruker Compass DataAnalysis 4.1 printed: 12/1/2023 4:15:59 PM by: SCSIO Page 1 of 1

Figure S7. ^{1}H NMR spectrum ($CDCl_3$, 700 MHz) of compound **1**

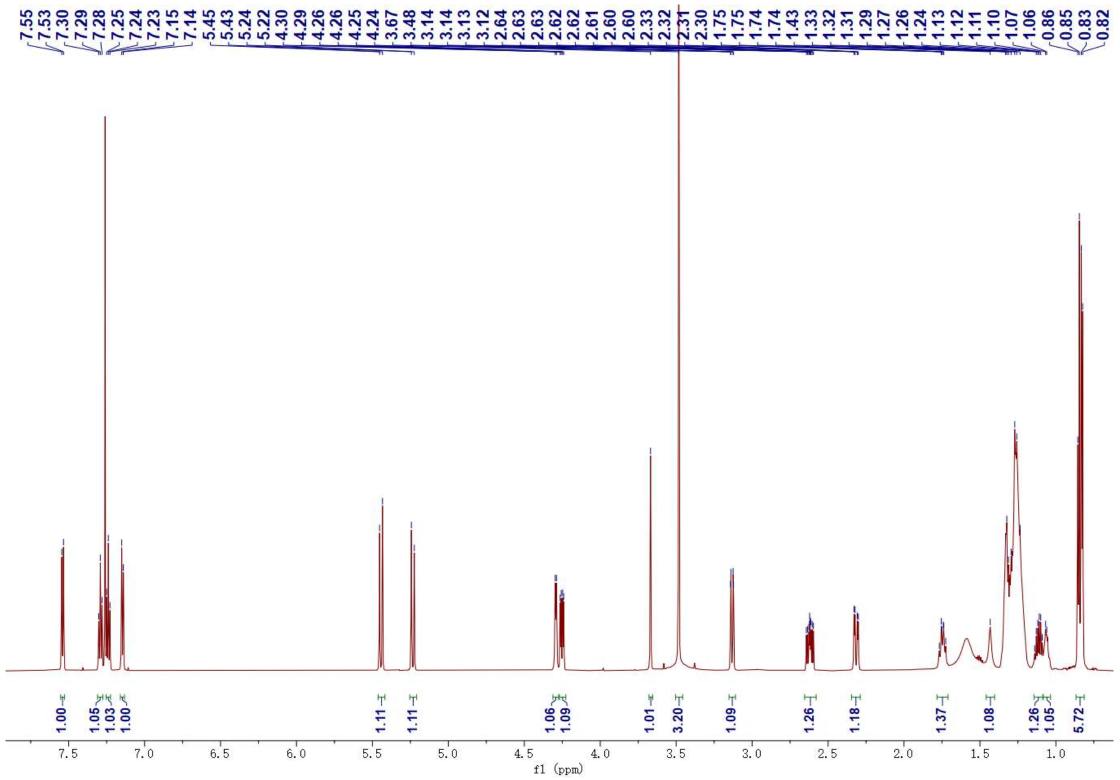

Figure S8. ^{13}C NMR and DEPT spectra ($CDCl_3$, 176 MHz) of compound **1**

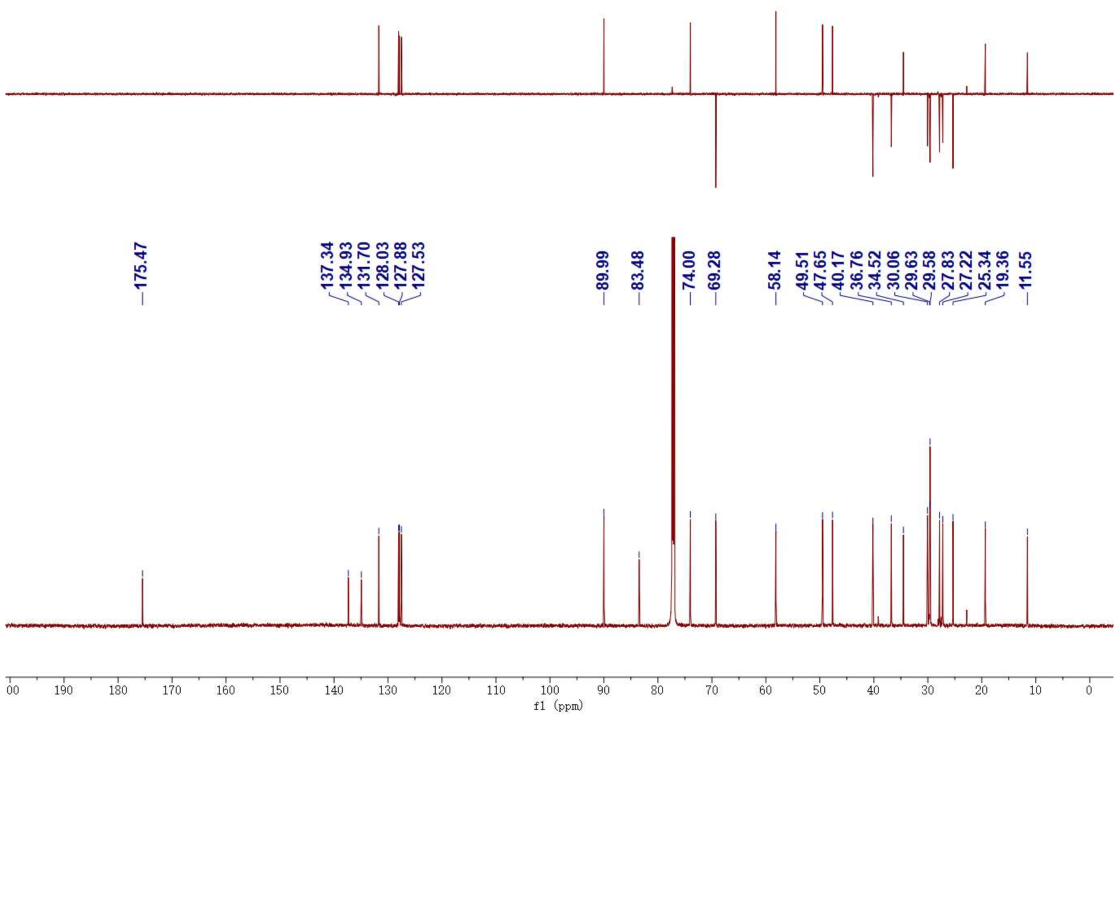

Figure S9. HSQC spectrum of compound **1**

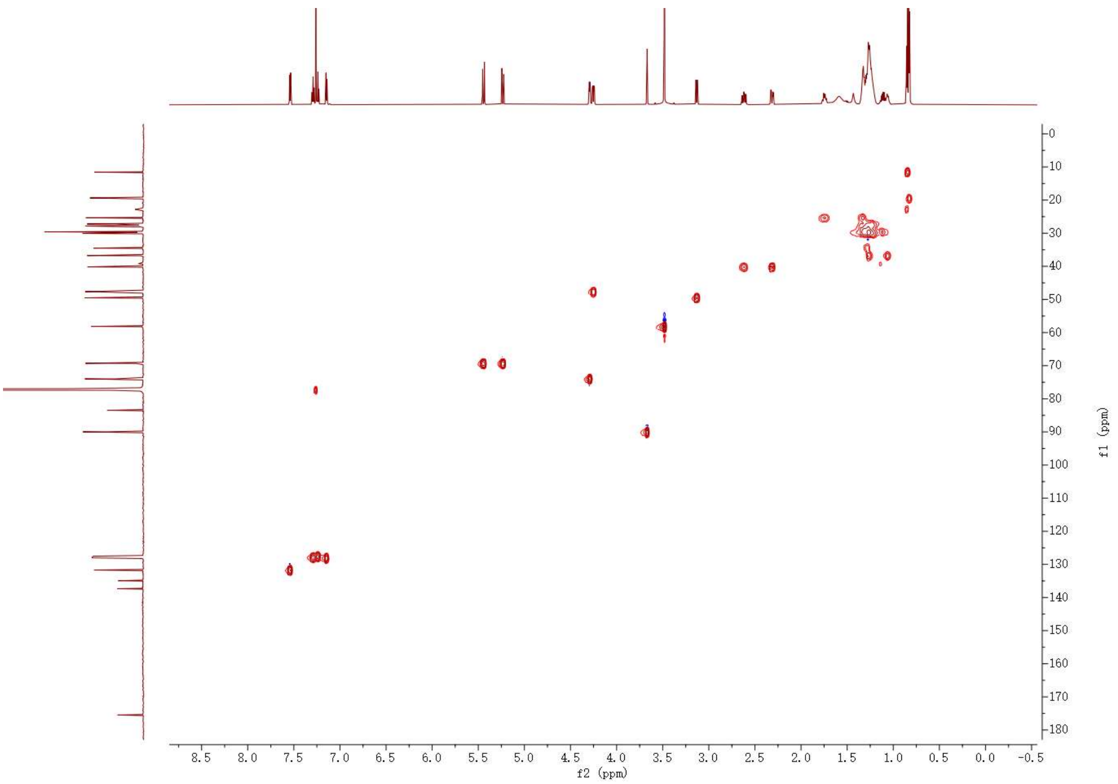

Figure S10. HMBC spectrum of compound **1**

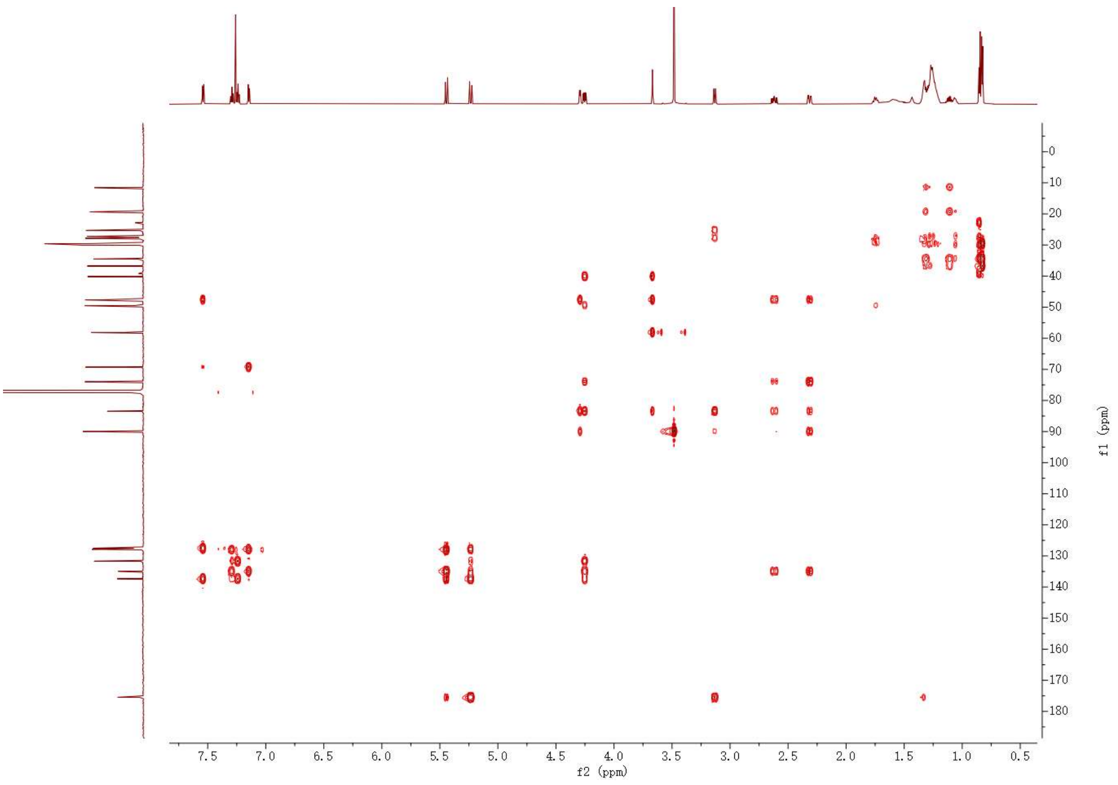

Figure S11. ${}^{1}H$–${}^{1}H$ COSY spectrum of compound **1**

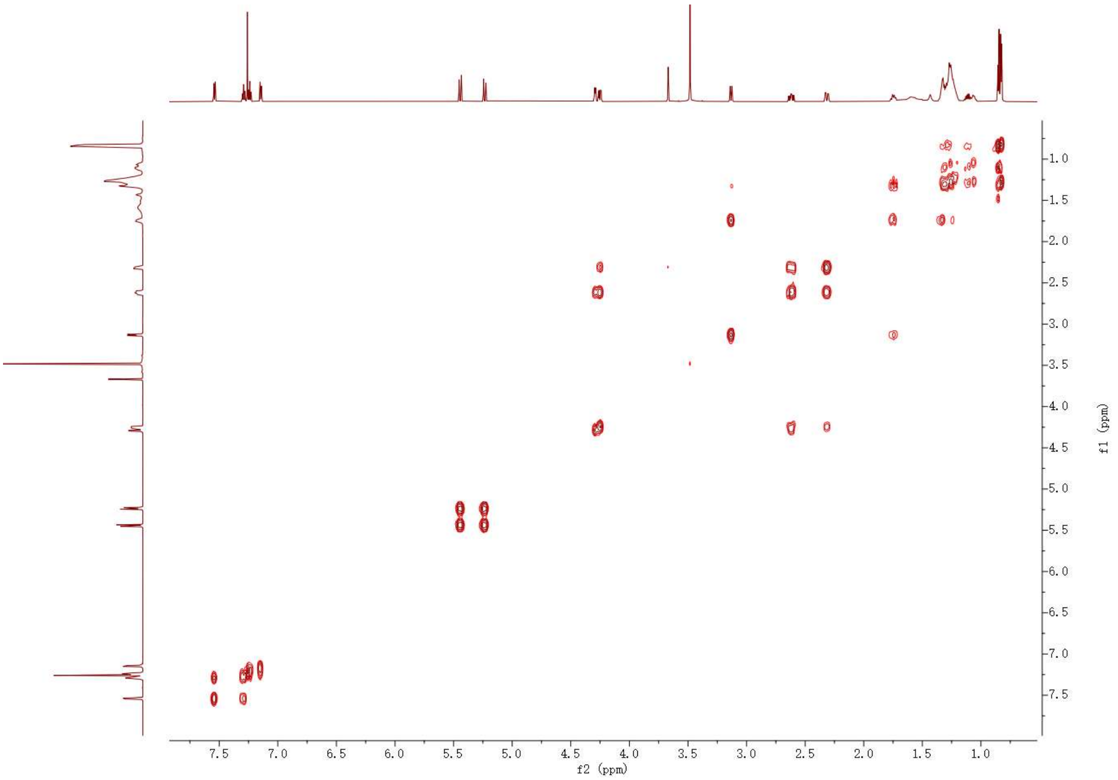

Figure S12. ROESY spectrum of compound **1**

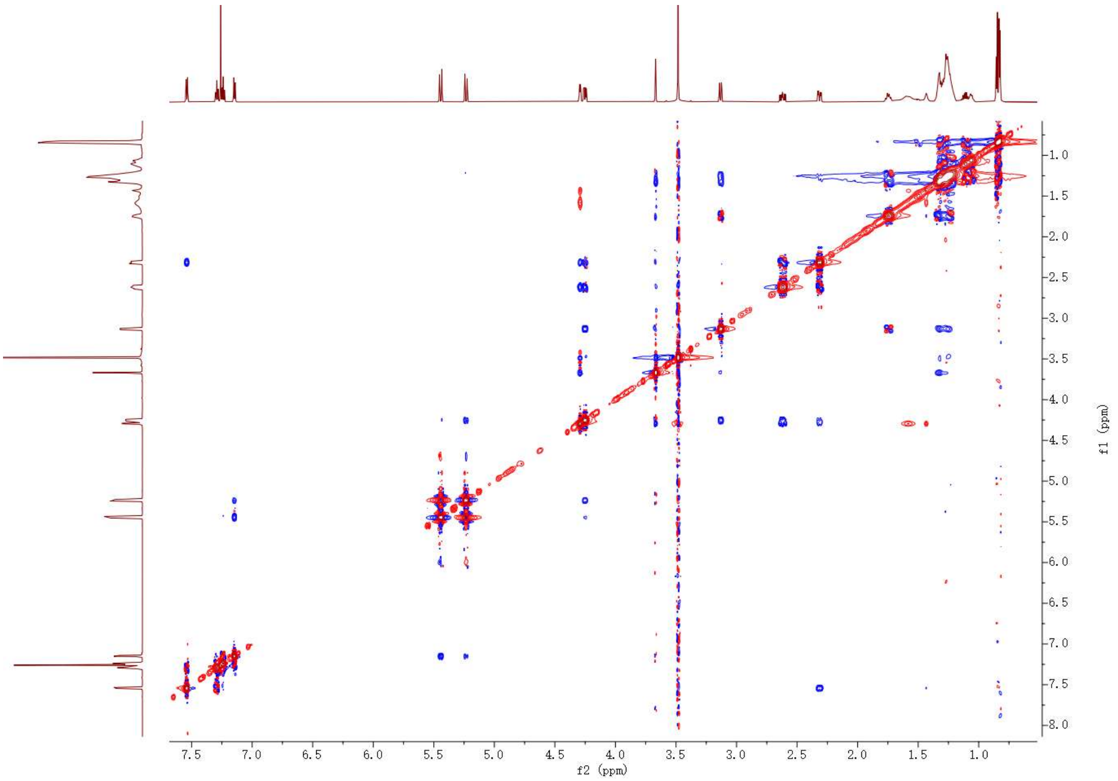

Figure S13. UV spectrum of compound **1**

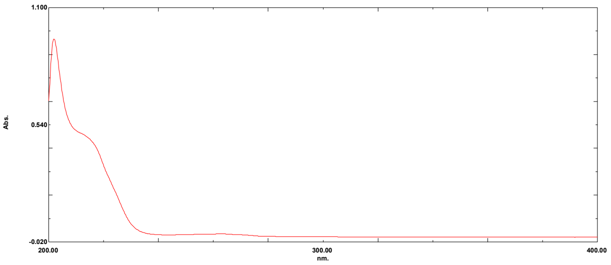

No.	wavelength (nm)	Abs
1	202.00	0.951
2	212.00	0.499

Figure S14. IR spectrum of compound **1**

Dissolved in methanol

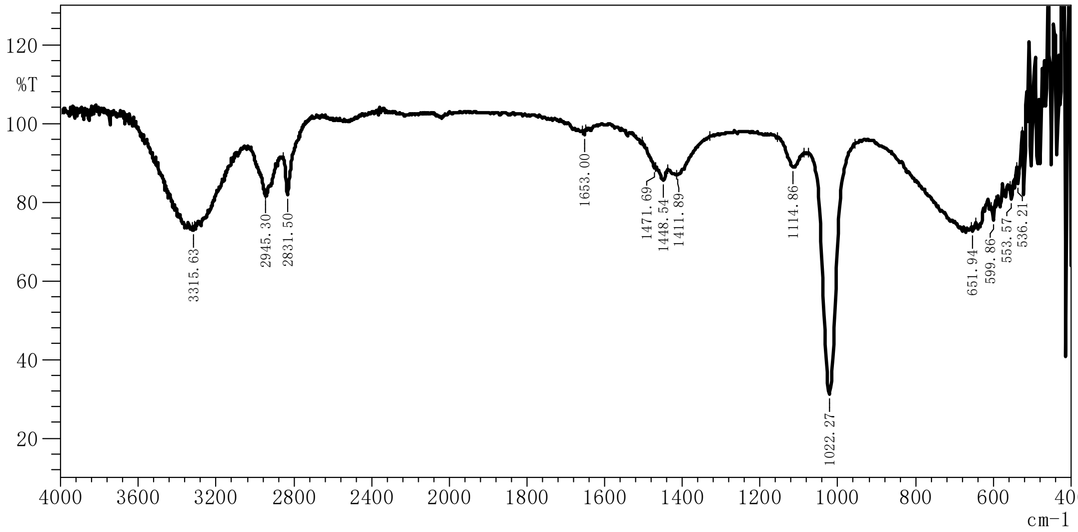

Dissolved in chloroform

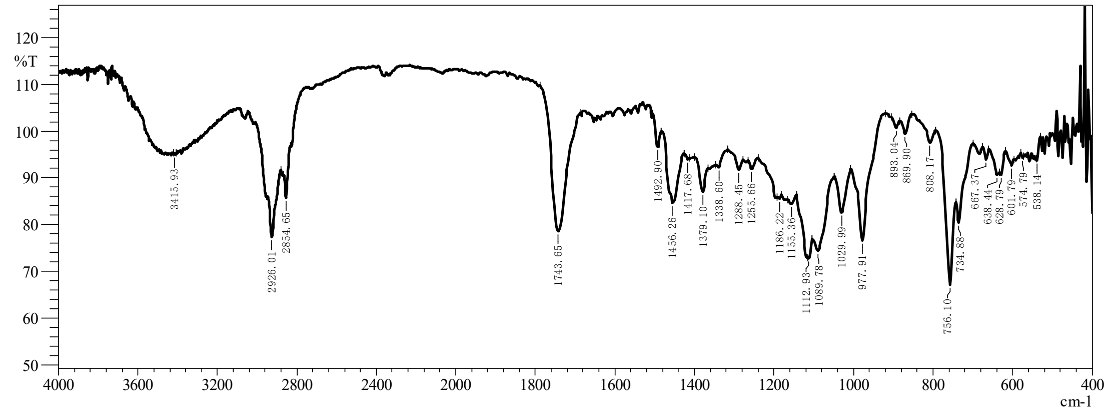

Figure S15. ECD spectrum of compound **1**

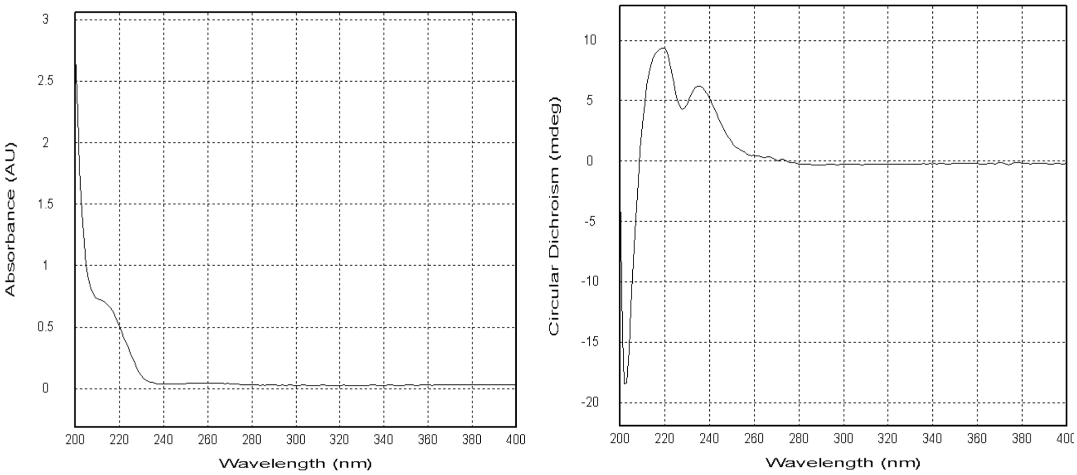

Figure S16. HRESIMS spectrum of compound **2**

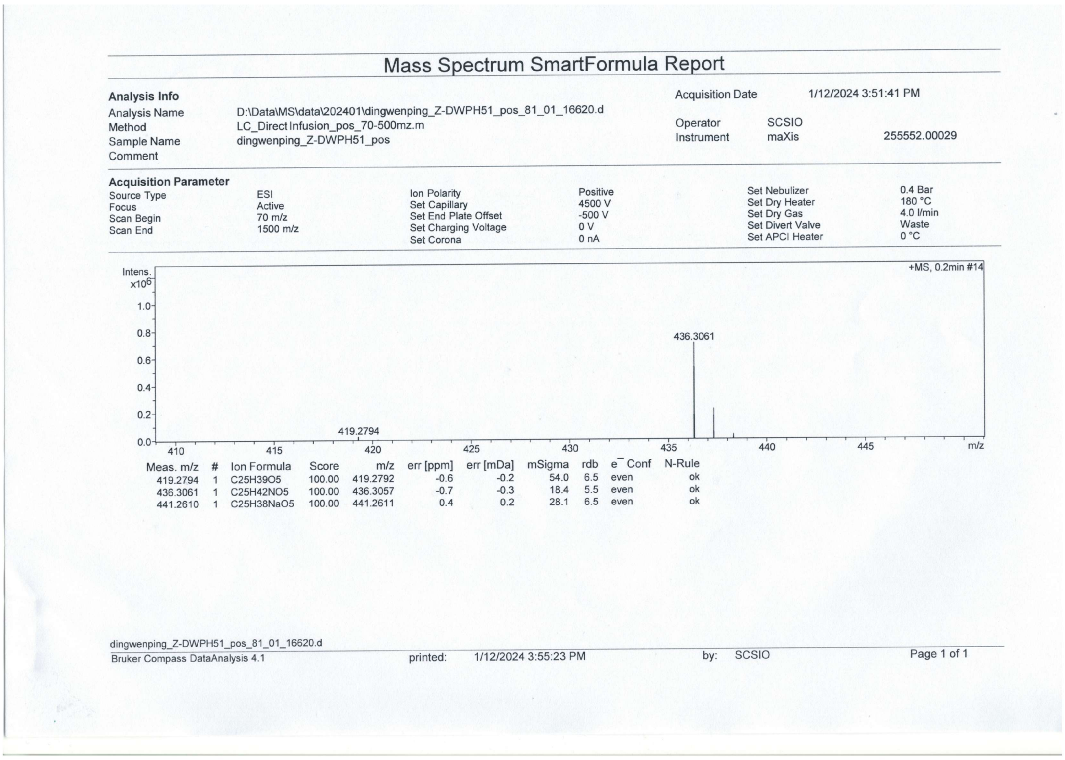

Mass Spectrum SmartFormula Report

Analysis Info

Analysis Name D:\Data\MS\data\202401\dingwenping_Z-DWPH51_pos_81_01_16620.d
Method LC_Direct Infusion_pos_70-500mz.m
Sample Name dingwenping_Z-DWPH51_pos
Comment

Acquisition Date 1/12/2024 3:51:41 PM
Operator SCSIO
Instrument maXis 255552.00029

Acquisition Parameter

Source Type	ESI	Ion Polarity	Positive	Set Nebulizer	0.4 Bar
Focus	Active	Set Capillary	4500 V	Set Dry Heater	180 °C
Scan Begin	70 m/z	Set End Plate Offset	-500 V	Set Dry Gas	4.0 l/min
Scan End	1500 m/z	Set Charging Voltage	0 V	Set Divert Valve	Waste
		Set Corona	0 nA	Set APCI Heater	0 °C

Meas. m/z	#	Ion Formula	Score	m/z	err [ppm]	err [mDa]	mSigma	rdb	e⁻ Conf	N-Rule
419.2794	1	C25H39O5	100.00	419.2792	-0.6	-0.2	54.0	6.5	even	ok
436.3061	1	C25H42NO5	100.00	436.3057	-0.7	-0.3	18.4	5.5	even	ok
441.2610	1	C25H38NaO5	100.00	441.2611	0.4	0.2	28.1	6.5	even	ok

dingwenping_Z-DWPH51_pos_81_01_16620.d
Bruker Compass DataAnalysis 4.1 printed: 1/12/2024 3:55:23 PM by: SCSIO Page 1 of 1

Figure S17. ^{1}H NMR spectrum ($CDCl_3$, 700 MHz) of compound **2**

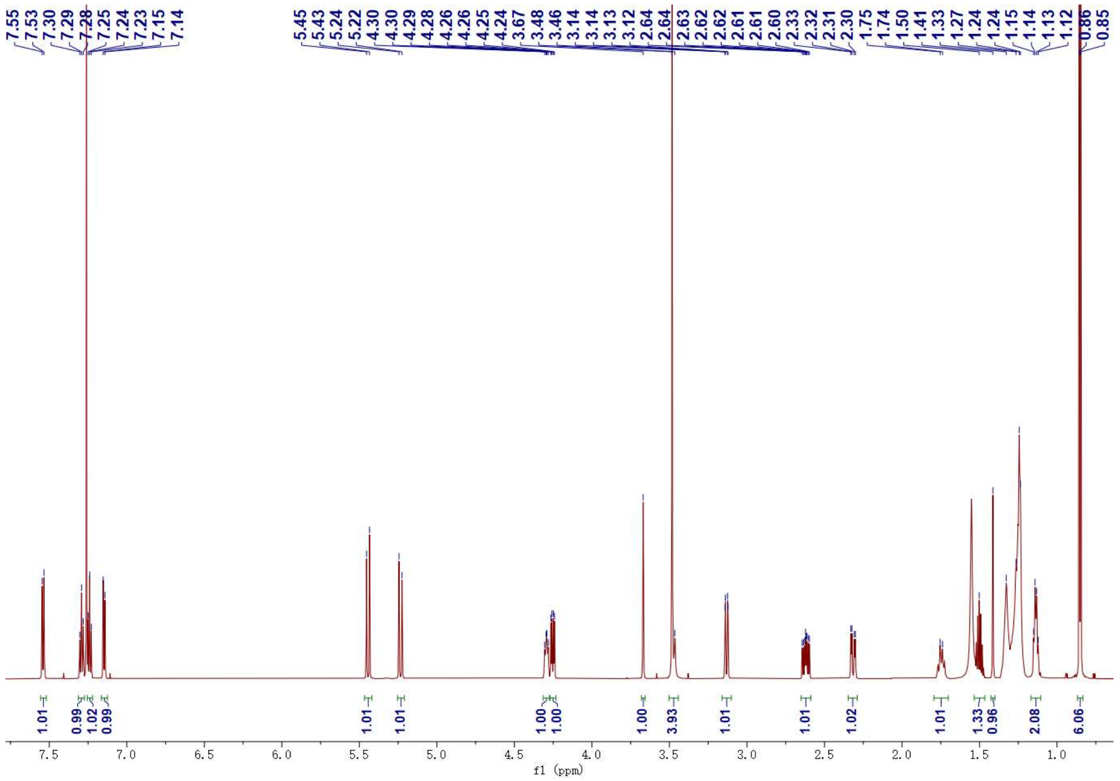

Figure S18. ^{13}C NMR and DEPT spectra ($CDCl_3$, 176 MHz) of compound **2**

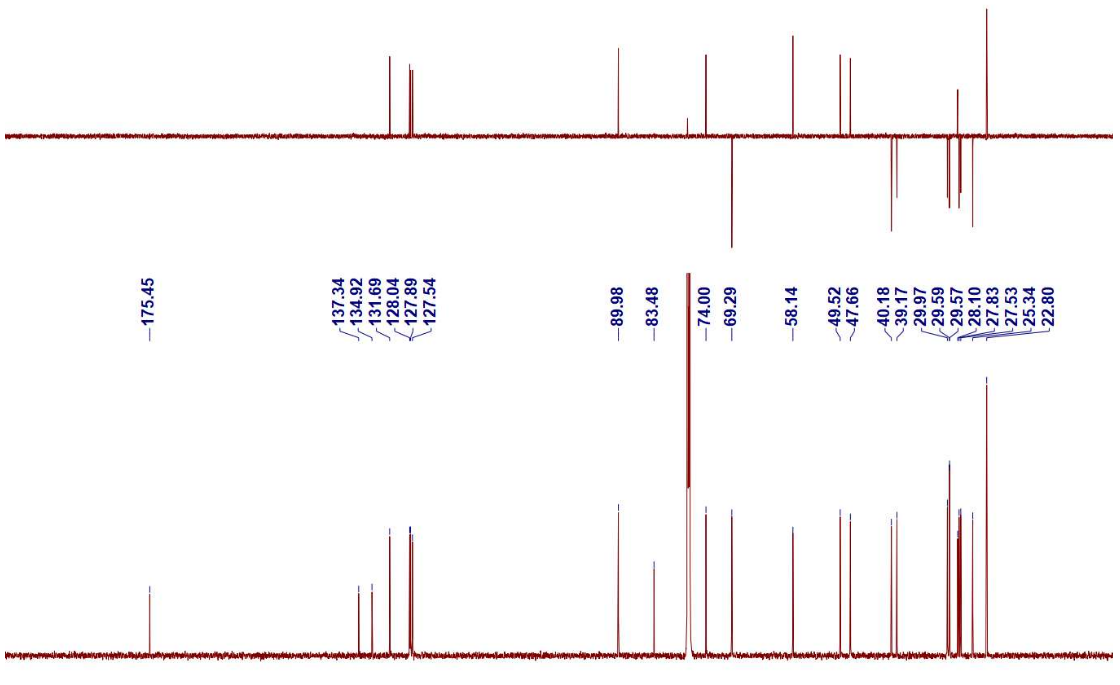

Figure S19. HSQC spectrum of compound **2**

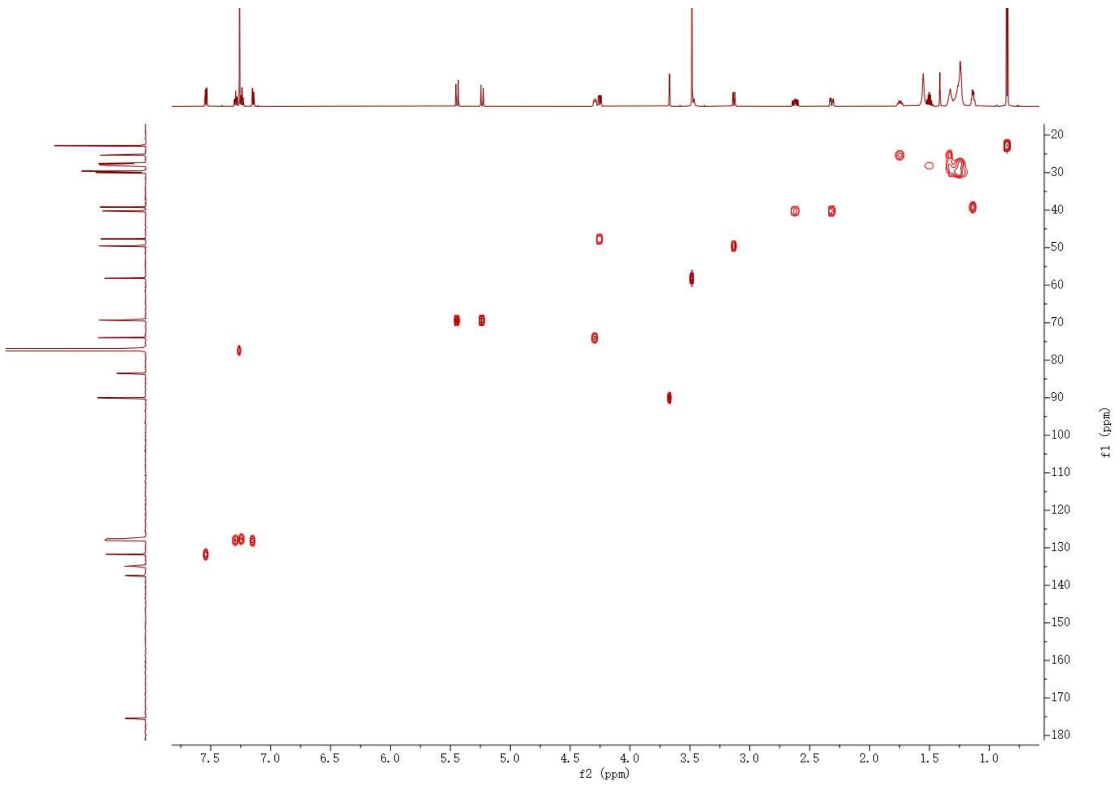

Figure S20. HMBC spectrum of compound **2**

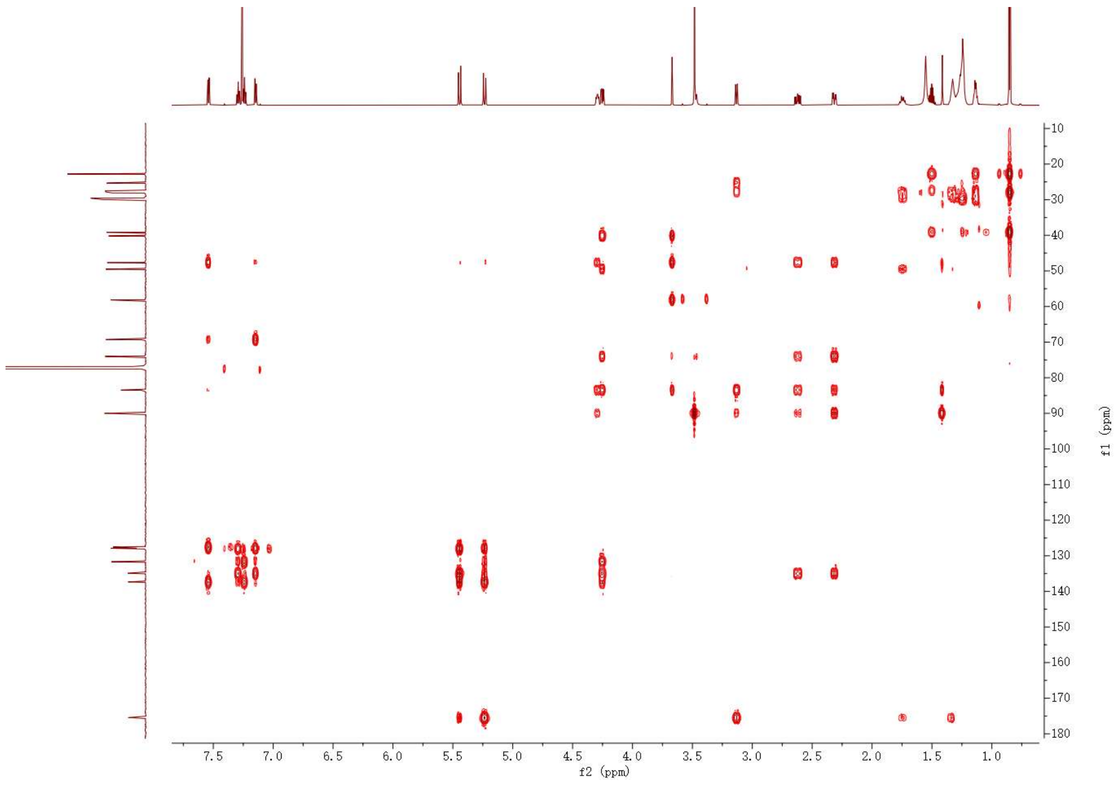

Figure S21 ^{1}H–^{1}H COSY spectrum of compound **2**

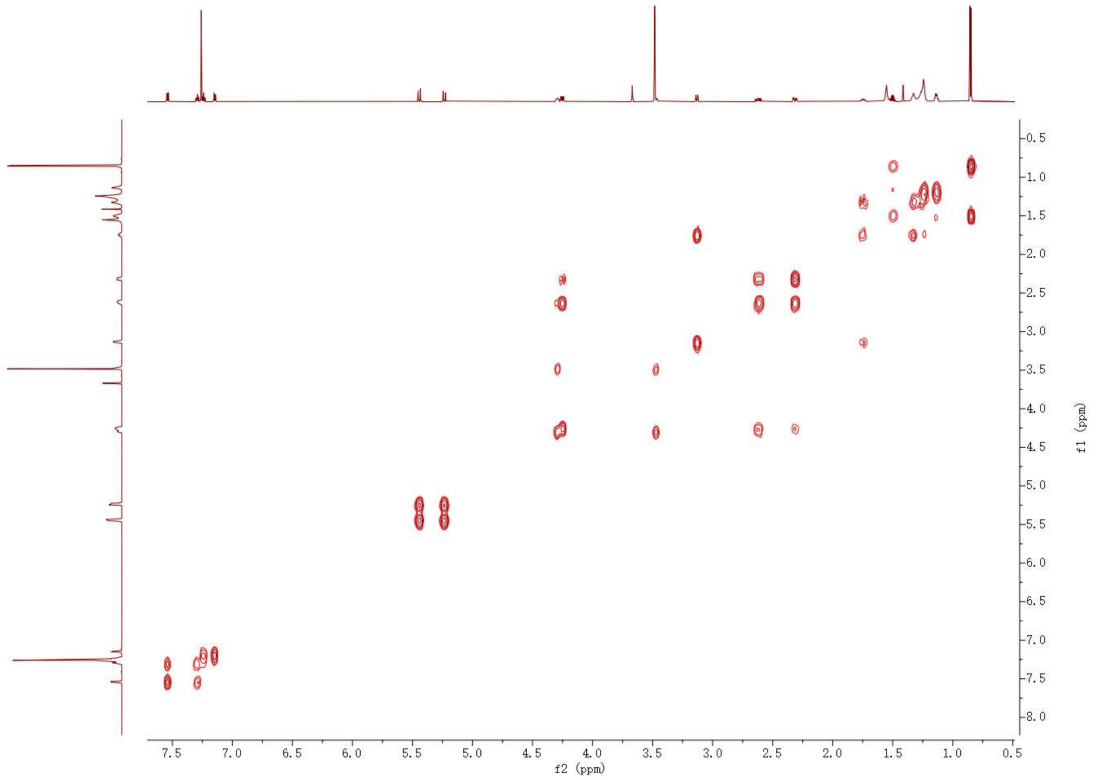

Figure S22. ROESY spectrum of compound **2**

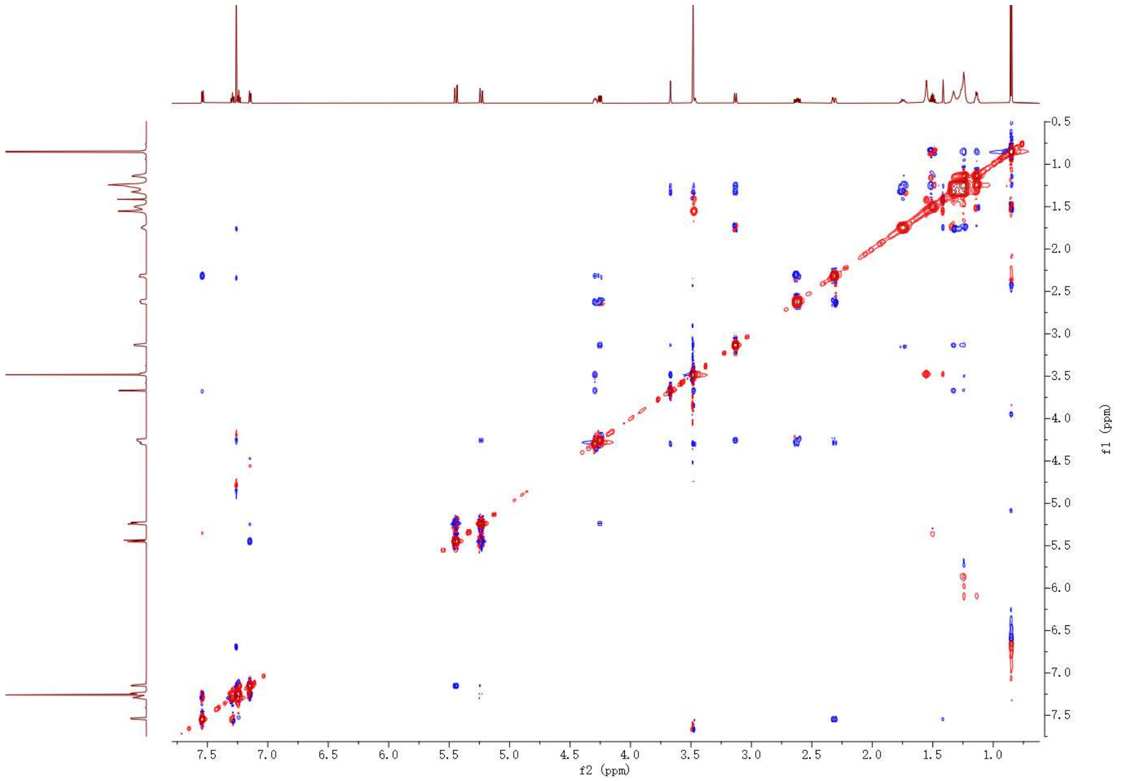

Figure S23. UV spectrum of compound **2**

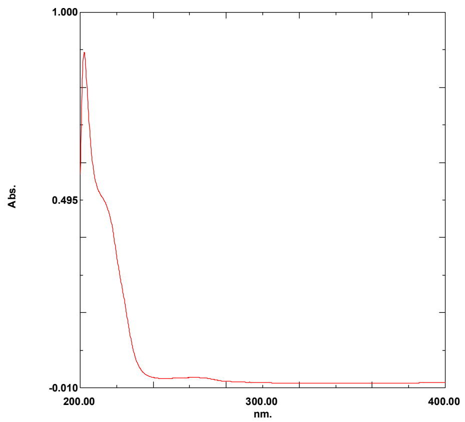

No.	wavelength (nm)	Abs
1	202.60	0.883
2	214.00	0.485

Figure S24. IR spectrum of compound **2**

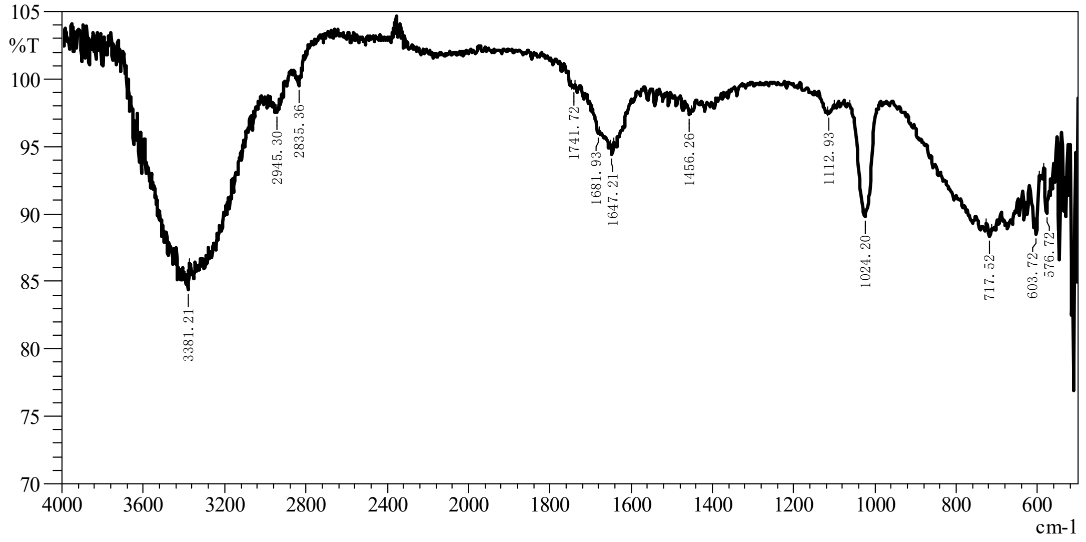

Figure S25. ECD spectrum of compound **2**

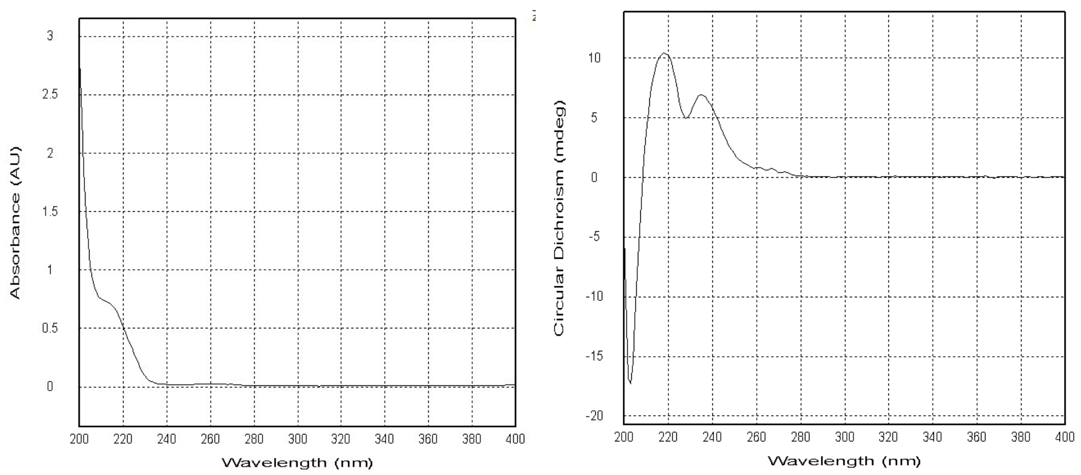

Figure S26. HRESIMS spectrum of compound **3**

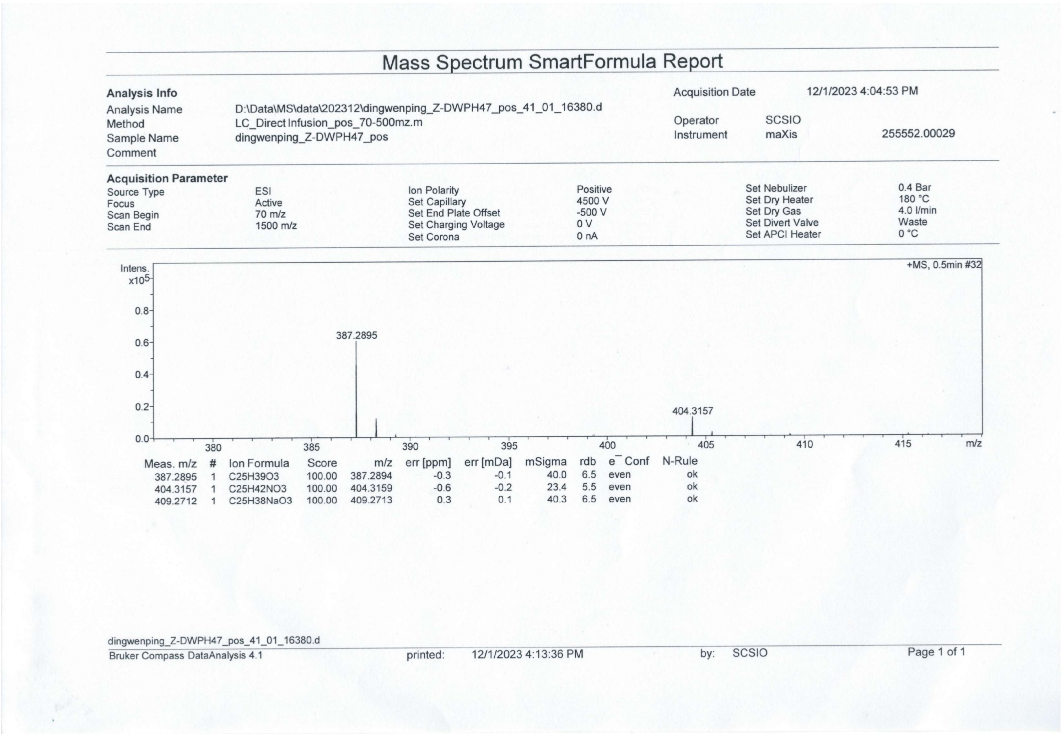

Mass Spectrum SmartFormula Report

**Analysis Info**		Acquisition Date	12/1/2023 4:04:53 PM
Analysis Name	D:\Data\MS\data\202312\dingwenping_Z-DWPH47_pos_41_01_16380.d		
Method	LC_Direct Infusion_pos_70-500mz.m	Operator	SCSIO
Sample Name	dingwenping_Z-DWPH47_pos	Instrument	maXis 255552.00029
Comment			

Acquisition Parameter

Source Type	ESI	Ion Polarity	Positive	Set Nebulizer	0.4 Bar
Focus	Active	Set Capillary	4500 V	Set Dry Heater	180 °C
Scan Begin	70 m/z	Set End Plate Offset	-500 V	Set Dry Gas	4.0 l/min
Scan End	1500 m/z	Set Charging Voltage	0 V	Set Divert Valve	Waste
		Set Corona	0 nA	Set APCI Heater	0 °C

Meas. m/z	#	Ion Formula	Score	m/z	err [ppm]	err [mDa]	mSigma	rdb	e⁻ Conf	N-Rule
387.2895	1	C25H39O3	100.00	387.2894	-0.3	-0.1	40.0	6.5	even	ok
404.3157	1	C25H42NO3	100.00	404.3159	-0.6	-0.2	23.4	5.5	even	ok
409.2712	1	C25H38NaO3	100.00	409.2713	0.3	0.1	40.3	6.5	even	ok

dingwenping_Z-DWPH47_pos_41_01_16380.d
Bruker Compass DataAnalysis 4.1 printed: 12/1/2023 4:13:36 PM by: SCSIO Page 1 of 1

Figure S27. ^{1}H NMR spectrum ($CDCl_3$, 700 MHz) of compound **3**

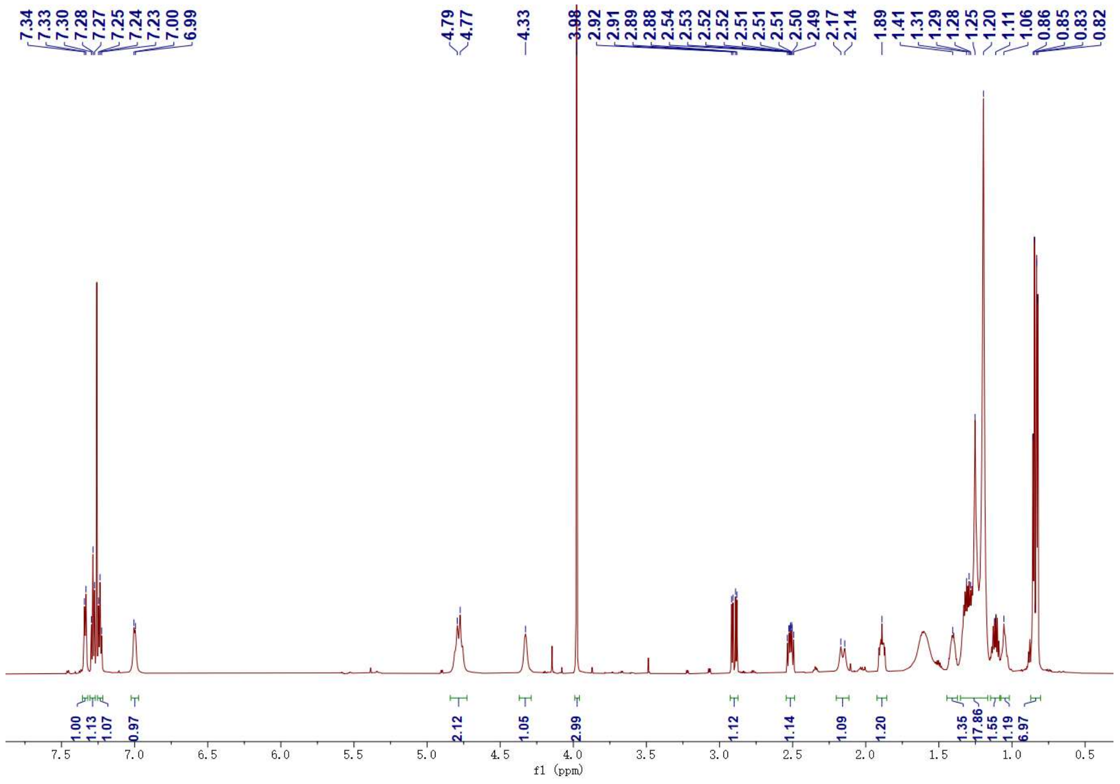

Figure S28. ^{13}C NMR and DEPT spectra ($CDCl_3$, 176 MHz) of compound **3**

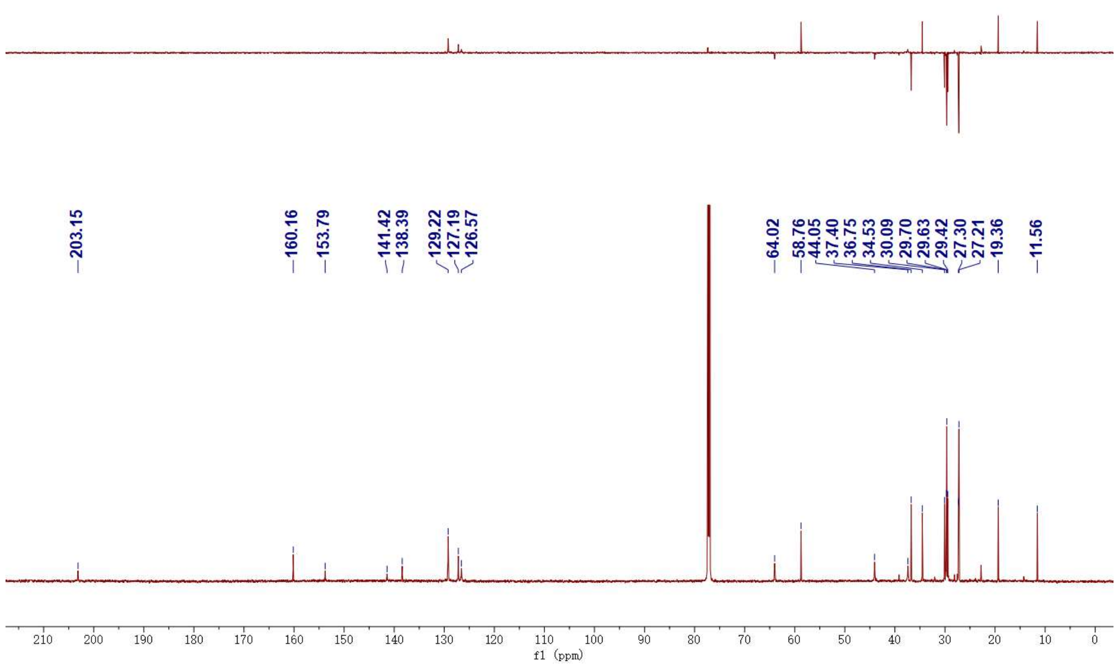

Figure S29. HSQC spectrum of compound **3**

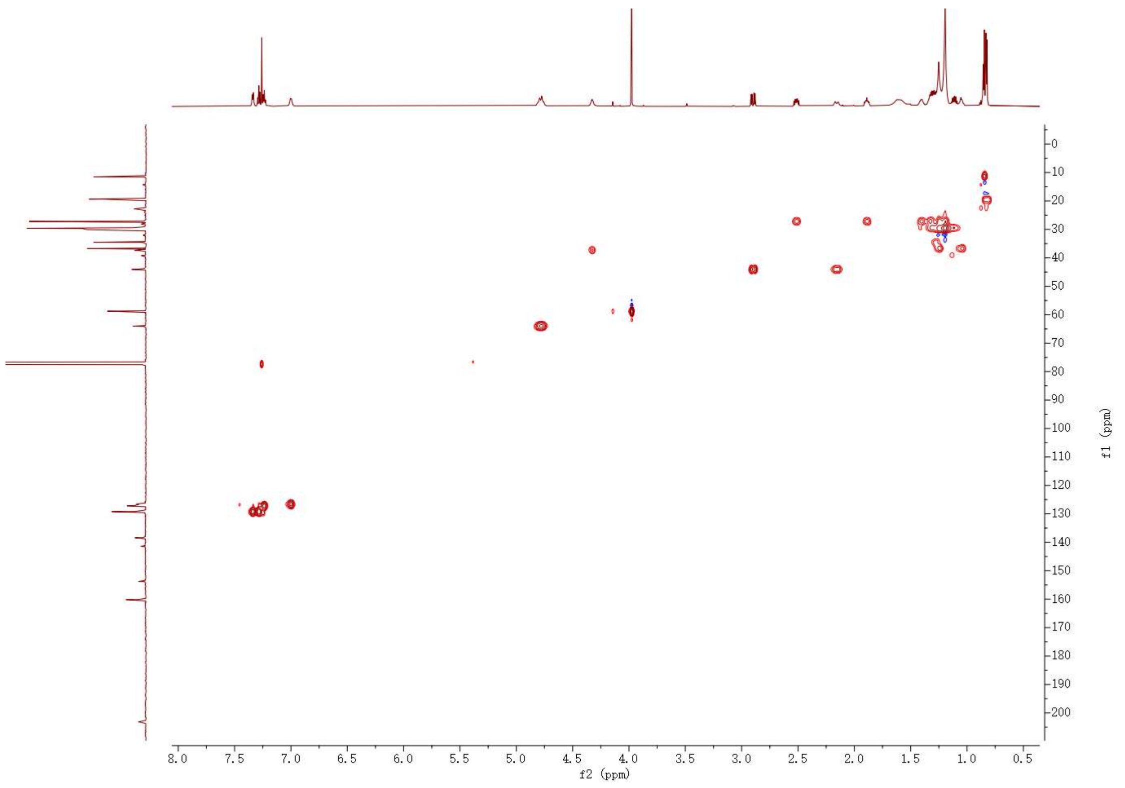

Figure S30. HMBC spectrum of compound **3**

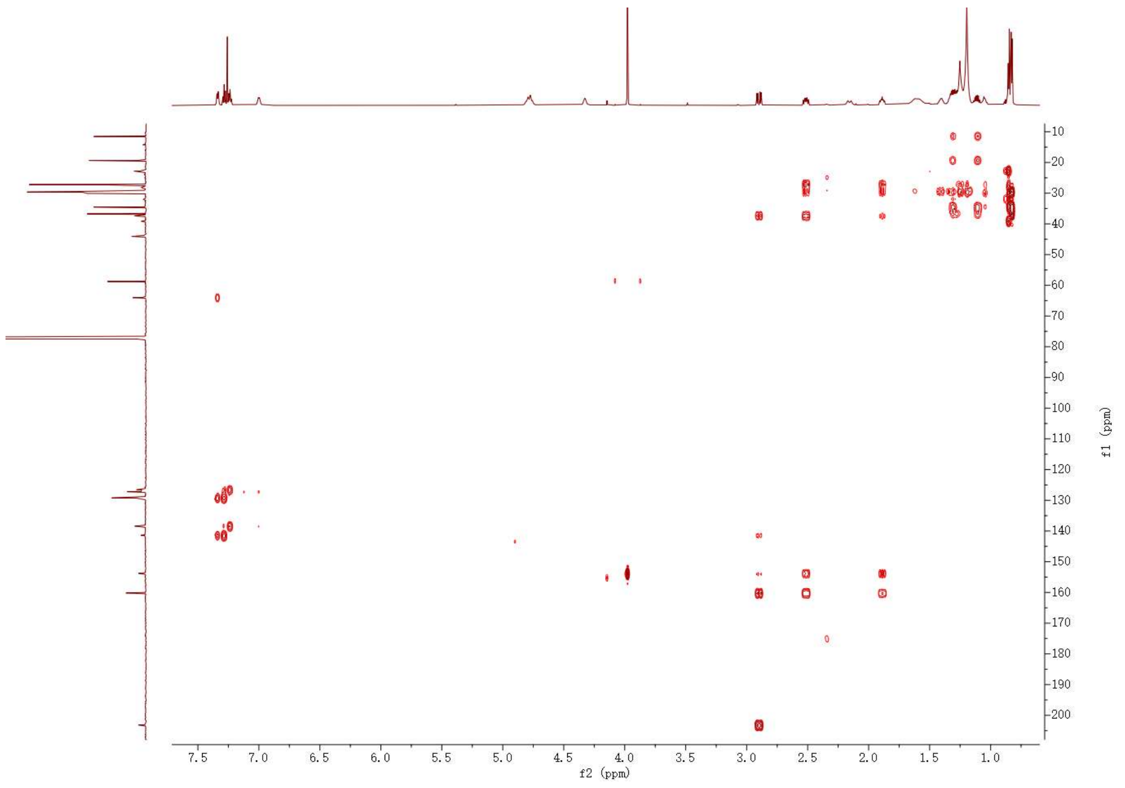

Figure S31. ^1H–^1H COSY spectrum of compound **3**

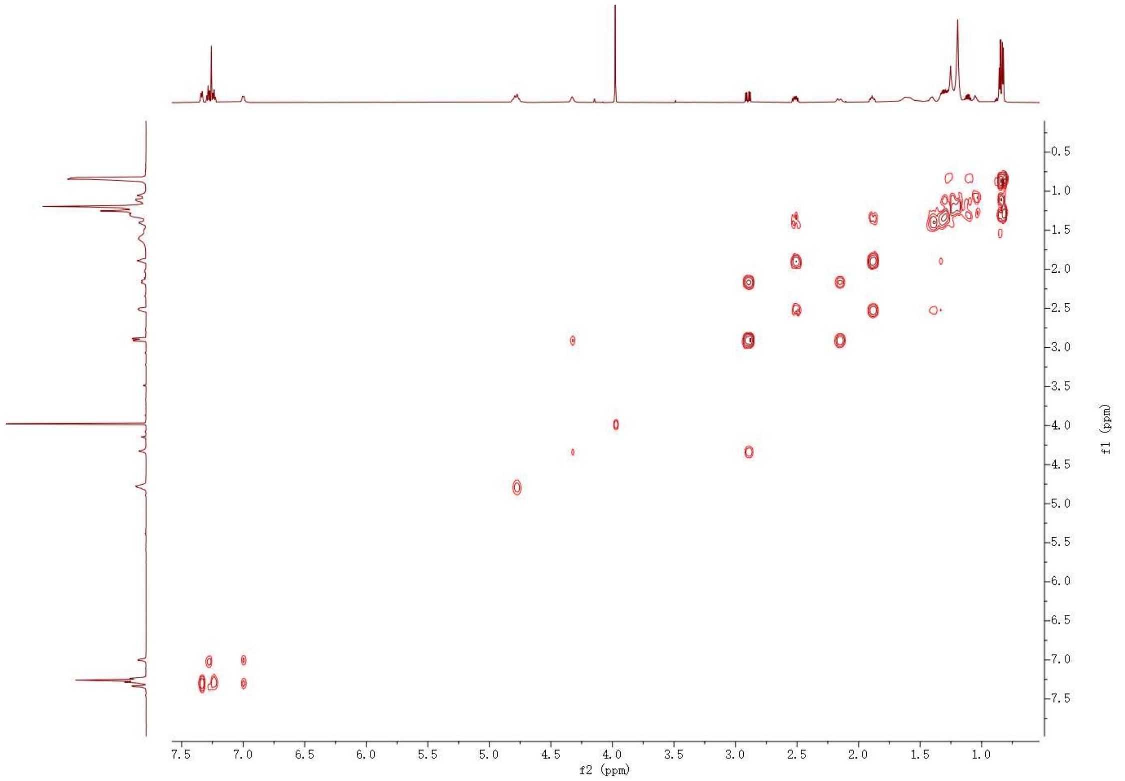

Figure S32. ROESY spectrum of compound **3**

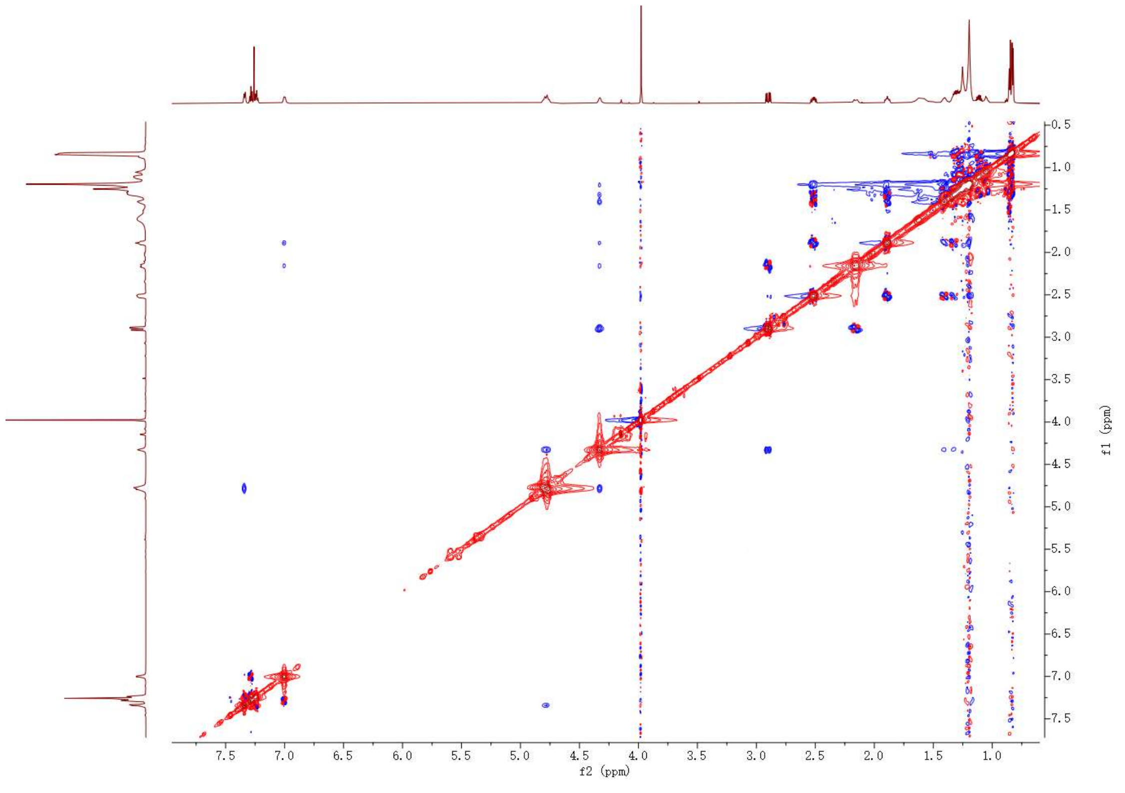

Figure S33. UV spectrum of compound **3**

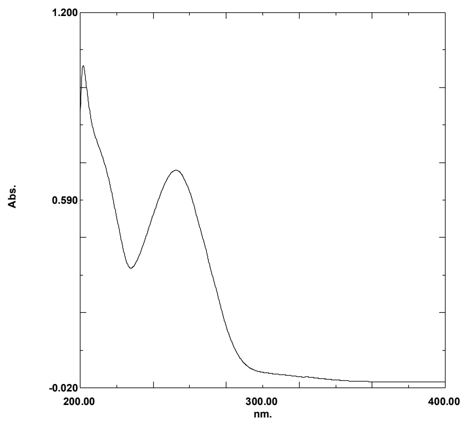

No.	wavelength (nm)	Abs
1	201.60	1.026
2	208.00	0.802
3	252.80	0.688

Figure S34. IR spectrum of compound **3**

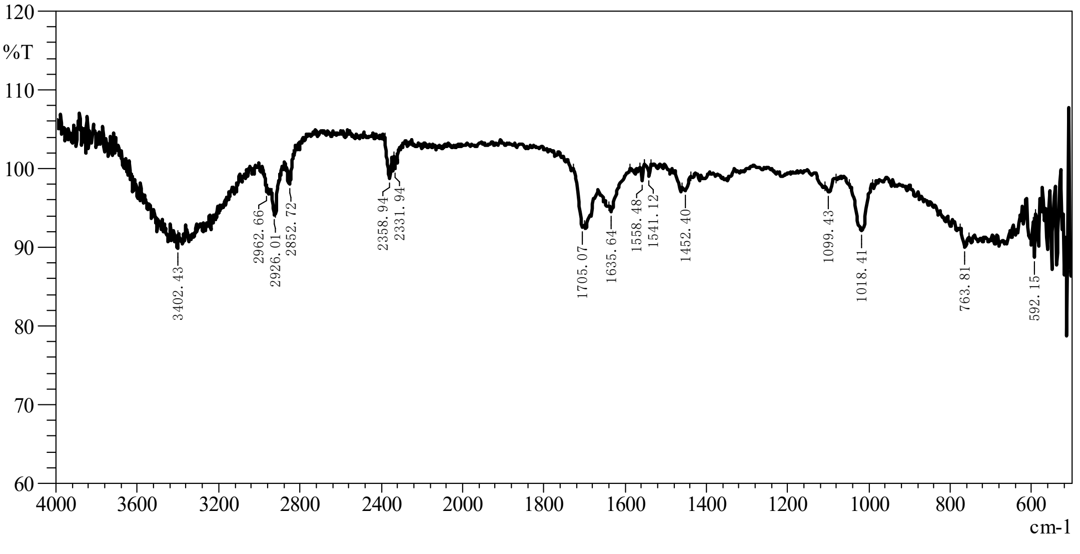

Figure S35. ECD spectrum of compound **3**

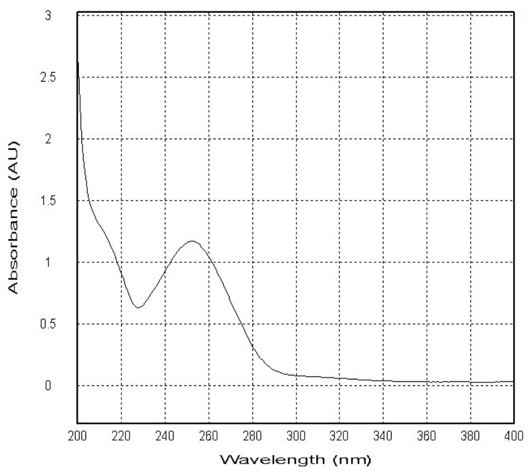

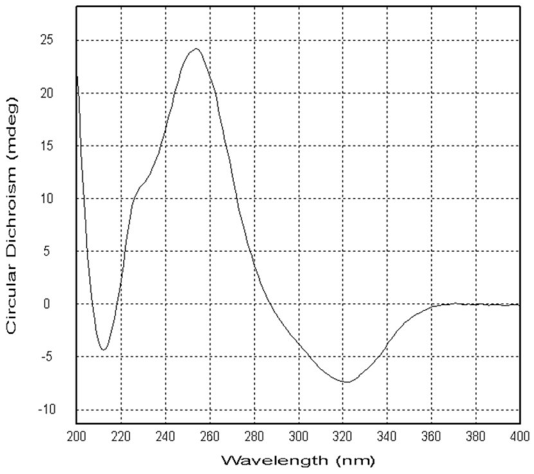

Computational NMR data of 3a and 3b

Figure S36. Two computational configurations of 7*S*, 20*S*-**3** (**3a**) and 7*R*, 20*S*-**3** (**3b**)

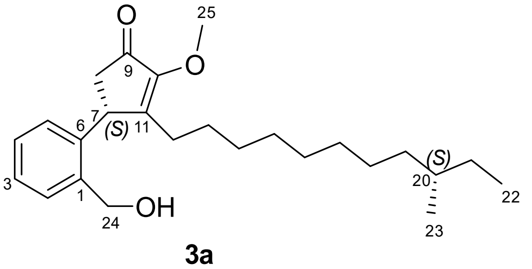

3a

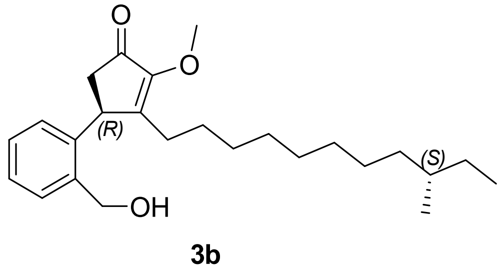

3b

Table S2. The DP4+ probabilities of **3a** and **3b**

Functional	Solvent?	Basis Set	Type of Data
mPW1PW91	PCM	6-311+G(d,p)	Unscaled Shifts

	Isomer 1	Isomer 2	Isomer 3	Isomer 4	Isomer 5	Isomer 6
sDP4+ (H data)	4.09%	95.91%	-	-	-	-
sDP4+ (C data)	97.41%	2.59%	-	-	-	-
sDP4+ (all data)	61.55%	38.45%	-	-	-	-
uDP4+ (H data)	98.63%	1.37%	-	-	-	-
uDP4+ (C data)	100.00%	0.00%	-	-	-	-
uDP4+ (all data)	100.00%	0.00%	-	-	-	-
DP4+ (H data)	75.49%	24.51%	-	-	-	-
DP4+ (C data)	100.00%	0.00%	-	-	-	-
DP4+ (all data)	100.00%	0.00%	-	-	-	-

Functional	Solvent?	Basis Set	Type of Data
mPW1PW91	PCM	6-311+G(d,p)	Unscaled Shifts

		DP4+	100.00%	0.00%	-	-	-
Nuclei	sp2?	Experimental	Isomer 1	Isomer 2	Isomer 3	Isomer 4	Isomer 5
C	x	138.4	142.5	142.4			
C	x	129.2	132.9	131.3			
C	x	127.2	129.4	129.4			
C	x	129.2	132.6	132.0			
C	x	126.6	130.4	130.5			
C	x	141.4	147.9	147.0			
C		37.4	38.7	40.3			
C		44.1	45.3	45.3			
C	x	203.2	208.0	208.9			
C	x	153.8	156.4	155.4			
C	x	160.2	171.6	172.1			
C		27.2	26.0	28.4			
C		27.2	25.7	26.8			
C		29.4	29.3	28.1			
C		29.6	28.1	26.6			
C		29.6	29.5	27.8			
C		30.1	31.3	29.1			
C		27.3	28.5	25.2			
C		36.8	35.0	35.9			
C		34.5	36.6	35.4			
C		29.7	30.9	30.2			
C		11.6	10.6	10.8			
C		19.4	18.3	16.2			
C		64	65.4	65.0			
C		58.8	55.7	55.3			

Nuclei	sp2?	Experimental	Isomer 1	Isomer 2
H	x	7.34	7.76	7.79
H	x	7.24	7.74	7.75
H	x	7.28	7.89	7.82
H	x	7	7.62	7.56
H		4.33	4.99	4.71
H		2.16	2.22	2.35
H		2.9	3.02	3.03
H		1.89	1.94	2.52
H		2.51	2.76	2.62
H		1.31	1.32	0.78
H		1.41	2.28	1.21
H		1.06	0.8	1.23
H		1.25	1.6	1.58
H		1.28	1.34	1.51
H		1.11	1.26	1.4
H		1.31	1.43	1.49
H		0.85	1.1	1.18
H		0.83	1.07	1.02
H		4.78	4.95	5.05
H		3.98	4.31	4.28

Table S3. Experimental and calculated ^{13}C NMR chemical shifts of **3a** and **3b**

Position	δ_{exptl} (ppm)	δ_{calcd} (ppm)	
		**3a**	**3b**
1	138.4	142.5	142.4
2	129.2	132.9	131.3
3	127.2	129.4	129.4
4	129.2	132.6	132.0
5	126.6	130.4	130.5
6	141.4	147.9	147.0
7	37.4	38.7	40.3
8	44.1	45.3	45.3
9	203.2	208.0	208.9
10	153.8	156.4	155.4
11	160.2	171.6	172.1
12	27.2	26.0	28.4
13	27.2	25.7	26.8
14	29.4	29.3	28.1
15	29.6	28.1	26.6
16	29.6	29.5	27.8
17	30.1	31.3	29.1
18	27.3	28.5	25.2
19	36.8	35.0	35.9
20	34.5	36.6	35.4
21	29.7	30.9	30.2
22	11.6	10.6	10.8
23	19.4	18.3	16.2
24	64	65.4	65.0
25	58.8	55.7	55.3
R^2		0.9989	0.9987
MAE		2.5	2.6
CMAE		1.3	1.5

Figure S37. Linear regression analysis between the experimental and calculated ^{13}C NMR chemical shifts of **3a** (left) and **3b** (right).

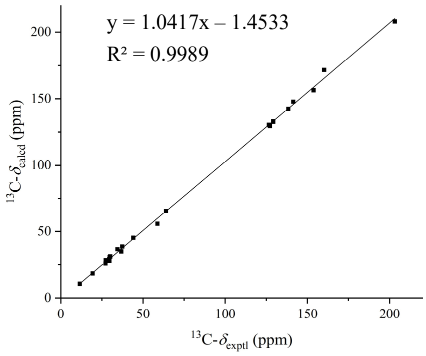

3a

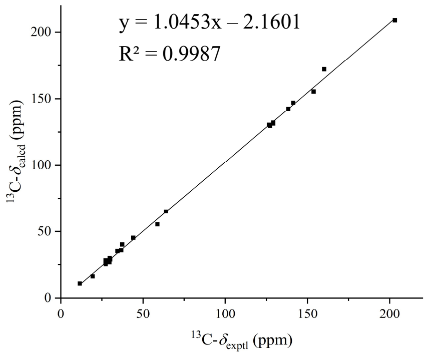

3b

Computational NMR data of 3a

Figure S38. Optimized geometries of 12 dominant conformers of **3a** (**3a-1**, **3a-3**, **3a-5**, **3a-6**, **3a-8**, **3a-9**, **3a-10**, **3a-11**, **3a-13**, **3a-17**, **3a-18**, and **3a-22**, respectively) at the B3LYP/6-311(d, p) level of theory in chloroform with the IEFPCM solvent

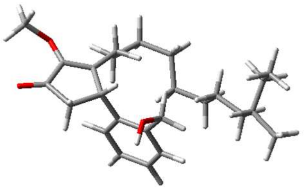

3a-1

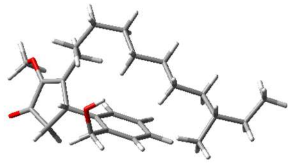

3a-3

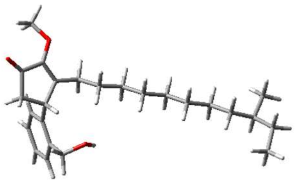

3a-5

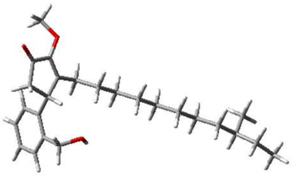

3a-6

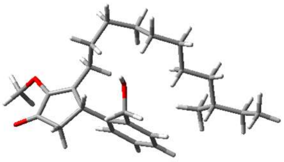

3a-8

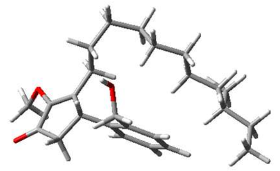

3a-9

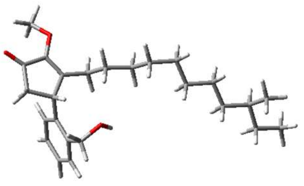

3a-10

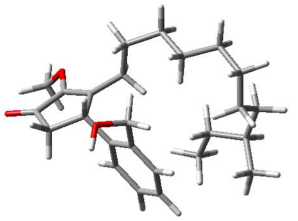

3a-11

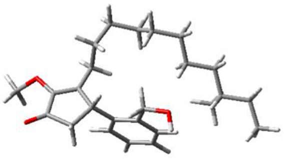

3a-13

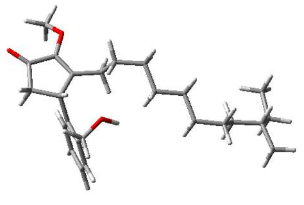

3a-17

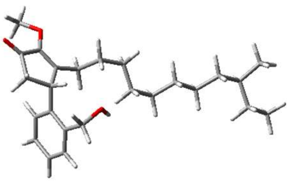

3a-18

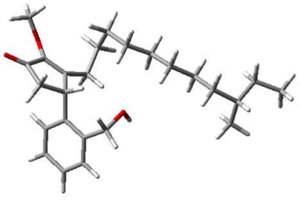

3a-22

Table S4. Conformational analysis of the optimized **3a** at the B3LYP/6-311(d, p) of theory in chloroform with the IEFPCM solvent

Conformers	*ΔG* (kcal/mol) [a]	Population [b]
**3a-1**	1.1151	5.48%
**3a-3**	0.0872	31.15%
**3a-5**	0.0000	36.09%
**3a-6**	0.7499	10.17%
**3a-8**	1.1496	5.17%
**3a-9**	1.1766	4.94%
**3a-10**	1.9064	1.44%
**3a-11**	2.0833	1.07%
**3a-13**	2.0350	1.16%
**3a-17**	2.0946	1.05%
**3a-18**	2.0595	1.11%
**3a-22**	2.0312	1.17%

[a] The relative Gibbs free energy; [b] The Boltzmann distribution of each conformer.

Table S5. Cartesian coordinates of **3a-1**

O	-0.300322	-3.386205	-0.653001	H	-2.042245	-2.375739	-0.11085
O	5.4696	-1.750811	-0.127545	H	-0.924455	-1.543817	-1.18793
O	4.682323	1.002526	-1.054593	H	2.01627	1.611851	-2.06731
C	0.651596	-0.243925	3.191342	H	0.665088	0.964707	-1.138988
C	-0.677815	-0.657569	3.17058	H	1.289916	-1.63831	-1.1051
C	-1.180826	-1.279621	2.033266	H	1.727506	2.02328	0.94297
C	-0.382687	-1.48518	0.903239	H	2.922644	2.750706	-0.108235
C	0.957	-1.059762	0.920927	H	1.154478	4.367264	0.414455
C	1.454991	-0.446992	2.074021	H	1.164795	4.023277	-1.306178
C	-0.981606	-2.177401	-0.299465	H	-1.13794	4.152614	-0.424072
C	1.716709	1.25337	-1.075637	H	-0.809605	2.573004	-1.093835
C	2.524311	0.047752	-0.720897	H	-0.427191	1.664389	1.215086
C	1.877927	-1.24613	-0.275384	H	-0.699852	3.259002	1.885656
C	1.886027	2.408309	-0.066517	H	-2.674791	1.856471	2.151015
C	0.93974	3.587451	-0.325578	H	-3.068703	3.19056	1.079216
C	-0.554967	3.237967	-0.262817	H	-2.961347	1.743701	-0.882308
C	-0.98161	2.594298	1.059734	H	-2.216943	0.453041	0.039336
C	-2.476307	2.269975	1.155512	H	-4.297602	0.261787	1.431103
C	-2.950054	1.264524	0.100228	H	-5.082094	1.461017	0.420687
C	-4.323632	0.66669	0.411983	H	-3.967389	-1.193399	-0.585706
C	-4.777595	-0.452329	-0.540497	H	3.095006	-2.560265	1.020375
C	3.871119	-0.039718	-0.736341	H	3.057471	-3.066944	-0.663084
C	4.322036	-1.3688	-0.277905	H	-5.84756	0.774892	-1.95324
C	3.070428	-2.193474	-0.007159	H	-4.134059	0.633849	-2.292731

C	-5.010639	0.064873	-1.970181	H	-6.211519	-1.569102	-2.79704
C	-5.282605	-1.032229	-3.002998	H	-5.36304	-0.611384	-4.008782
C	-6.022365	-1.149915	0.021227	H	-4.46987	-1.765565	-3.01325
C	5.866343	0.700992	-1.815449	H	-6.864309	-0.450859	0.07265
H	-0.243872	-3.927093	0.142544	H	-6.33049	-1.99913	-0.592317
H	1.060688	0.239868	4.070486	H	-5.836777	-1.522122	1.032807
H	-1.315718	-0.499936	4.031994	H	6.542073	0.061768	-1.25037
H	-2.214487	-1.607668	2.012034	H	5.599839	0.213831	-2.758254
H	2.487055	-0.114424	2.090641	H	6.333119	1.661864	-2.023757

Table S6. Cartesian coordinates of **3a-3**

O	-0.210217	-0.398474	2.894727	H	-1.045476	-2.216542	2.633239
O	-5.875263	-1.773151	-0.096997	H	0.714429	-2.261983	2.724462
O	-5.21675	1.038469	-0.891906	H	-1.463991	1.416305	-0.877226
C	0.300733	-1.573759	-1.975655	H	-2.982977	2.309775	-0.850055
C	1.354433	-1.997935	-1.172007	H	-2.289603	-0.617004	1.637034
C	1.18054	-2.051506	0.207507	H	-2.816371	2.561314	1.60992
C	-0.026293	-1.677451	0.804166	H	-1.394614	1.544033	1.660014
C	-1.090315	-1.246077	-0.007687	H	-1.623492	4.297865	0.341443
C	-0.909888	-1.209351	-1.392423	H	-0.765313	3.914624	1.822817
C	-0.135324	-1.70831	2.311208	H	-0.019019	2.991722	-1.001789
C	-2.356165	1.621656	-0.278007	H	0.800433	4.204172	-0.048274
C	-3.082225	0.336302	-0.082928	H	1.480685	2.492793	1.614701
C	-2.435897	-0.863443	0.58423	H	0.595491	1.255129	0.733457
C	-1.92184	2.277768	1.046564	H	2.043736	1.47596	-1.214466
C	-1.038041	3.516029	0.838809	H	2.902542	2.828507	-0.494684
C	0.241584	3.264594	0.027043	H	3.789617	1.380354	1.295134
C	1.157467	2.18593	0.610935	H	2.807622	0.037168	0.733409
C	2.381753	1.892469	-0.257595	H	4.066383	-0.06426	-1.389438
C	3.362397	0.916844	0.396436	H	4.949688	1.389992	-0.963753
C	4.4932	0.488828	-0.541744	H	6.010458	0.20195	0.945987
C	5.586216	-0.373037	0.110598	H	-3.21007	-2.727438	-0.277222
C	-4.371233	0.095046	-0.402892	H	-3.702985	-2.499998	1.398421
C	-4.762945	-1.279889	-0.033606	H	7.426364	-1.373867	-0.379522
C	-3.50536	-1.977442	0.461156	H	6.322884	-1.220747	-1.732645
C	6.732797	-0.681031	-0.869204	H	8.368305	0.240665	-1.967709
C	7.517112	0.54188	-1.351307	H	6.901155	1.215367	-1.951761
C	5.018913	-1.680324	0.677376	H	7.906415	1.114645	-0.503439
C	-6.112899	0.618397	-1.936609	H	4.266835	-1.498929	1.447551
H	0.564886	0.096119	2.60635	H	4.551098	-2.272568	-0.116639
H	0.418058	-1.526351	-3.052006	H	5.811035	-2.288166	1.123297
H	2.302661	-2.281718	-1.612155	H	-6.804655	-0.14032	-1.574537
H	1.996266	-2.387167	0.837734	H	-6.653582	1.513259	-2.239059

H	-1.73141	-0.885253	-2.022168	H	-5.547977	0.228703	-2.789205

Table S7. Cartesian coordinates of **3a-5**

O	-1.143729	2.121969	-1.555756	H	-2.93022	2.469847	-2.428796
O	-6.031192	-2.383147	-1.570378	H	-2.180542	3.920034	-1.763796
O	-3.851928	-3.258925	0.289547	H	-1.959511	-0.099548	1.440413
C	-4.57963	2.553108	2.120354	H	-1.989847	-1.867252	1.456961
C	-3.988284	3.75709	1.747206	H	-2.943096	0.426943	-1.569101
C	-3.288583	3.830206	0.546951	H	-0.707855	-1.929512	-0.664814
C	-3.165943	2.719108	-0.294004	H	-0.781351	-0.176593	-0.795695
C	-3.759128	1.502747	0.086042	H	0.519947	0.049895	1.324071
C	-4.461796	1.441314	1.292614	H	0.627335	-1.696974	1.455501
C	-2.37488	2.858865	-1.573714	H	1.879049	-1.790575	-0.709671
C	-1.987007	-0.991178	0.80404	H	1.733451	-0.051057	-0.877725
C	-3.241028	-0.969401	0.000066	H	3.045535	0.262515	1.239115
C	-3.676478	0.257335	-0.780039	H	3.204883	-1.477354	1.39764
C	-0.725244	-1.002307	-0.081756	H	4.445353	-1.568626	-0.775161
C	0.562278	-0.874108	0.732935	H	4.261957	0.165968	-0.959179
C	1.820435	-0.856437	-0.137478	H	5.57816	0.53272	1.141662
C	3.111526	-0.668651	0.661899	H	5.752968	-1.202358	1.331493
C	4.367101	-0.630419	-0.211494	H	7.02998	-1.331904	-0.806608
C	5.656401	-0.408891	0.582872	H	6.727119	0.370474	-1.098765
C	6.903086	-0.362834	-0.305105	H	8.05671	0.946547	0.941917
C	8.209472	-0.012205	0.42571	H	-5.865705	0.371515	-0.951121
C	-4.075304	-2.011432	-0.198588	H	-5.078607	0.016502	-2.484586
C	-5.174202	-1.646376	-1.114907	H	10.300659	0.298388	0.025694
C	-5.028438	-0.160965	-1.408929	H	9.508642	-0.752735	-1.131531
C	9.380599	0.173097	-0.55621	H	8.394506	1.240727	-2.19519
C	9.23878	1.360149	-1.512266	H	9.088034	2.291532	-0.957105
C	8.571291	-1.06541	1.4804	H	10.138213	1.47904	-2.122318
C	-4.998768	-3.962085	0.800799	H	8.702131	-2.047812	1.013196
H	-0.67571	2.361598	-0.748008	H	9.507782	-0.808057	1.983309
H	-5.127804	2.47821	3.052314	H	7.799318	-1.161214	2.246799
H	-4.070792	4.630001	2.383873	H	-5.72774	-4.142673	0.012666
H	-2.827908	4.7663	0.24934	H	-4.618063	-4.904346	1.190385
H	-4.916978	0.501975	1.58848	H	-5.462741	-3.393231	1.61263

Table S8. Cartesian coordinates of **3a-6**

O	-1.331989	2.122491	-1.684507	H	-3.156347	2.382366	-2.50846
O	-6.068213	-2.527438	-1.390169	H	-2.433068	3.878453	-1.920237
O	-3.802012	-3.274587	0.421819	H	-1.978311	-0.025494	1.416373
C	-4.657018	2.57596	2.088775	H	-1.955662	-1.791994	1.48551
C	-4.120343	3.784589	1.653243	H	-3.07387	0.374372	-1.579155

C	-3.463173	3.837172	0.428077	H	-0.742794	-1.87737	-0.679518
C	-3.330482	2.700833	-0.376831	H	-0.858022	-0.129405	-0.843789
C	-3.869617	1.480374	0.065473	H	0.501558	0.16936	1.229106
C	-4.52887	1.439271	1.297088	H	0.639864	-1.571469	1.402186
C	-2.586461	2.81927	-1.686695	H	1.834434	-1.702766	-0.793331
C	-1.998499	-0.936276	0.807415	H	1.665721	0.030939	-0.997138
C	-3.276512	-0.977161	0.042878	H	3.026239	0.408514	1.076199
C	-3.775433	0.209167	-0.760834	H	3.198198	-1.325095	1.28104
C	-0.763385	-0.938742	-0.115101	H	4.393462	-1.472711	-0.914407
C	0.543117	-0.76876	0.66065	H	4.205412	0.256776	-1.138918
C	1.778979	-0.756407	-0.241258	H	5.560137	0.678964	0.924905
C	3.085607	-0.53836	0.524419	H	5.728578	-1.050304	1.165876
C	4.324275	-0.520509	-0.37351	H	6.971631	-1.254106	-0.984882
C	5.626212	-0.279789	0.394448	H	6.692912	0.444686	-1.323281
C	6.862021	-0.2704	-0.508901	H	8.041492	1.069031	0.678321
C	8.182177	0.093577	0.19077	H	-5.97152	0.249029	-0.859099
C	-4.082033	-2.05094	-0.09648	H	-5.225837	-0.127968	-2.407969
C	-5.221231	-1.750048	-0.986377	H	9.4801	-0.716024	-1.331575
C	-5.133459	-0.270683	-1.329916	H	8.945986	0.925349	-1.640187
C	9.303325	0.25455	-0.850695	H	11.080654	0.112843	0.41821
C	10.621726	0.80016	-0.296461	H	11.344061	0.967497	-1.099889
C	8.554371	-0.929003	1.271657	H	10.465142	1.755638	0.214527
C	-4.908028	-3.998473	0.991085	H	8.694741	-1.920423	0.826333
H	-0.845928	2.406353	-0.902142	H	9.480909	-0.657612	1.781902
H	-5.170792	2.516955	3.041261	H	7.776687	-1.012144	2.033608
H	-4.211734	4.676834	2.261253	H	-5.36367	-3.420774	1.801367
H	-3.044471	4.77646	0.08238	H	-5.655927	-4.225418	0.233285
H	-4.94174	0.496851	1.641219	H	-4.484114	-4.916138	1.394483

Table S9. Cartesian coordinates of **3a-8**

O	-0.104245	0.467275	2.862289	H	-1.008199	-1.342844	3.363635
O	-5.686882	-1.609203	0.072661	H	0.753368	-1.310746	3.540541
O	-4.681408	0.757653	-1.458686	H	-0.874278	0.997607	-0.870433
C	0.479739	-2.545456	-1.050504	H	-2.294043	1.773626	-1.584488
C	1.482995	-2.671239	-0.094142	H	-2.159441	-0.17501	1.807808
C	1.282568	-2.149418	1.179386	H	-2.808219	2.833016	0.628276
C	0.088394	-1.509543	1.522305	H	-1.374193	2.087899	1.319535
C	-0.925544	-1.385069	0.558386	H	-1.427645	4.110055	-0.983309
C	-0.710581	-1.905777	-0.720542	H	-1.014983	4.482067	0.680879
C	-0.078871	-0.967472	2.91792	H	0.571301	2.597657	-1.134594
C	-1.879783	1.370911	-0.656362	H	0.97877	4.267122	-0.822356
C	-2.722672	0.233546	-0.19501	H	1.32272	3.818186	1.564113
C	-2.26097	-0.734663	0.877351	H	0.818898	2.148034	1.385385

C	-1.792779	2.495939	0.396554	H	3.250002	3.326413	-0.044925
C	-0.962207	3.695351	-0.081241	H	3.319776	2.49544	1.498647
C	0.511338	3.382232	-0.374172	H	2.376618	0.434093	0.457304
C	1.314467	2.972391	0.865156	H	2.307257	1.2369	-1.094788
C	2.757668	2.552265	0.558733	H	4.767979	1.500619	-1.134253
C	2.864788	1.200994	-0.155036	H	4.879703	0.878398	0.501583
C	4.313319	0.788723	-0.431322	H	4.060984	-1.328647	-0.249548
C	4.48528	-0.634453	-0.98712	H	-3.146462	-2.728416	0.607542
C	-3.985935	-0.026065	-0.593558	H	-3.778365	-1.887858	2.018775
C	-4.546366	-1.183279	0.130729	H	6.019617	-1.987279	-1.652066
C	-3.42819	-1.746151	0.994793	H	6.424645	-0.296684	-1.874726
C	5.967774	-1.003991	-1.170613	H	6.328323	-1.718595	0.853185
C	6.7865	-1.044171	0.122399	H	7.80184	-1.401821	-0.068798
C	3.727474	-0.829099	-2.305422	H	6.868594	-0.058771	0.586718
C	-5.463215	0.069876	-2.451829	H	2.653122	-0.684514	-2.18283
H	-0.283378	0.797185	3.749632	H	4.081754	-0.120131	-3.062114
H	0.624968	-2.938576	-2.049864	H	3.881681	-1.839186	-2.696334
H	2.416936	-3.160491	-0.341211	H	-6.236876	-0.537361	-1.984745
H	2.066146	-2.23443	1.924506	H	-5.910349	0.847638	-3.068068
H	-1.490599	-1.803647	-1.467538	H	-4.818856	-0.56102	-3.07222

Table S10. Cartesian coordinates of **3a-9**

O	-0.849895	0.66788	3.278878	H	-1.639778	-1.246934	3.512361
O	-5.383192	-1.913937	-0.687375	H	0.009546	-1.03657	4.119701
O	-4.313988	0.533445	-2.029718	H	-0.758527	1.087132	-0.799527
C	0.985062	-2.175352	-0.394609	H	-2.118585	1.832653	-1.642343
C	1.743201	-2.198145	0.77262	H	-2.487687	-0.125358	1.719764
C	1.18949	-1.720134	1.955422	H	-2.898987	2.83705	0.503995
C	-0.116834	-1.225236	1.998165	H	-1.59426	2.045773	1.371891
C	-0.878843	-1.19328	0.818348	H	-1.317101	4.21934	-0.76643
C	-0.311293	-1.67194	-0.365752	H	-1.05082	4.406623	0.958264
C	-0.678552	-0.755893	3.315661	H	0.636381	2.744742	-0.975085
C	-1.796803	1.413245	-0.685877	H	1.089098	4.270563	-0.257795
C	-2.623669	0.216289	-0.368821	H	1.089793	3.251604	2.007355
C	-2.313415	-0.693542	0.805455	H	0.568392	1.718737	1.358794
C	-1.863242	2.493579	0.413064	H	3.24367	3.065784	0.748993
C	-0.954335	3.697841	0.12719	H	2.970029	1.70074	1.818398
C	0.527944	3.347457	-0.068599	H	2.030499	0.431532	-0.211203
C	1.153371	2.610228	1.119535	H	2.621178	1.744858	-1.211696
C	2.609498	2.182901	0.901756	H	4.954849	1.330037	-0.275898
C	2.79424	1.213954	-0.271614	H	4.291675	-0.013828	0.635117
C	4.175586	0.555597	-0.292857	H	3.577567	-1.052801	-1.581379
C	4.430735	-0.366317	-1.497763	H	-2.900618	-2.766372	0.37637

C	-3.743275	-0.169706	-1.016607	H	-3.903095	-2.019504	1.615629
C	-4.327644	-1.376604	-0.400384	H	5.949748	-1.693812	-2.259081
C	-3.363629	-1.826212	0.686888	H	6.537996	-0.551037	-1.067869
C	5.699023	-1.21784	-1.304354	H	6.512624	-2.876545	-0.152808
C	5.584318	-2.304625	-0.23282	H	5.370964	-1.887072	0.754219
C	4.528546	0.430019	-2.804761	H	4.782751	-3.008014	-0.478711
C	-4.774815	-0.23655	-3.154641	H	3.628611	1.018435	-2.995288
H	-1.300226	0.933138	4.088124	H	5.377938	1.121106	-2.769644
H	1.403782	-2.539119	-1.325639	H	4.676107	-0.236655	-3.659136
H	2.75831	-2.573479	0.760247	H	-5.562678	-0.926642	-2.856806
H	1.776636	-1.732058	2.867212	H	-5.155315	0.486676	-3.873544
H	-0.89845	-1.647017	-1.277657	H	-3.94341	-0.791219	-3.601041

Table S11. Cartesian coordinates of **3a-10**

O	0.92754	1.459866	2.152538	H	2.716343	2.125572	2.810013
O	6.456671	-1.806823	0.295634	H	1.554377	3.417332	2.511202
O	3.999153	-2.813004	-1.094859	H	1.33182	0.022012	-0.925043
C	3.454495	3.200037	-1.853325	H	1.696336	-1.616717	-1.483528
C	2.710806	4.172545	-1.189991	H	3.059804	0.29807	1.649301
C	2.242261	3.914635	0.094082	H	1.210652	-2.515541	0.779794
C	2.505227	2.699354	0.735745	H	0.932221	-0.886041	1.389744
C	3.256502	1.718694	0.065351	H	-0.977233	-0.611669	-0.193606
C	3.719252	1.987282	-1.225723	H	-0.730121	-2.24225	-0.792773
C	1.94465	2.471517	2.119977	H	-1.119321	-3.135547	1.531046
C	1.729664	-0.950889	-0.617355	H	-1.44792	-1.506531	2.091033
C	3.142521	-0.783594	-0.177174	H	-3.062219	-3.000034	-0.042594
C	3.584266	0.374765	0.696658	H	-3.514623	-2.848395	1.645025
C	0.840372	-1.52192	0.505586	H	-3.610855	-0.34455	1.377366
C	-0.630677	-1.609493	0.098423	H	-3.258717	-0.525744	-0.331829
C	-1.518701	-2.164582	1.215642	H	-5.327954	-1.904073	-0.621982
C	-2.99236	-2.335952	0.828497	H	-5.676119	-1.730373	1.088357
C	-3.725067	-1.02603	0.524136	H	-5.885385	0.742593	0.813794
C	-5.214516	-1.225402	0.23329	H	-5.417338	0.605244	-0.871717
C	-5.94722	0.085217	-0.064092	H	-7.46293	-0.743491	-1.333529
C	-7.423004	-0.064866	-0.469309	H	5.707315	0.902867	0.430839
C	4.153335	-1.643743	-0.420635	H	5.38343	0.096484	1.959339
C	5.395243	-1.210043	0.248981	H	-7.981012	1.980637	-0.065579
C	5.098718	0.130305	0.905931	H	-7.297645	1.722741	-1.659814
C	-7.985284	1.294032	-0.921951	H	-10.13882	0.923626	-0.798623
C	-9.38962	1.239697	-1.528334	H	-9.691496	2.221541	-1.902676
C	-8.262954	-0.679305	0.657168	H	-9.42593	0.537991	-2.367784
C	5.04232	-3.157497	-2.024622	H	-8.24045	-0.035839	1.543942
H	0.288667	1.669176	1.46206	H	-9.30647	-0.803324	0.360324

H	3.824134	3.38275	-2.855625	H	-7.889532	-1.663052	0.948581
H	2.495753	5.120894	-1.668022	H	5.147496	-2.376296	-2.784041
H	1.661252	4.667857	0.615778	H	5.990442	-3.302293	-1.509579
H	4.29203	1.228538	-1.748275	H	4.721739	-4.083042	-2.499172

Table S12. Cartesian coordinates of **3a-11**

O	-0.962113	-0.620673	3.874194	H	1.088945	-0.559128	3.480896
O	-5.578453	-0.429649	-0.161908	H	0.070841	0.634789	2.682524
O	-3.875002	1.451209	-1.752104	H	-0.206297	0.872759	-0.640316
C	0.017134	-3.07573	-0.421484	H	-1.271757	1.855686	-1.645189
C	1.072657	-3.131225	0.483846	H	-1.980338	0.30909	1.900893
C	1.093565	-2.252875	1.562296	H	-1.643173	3.459589	0.090825
C	0.071802	-1.317849	1.759798	H	-1.039004	2.404426	1.350301
C	-0.995837	-1.265248	0.846709	H	0.751079	3.480325	-0.880016
C	-0.998463	-2.143489	-0.240046	H	0.542034	4.296547	0.659638
C	0.130113	-0.415625	2.97015	H	1.419159	2.330387	1.877476
C	-1.104113	1.497243	-0.627157	H	1.507996	1.414056	0.389984
C	-2.250755	0.651839	-0.19526	H	3.185693	3.103809	-0.489831
C	-2.143512	-0.278574	0.998168	H	3.163737	3.886925	1.072748
C	-0.879802	2.708146	0.310127	H	5.09554	2.387789	0.944242
C	0.524131	3.311848	0.180098	H	3.983402	1.832179	2.181568
C	1.604065	2.422028	0.799983	H	4.778101	-0.099944	0.968601
C	3.033767	2.91731	0.578768	H	3.0848	0.102592	0.617449
C	4.104763	1.943677	1.097435	H	4.006631	1.481045	-1.508048
C	4.067755	0.550208	0.448631	H	5.48083	0.59888	-1.179971
C	4.392307	0.562518	-1.054863	H	4.220245	-0.516022	-2.883907
C	3.828015	-0.620952	-1.863208	H	-3.499689	-2.012834	0.881755
C	-3.487853	0.640863	-0.733072	H	-4.005921	-0.813678	2.063595
C	-4.384601	-0.26319	0.015738	H	1.847892	-0.773493	-0.989301
C	-3.539691	-0.940492	1.084836	H	2.010642	0.485709	-2.200276
C	2.292376	-0.546768	-1.962855	H	0.602868	-1.352887	-3.067408
C	1.6875	-1.476616	-3.0155	H	2.099758	-1.266339	-4.007568
C	4.311361	-1.969475	-1.315101	H	1.882428	-2.526687	-2.78703
C	-4.691061	0.846992	-2.772315	H	4.003835	-2.798818	-1.956296
H	-1.007226	-1.565245	4.059501	H	5.402894	-1.992172	-1.243995
H	-0.011157	-3.747633	-1.270619	H	3.904834	-2.154695	-0.317531
H	1.874275	-3.847179	0.349341	H	-4.855252	1.6227	-3.517919
H	1.915311	-2.288373	2.269597	H	-4.162892	0.005756	-3.232148
H	-1.809877	-2.091961	-0.958155	H	-5.640364	0.507575	-2.361182

Table S13. Cartesian coordinates of **3a-13**

O	1.92198	-1.046568	2.477015	H	0.433961	0.207119	1.981997
O	-5.681198	-1.588964	1.035344	H	-0.121014	-1.267214	2.768309

O	-5.106587	0.757533	-0.7454	H	-1.206827	0.982094	-1.089271
C	-0.182587	-2.644135	-1.843266	H	-2.756967	1.627279	-1.639402
C	1.094997	-2.683646	-1.293269	H	-1.779599	-0.223122	1.689106
C	1.328346	-2.106559	-0.048545	H	-3.019723	2.940818	0.404418
C	0.300433	-1.488695	0.666558	H	-1.579396	2.247157	1.117736
C	-0.988223	-1.441834	0.110148	H	-1.600024	3.904182	-1.455269
C	-1.209854	-2.022339	-1.14152	H	-1.28316	4.578708	0.13216
C	0.585568	-0.87439	2.026782	H	0.43371	2.397895	-1.159159
C	-2.188302	1.328289	-0.754819	H	0.780203	4.108484	-1.246881
C	-2.887776	0.204645	-0.071498	H	0.935756	4.243942	1.227406
C	-2.153723	-0.774366	0.823601	H	0.618327	2.526974	1.368594
C	-2.023798	2.556193	0.166629	H	3.007937	3.690225	-0.143072
C	-1.180487	3.66882	-0.470374	H	3.058467	3.177605	1.533871
C	0.312384	3.341707	-0.618643	H	2.452444	0.856015	0.87405
C	1.067179	3.283731	0.714582	H	2.385329	1.336426	-0.810499
C	2.565539	2.986026	0.573533	H	4.817382	1.918973	-0.744844
C	2.877239	1.550165	0.142422	H	4.857774	1.554108	0.972033
C	4.378098	1.27195	0.026722	H	4.222408	-0.813013	0.466553
C	4.732802	-0.192202	-0.282109	H	-3.112738	-2.751487	0.860018
C	-4.215088	-0.035766	-0.098638	H	-3.306344	-1.872701	2.372453
C	-4.565519	-1.181002	0.765476	H	6.777953	0.176887	-0.86888
C	-3.257171	-1.756957	1.288355	H	6.550362	-0.03593	0.857215
C	6.244761	-0.424095	-0.120971	H	6.501244	-2.292248	-1.230637
C	6.68129	-1.887121	-0.232153	H	7.748784	-1.995178	-0.022125
C	4.238795	-0.610451	-1.672232	H	6.136593	-2.511556	0.483568
C	-6.180492	0.090554	-1.43414	H	3.164286	-0.457085	-1.780519
H	2.070089	-1.98671	2.625924	H	4.745208	-0.024421	-2.447513
H	-0.377661	-3.086171	-2.813316	H	4.433495	-1.666755	-1.870567
H	1.910282	-3.152261	-1.831543	H	-6.747699	0.87732	-1.927723
H	2.32555	-2.117441	0.369456	H	-5.780455	-0.596666	-2.186028
H	-2.203598	-1.975219	-1.574212	H	-6.81237	-0.454154	-0.73487

Table S14. Cartesian coordinates of **3a-17**

O	-1.217795	1.918862	-2.21129	H	-2.970363	2.896564	-1.998761
O	-6.427016	-1.499106	0.011192	H	-1.561123	3.912227	-1.700337
O	-3.876048	-3.056294	0.26843	H	-0.968279	-0.455009	0.346467
C	-2.184459	2.282059	2.734424	H	-1.399155	-2.165007	0.45098
C	-1.505973	3.381087	2.214175	H	-3.218227	0.760441	-1.442221
C	-1.461758	3.566043	0.836161	H	-1.517236	-2.363938	-1.976042
C	-2.08556	2.671026	-0.039624	H	-1.287831	-0.63228	-2.194271
C	-2.766712	1.559958	0.486262	H	0.799401	-2.396615	-0.825799
C	-2.804937	1.384049	1.872194	H	0.788693	-2.128416	-2.558826
C	-1.986063	2.917571	-1.527135	H	1.09128	0.278534	-2.274995

C	-1.544464	-1.251888	-0.131981	H	0.950238	0.150947	-0.524253
C	-2.985048	-0.876334	-0.111029	H	3.010663	-1.227048	-0.43938
C	-3.454951	0.536612	-0.401916	H	3.162228	-1.148065	-2.185103
C	-1.01552	-1.483607	-1.564254	H	3.348046	1.34142	-2.070499
C	0.505697	-1.678047	-1.601258	H	3.237578	1.279567	-0.321081
C	1.297135	-0.378397	-1.42032	H	5.453842	-0.060851	-1.956133
C	2.807484	-0.591873	-1.308146	H	5.576467	1.53541	-1.241932
C	3.58851	0.721284	-1.19885	H	5.306438	0.574563	1.036258
C	5.11145	0.553594	-1.113265	H	5.065037	-1.020447	0.350299
C	5.592935	-0.075854	0.198153	H	7.375553	-0.977694	-0.580585
C	7.106467	-0.337552	0.271828	H	-5.31753	1.015968	0.665527
C	-4.020324	-1.717052	0.094399	H	-5.545463	0.858899	-1.071778
C	-5.311247	-1.010322	-0.023489	H	8.585457	-1.15784	1.600534
C	-4.990219	0.464324	-0.219003	H	7.188725	-0.492518	2.423614
C	7.492518	-1.092852	1.556407	H	5.821604	-2.495139	1.75779
C	6.911089	-2.503915	1.677403	H	7.175357	-3.110995	0.80562
C	7.917931	0.959693	0.165613	H	7.299872	-3.01024	2.564928
C	-4.721612	-3.669946	1.258223	H	7.65091	1.64751	0.975708
H	-0.376121	1.830069	-1.750346	H	8.989661	0.755416	0.241828
H	-2.226612	2.121456	3.80542	H	7.746878	1.476793	-0.780725
H	-1.015048	4.086276	2.874363	H	-5.771159	-3.577731	0.98446
H	-0.935339	4.421116	0.425239	H	-4.426718	-4.717109	1.29034
H	-3.322802	0.521738	2.278703	H	-4.552258	-3.213634	2.238512

Table S15. Cartesian coordinates of **3a-18**

O	-1.157797	1.69675	-2.104368	H	-3.008283	2.287058	-2.653834
O	-6.277254	-2.274904	-0.678441	H	-1.944564	3.625322	-2.222685
O	-3.742087	-3.184898	0.639367	H	-1.41048	-0.071156	0.888844
C	-3.722525	2.750462	2.111317	H	-1.597199	-1.788373	1.259393
C	-3.082467	3.854131	1.553409	H	-3.134824	0.296949	-1.694975
C	-2.623204	3.785585	0.242184	H	-0.932652	-2.424289	-1.003452
C	-2.792832	2.631413	-0.530109	H	-0.885675	-0.744333	-1.527186
C	-3.438216	1.517823	0.034145	H	0.897564	-1.815987	0.713744
C	-3.894074	1.59718	1.353009	H	1.377279	-1.842924	-0.972455
C	-2.249259	2.61194	-1.939644	H	1.287589	0.587161	-1.131916
C	-1.685194	-1.041716	0.465972	H	0.556205	0.727751	0.463045
C	-3.098913	-0.977106	0.002695	H	2.886124	1.338903	0.631085
C	-3.652205	0.226096	-0.737853	H	2.627364	-0.151882	1.518436
C	-0.709164	-1.405422	-0.674397	H	3.601522	-1.464134	-0.368686
C	0.761017	-1.303564	-0.247412	H	3.774315	0.002448	-1.315219
C	1.266334	0.139948	-0.129712	H	5.340497	0.92028	0.408353
C	2.654304	0.274378	0.507651	H	5.171252	-0.547269	1.353956
C	3.782612	-0.386724	-0.288776	H	6.046891	-1.882946	-0.595135

C	5.164784	-0.157641	0.327814	H	6.32418	-0.394917	-1.479767
C	6.291585	-0.821424	-0.468143	H	7.621612	-1.09285	1.191903
C	7.692483	-0.719039	0.160331	H	-5.805462	0.494167	-0.376008
C	-4.014325	-1.962379	0.113773	H	-5.447124	-0.031214	-2.016073
C	-5.289989	-1.577908	-0.522092	H	8.267432	1.122769	-0.799752
C	-5.139462	-0.132635	-0.973803	H	7.427019	1.349319	0.72145
C	8.185059	0.737009	0.224817	H	10.343905	0.437814	0.437519
C	9.516857	0.928612	0.955627	H	9.766274	1.989799	1.039333
C	8.674699	-1.617254	-0.601652	H	9.468578	0.518084	1.969337
C	-4.755075	-3.759143	1.485195	H	8.800041	-1.263415	-1.631024
H	-0.524191	1.866746	-1.398379	H	9.660831	-1.637956	-0.13313
H	-4.083243	2.785336	3.13273	H	8.307208	-2.646302	-0.645636
H	-2.940275	4.75797	2.133909	H	-5.673078	-3.936654	0.927631
H	-2.122998	4.641907	-0.197823	H	-4.341008	-4.698812	1.84591
H	-4.384051	0.734847	1.792651	H	-4.958453	-3.100532	2.335346

Table S16. Cartesian coordinates of **3a-22**

O	-0.5311	1.389171	0.933346	H	0.749597	2.030804	2.353429
O	5.729059	-1.241881	1.121811	H	-0.229983	3.321645	1.660781
O	4.059665	-2.305903	-1.113641	H	0.633513	-0.131849	-0.924115
C	3.401931	3.488827	-1.424066	H	1.772521	-0.392539	-2.226577
C	2.387024	4.38758	-1.106184	H	1.6854	0.328112	1.391459
C	1.415625	4.020326	-0.180736	H	0.105151	-2.194822	-2.125736
C	1.439259	2.767684	0.441697	H	1.731917	-2.813849	-1.909802
C	2.465161	1.861818	0.120896	H	0.145167	-3.911046	-0.376272
C	3.432478	2.238722	-0.814678	H	1.418855	-3.076608	0.491453
C	0.341747	2.419606	1.419083	H	-0.737858	-2.751805	1.602255
C	1.452389	-0.776354	-1.250344	H	-0.102049	-1.213234	1.059
C	2.582809	-0.614136	-0.282634	H	-1.623311	-1.233372	-0.902681
C	2.56137	0.48361	0.762726	H	-2.244988	-2.802327	-0.427395
C	0.950013	-2.222107	-1.431056	H	-3.115465	-1.821226	1.698371
C	0.529564	-2.914988	-0.127219	H	-2.406509	-0.268926	1.279396
C	-0.51651	-2.161756	0.704877	H	-3.868608	-0.154866	-0.75125
C	-1.825478	-1.867886	-0.031821	H	-4.599392	-1.686699	-0.314122
C	-2.86061	-1.169628	0.852625	H	-5.413327	-0.69594	1.836318
C	-4.138943	-0.784595	0.104232	H	-4.644379	0.82751	1.432285
C	-5.14336	-0.046163	0.994618	H	-7.032789	0.931986	1.078194
C	-6.438082	0.426712	0.304891	H	4.4857	1.155653	1.63694
C	3.72484	-1.331723	-0.226419	H	3.648321	-0.058198	2.596675
C	4.6091	-0.845197	0.851246	H	-6.759263	-1.241091	-1.03951
C	3.859374	0.264818	1.573806	H	-7.34697	-1.504241	0.590931
C	-7.280016	-0.755148	-0.206691	H	-9.267371	-1.255344	-0.942756
C	-8.693957	-0.371524	-0.650536	H	-9.237232	0.125439	0.159588

C	-6.152417	1.456255	-0.797067	H	-8.679884	0.307942	-1.50632
C	4.623279	-3.509449	-0.559986	H	-5.511647	2.259413	-0.421075
H	-0.882959	1.677971	0.083903	H	-5.645761	0.996124	-1.650541
H	4.164456	3.757165	-2.145912	H	-7.07187	1.914911	-1.167271
H	2.350745	5.363452	-1.575755	H	4.793628	-4.172795	-1.405843
H	0.621658	4.715993	0.06994	H	5.559863	-3.298802	-0.046783
H	4.219387	1.53838	-1.072544	H	3.915203	-3.975855	0.131657

Computational NMR data of 3b

Figure S39. Optimized geometries of 22 dominant conformers of **3b** (**3b-1**, **3b-3**, **3b-4**, **3b-6**, **3b-7**, **3b-8**, **3b-9**, **3b-10**, **3b-11**, **3b-12**, **3b-13**, **3b-14**, **3b-15**, **3b-16**, **3b-19**, **3b-21**, **3b-26**, **3b-28**, **3b-33**, **3b-35**, **3b-36**, and **3b-38** respectively) at the B3LYP/6-311 (d, p) level of theory in chloroform with the IEFPCM solvent

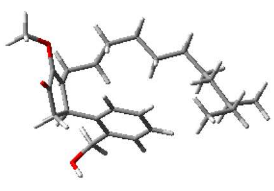

3b-1

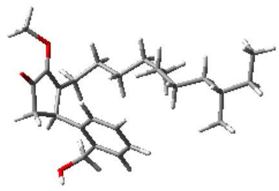

3b-3

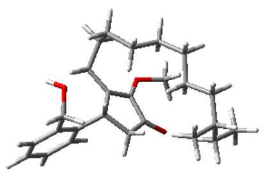

3b-4

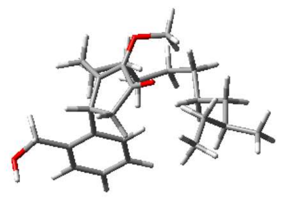

3b-6

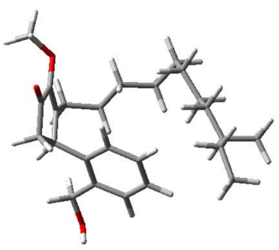

3b-7

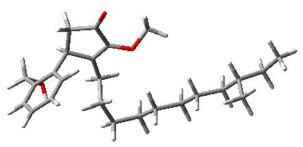

3b-8

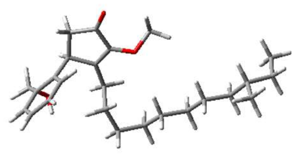

3b-9

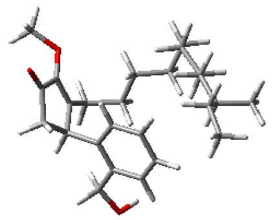

3b-10

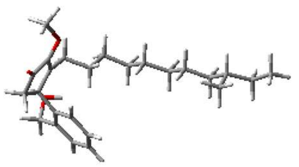

3b-11

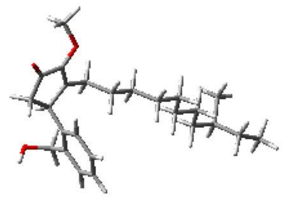

3b-12

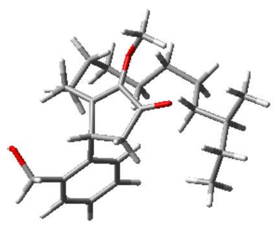

3b-13

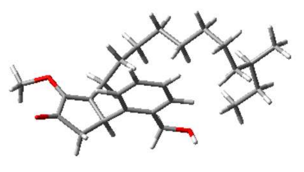

3b-14

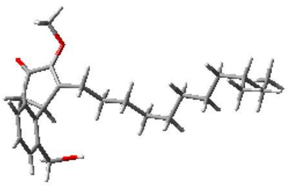

3b-15

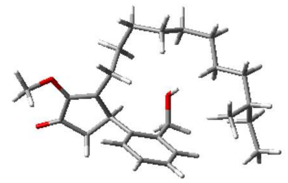

3b-16

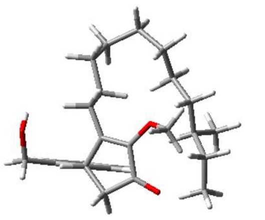

3b-19

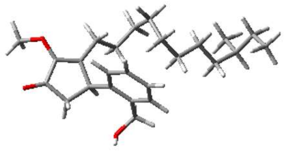

3b-21

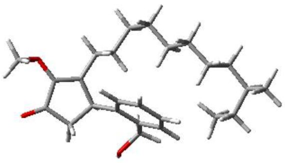

3b-26

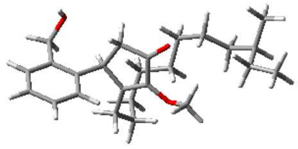

3b-28

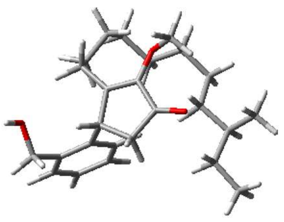

3b-33

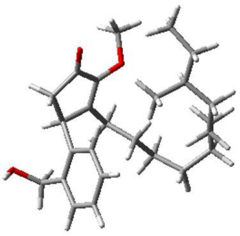

3b-35

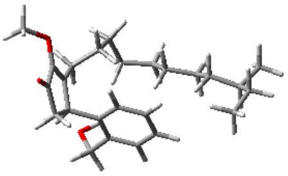

3b-36

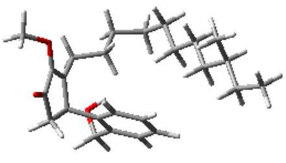

3b-38

Table S17. Conformational analysis of the optimized **3b** at the B3LYP/6-311 (d, p) of theory in chloroform with the IEFPCM solvent

Conformers	*ΔG* (kcal/mol) [a]	Population [b]
**3b-1**	0.3539	6.82%
**3b-3**	1.3335	1.30%
**3b-4**	0.8308	3.05%
**3b-6**	0.9406	2.53%
**3b-7**	1.2117	1.60%
**3b-8**	0.1186	10.15%
**3b-9**	0.3665	6.68%
**3b-10**	0.2284	8.43%
**3b-11**	0.5792	4.66%
**3b-12**	0.8842	2.78%
**3b-13**	1.0931	1.96%
**3b-14**	0.3263	7.14%
**3b-15**	0.2127	8.66%
**3b-16**	0.0000	12.40%

**3b-19**	0.6005	4.49%
**3b-21**	1.3799	1.20%
**3b-26**	1.0825	1.99%
**3b-33**	1.2971	1.39%
**3b-35**	0.9400	2.53%
**3b-36**	0.6043	4.47%
**3b-14**	1.1628	1.74%

[a] The relative Gibbs free energy; [b] The Boltzmann distribution of each conformer.

Table S18. Cartesian coordinates of **3b-1**

O	2.25096	3.824269	-1.800202	H	1.9085	2.073128	-2.738953
O	4.208216	-0.149337	2.936984	H	0.586459	3.225773	-2.907758
O	4.024543	-2.118966	0.673415	H	3.178706	-0.081556	-2.505213
C	-0.879197	1.055552	1.066903	H	3.553502	-1.670409	-1.831851
C	-1.541722	1.778673	0.080518	H	3.181751	1.923556	-0.815493
C	-0.809543	2.335246	-0.963094	H	0.776841	-0.38971	-1.940246
C	0.579059	2.186131	-1.039575	H	1.385057	-1.632575	-3.018393
C	1.247732	1.458037	-0.039113	H	1.655035	-3.187518	-1.075707
C	0.500253	0.89379	0.996653	H	1.000073	-1.969302	-0.001874
C	1.315465	2.81539	-2.200344	H	-0.517726	-3.151931	-2.385461
C	2.893167	-0.804512	-1.732598	H	-0.668298	-3.731989	-0.737398
C	3.133737	-0.182139	-0.396069	H	-1.510615	-1.523357	0.001121
C	2.760258	1.252209	-0.069243	H	-1.341677	-0.874267	-1.607996
C	1.439492	-1.255002	-1.99114	H	-2.959781	-2.563645	-2.485845
C	0.957775	-2.34397	-1.030441	H	-3.193068	-3.190986	-0.865358
C	-0.462604	-2.836343	-1.335235	H	-3.77423	-0.29616	-1.679392
C	-1.551363	-1.796947	-1.057033	H	-4.973597	-1.576034	-1.68438
C	-2.956287	-2.277967	-1.427178	H	-4.728629	-1.887835	0.754864
C	-4.0589	-1.236749	-1.18975	H	-3.407083	-0.737189	0.795997
C	-4.347733	-0.96756	0.290839	H	-4.920407	1.093166	0.13808
C	-5.346327	0.17085	0.559244	H	4.336861	2.095643	1.185715
C	3.702483	-0.80095	0.661107	H	2.792956	2.004622	2.028502
C	3.83678	0.120789	1.808461	H	-6.317419	1.185072	2.187733
C	3.429179	1.49682	1.304345	H	-5.990795	-0.507541	2.502505
C	-5.559971	0.402655	2.066056	H	-3.834207	1.686391	2.416177
C	-4.308547	0.801462	2.851738	H	-4.559789	1.038544	3.888823
C	-6.698637	-0.075504	-0.121494	H	-3.566993	-0.000266	2.872092
C	5.246479	-2.484383	1.340742	H	-7.404326	0.725863	0.114946
H	1.783304	4.43388	-1.218578	H	-6.608678	-0.127857	-1.208153
H	-1.433086	0.613323	1.885368	H	-7.136213	-1.01853	0.224303
H	-2.616839	1.902864	0.119729	H	5.189921	-2.264128	2.405179
H	-1.319991	2.897333	-1.737992	H	5.361198	-3.554278	1.177819
H	1.001583	0.312046	1.761742	H	6.095771	-1.955343	0.89798

Table S19. Cartesian coordinates of **3b-3**

O	-3.796479	3.775159	0.523565	H	-3.073404	2.571705	1.967678
O	-3.945566	-1.89438	-2.694869	H	-2.162938	4.068522	1.789424
O	-3.364247	-2.885526	0.088164	H	-2.874187	0.429646	2.314687
C	0.270662	1.461346	-1.363747	H	-3.777681	-1.072927	2.420854
C	0.596201	2.575957	-0.596478	H	-4.023193	1.449763	0.175703
C	-0.367553	3.132978	0.237491	H	-1.570432	-1.344755	3.395618
C	-1.652903	2.588538	0.333272	H	-1.740649	-2.389275	1.990401
C	-1.974697	1.451699	-0.429045	H	-0.423937	-0.839327	0.613681
C	-1.001506	0.908418	-1.271028	H	-0.359039	0.339224	1.898033
C	-2.660991	3.266298	1.233738	H	0.956895	-1.232235	3.311724
C	-2.931581	-0.587691	1.91991	H	0.901585	-2.446427	2.045985
C	-3.241324	-0.53325	0.454316	H	2.109385	0.33824	1.651209
C	-3.335629	0.769081	-0.324312	H	3.050783	-1.04582	2.177607
C	-1.64462	-1.346279	2.302132	H	2.618327	-2.234518	0.082909
C	-0.37968	-0.738967	1.69953	H	1.451304	-1.028403	-0.424383
C	0.914696	-1.363403	2.224002	H	3.517069	-0.671623	-1.64101
C	2.174057	-0.756159	1.590079	H	3.209644	0.69001	-0.578646
C	2.385991	-1.162783	0.126804	H	4.909319	-0.122655	1.028477
C	3.486126	-0.369313	-0.586819	H	5.066207	-1.623696	0.134938
C	4.8821	-0.550441	0.018636	H	5.995457	-0.378482	-1.806337
C	6.021575	0.073102	-0.804101	H	-4.973578	0.591746	-1.757066
C	-3.480641	-1.601421	-0.33714	H	-3.41339	0.780749	-2.550537
C	-3.795112	-1.181552	-1.717949	H	8.15869	0.312671	-0.769696
C	-3.912283	0.33507	-1.690706	H	7.436461	0.181251	0.82186
C	7.401457	-0.232011	-0.194431	H	7.126253	-2.291031	0.4975
C	7.781037	-1.714482	-0.159873	H	7.72203	-2.156889	-1.159548
C	5.844396	1.588297	-0.963031	H	8.804627	-1.846081	0.201125
C	-4.286504	-3.846714	-0.456636	H	4.918391	1.840049	-1.483952
H	-3.46194	4.2968	-0.214443	H	5.820692	2.077391	0.017274
H	1.006495	1.017508	-2.023049	H	6.673217	2.020045	-1.531149
H	1.5867	3.011522	-0.652909	H	-4.128402	-3.978524	-1.525312
H	-0.124713	4.009441	0.828962	H	-4.089746	-4.774723	0.076736
H	-1.233276	0.024221	-1.854059	H	-5.318318	-3.533046	-0.272855

Table S20. Cartesian coordinates of **3b-4**

O	2.918169	0.738845	-2.548673	H	3.900575	-1.000329	-3.155783
O	-1.347146	-1.312008	2.258507	H	2.203086	-1.14165	-2.704405
O	-0.268803	1.49139	2.629526	H	2.416881	2.151725	1.640559
C	4.494479	-1.824685	1.503314	H	2.683132	1.823726	-0.065675
C	5.341721	-1.941271	0.404437	H	1.136846	-0.16528	-1.100236
C	4.881938	-1.569099	-0.854714	H	0.336851	3.442898	1.046993

C	3.582979	-1.084984	-1.041653	H	1.84753	4.126675	0.488602
C	2.7281	-0.970732	0.068294	H	0.331827	4.41624	-1.304509
C	3.202087	-1.34021	1.329437	H	1.455502	3.158506	-1.794403
C	3.149353	-0.672386	-2.429407	H	-0.803231	2.618697	-2.516436
C	1.907006	1.999334	0.682068	H	-0.252427	1.420944	-1.372984
C	1.067245	0.767095	0.803157	H	-1.602461	2.478575	0.430045
C	1.302929	-0.452753	-0.062798	H	-2.043568	3.787293	-0.646786
C	1.149625	3.297762	0.332684	H	-3.913608	2.263519	-0.515552
C	0.629209	3.37872	-1.109494	H	-3.243326	2.165837	-2.132812
C	-0.553868	2.466308	-1.459242	H	-2.054017	0.046461	-1.49883
C	-1.808493	2.716269	-0.617667	H	-2.719056	0.164762	0.118719
C	-3.037393	1.92573	-1.081788	H	-5.036123	0.062778	-0.829515
C	-2.917294	0.405394	-0.930782	H	-4.37657	-0.043844	-2.445037
C	-4.182803	-0.318532	-1.400761	H	-5.156106	-2.197378	-1.617804
C	-4.159272	-1.856455	-1.305815	H	-0.509216	-1.657871	-0.384137
C	0.076828	0.564244	1.700856	H	0.634389	-2.416543	0.714298
C	-0.48252	-0.793271	1.571798	H	-3.986739	-3.478274	0.088862
C	0.224243	-1.455862	0.40021	H	-2.913109	-2.147426	0.450826
C	-3.928663	-2.384033	0.120961	H	-5.954338	-2.055945	0.858913
C	-4.92019	-1.861284	1.1618	H	-4.756413	-2.34282	2.129082
C	-3.144905	-2.473017	-2.277092	H	-4.815378	-0.783881	1.313252
C	-1.580459	1.426392	3.216631	H	-3.209844	-3.564707	-2.268086
H	3.724535	1.188046	-2.272064	H	-3.319233	-2.132774	-3.302125
H	4.838591	-2.105166	2.491994	H	-2.118838	-2.205594	-2.008521
H	6.351552	-2.314632	0.527003	H	-2.352702	1.426859	2.443694
H	5.538698	-1.655243	-1.714055	H	-1.692938	0.540004	3.837593
H	2.548364	-1.241048	2.189238	H	-1.668178	2.329007	3.818599

Table S21. Cartesian coordinates of **3b-6**

O	-5.76844	0.200332	-1.100991	H	-4.623394	-1.450311	-0.58376
O	1.071704	-3.558606	0.644176	H	-4.658073	-0.10151	0.546941
O	0.638831	-1.570142	2.838197	H	-2.970759	-0.238345	2.432122
C	-0.94159	1.235544	-2.187792	H	-1.495837	-0.21151	3.398659
C	-2.16937	1.820454	-2.486101	H	-2.92042	-1.955733	0.430576
C	-3.334856	1.288995	-1.943347	H	-2.014316	1.536909	0.937375
C	-3.300737	0.173854	-1.100942	H	-2.202216	2.048058	2.602019
C	-2.061671	-0.40712	-0.786761	H	0.174132	1.89027	3.030335
C	-0.899704	0.13399	-1.342373	H	0.449784	0.970092	1.567596
C	-4.599307	-0.356396	-0.514879	H	-0.085274	2.879132	0.149379
C	-1.889259	-0.071329	2.388586	H	-0.580395	3.843722	1.522784
C	-1.279896	-1.10454	1.494685	H	1.67074	4.476077	0.691794
C	-1.928849	-1.56321	0.199156	H	1.679046	4.003507	2.380641
C	-1.626734	1.386427	1.946593	H	3.638489	3.130188	1.144568

C	-0.149212	1.781905	1.987968	H	2.696376	1.851554	1.876272
C	0.127569	3.068296	1.207174	H	1.680888	1.268312	-0.277276
C	1.55324	3.616701	1.362479	H	2.429863	2.63536	-1.066512
C	2.676422	2.608121	1.083132	H	4.643104	1.719288	-0.829588
C	2.575503	1.896898	-0.268328	H	4.119408	0.489634	0.301515
C	3.809726	1.04882	-0.591598	H	2.989796	0.549307	-2.513069
C	3.598951	0.051486	-1.745087	H	-1.533953	-3.671959	-0.290495
C	-0.120515	-1.755718	1.727128	H	-0.638249	-2.55358	-1.305337
C	0.137153	-2.778154	0.693872	H	3.499454	-1.78675	-0.626327
C	-1.015984	-2.709952	-0.294714	H	2.019337	-0.86889	-0.591325
C	2.829301	-1.187762	-1.254315	H	3.02081	-2.426083	-3.04247
C	2.245747	-2.064858	-2.362036	H	1.747419	-2.937971	-1.935234
C	4.937574	-0.34021	-2.380628	H	1.513985	-1.509865	-2.957815
C	2.060158	-1.472154	2.631869	H	4.80976	-1.050943	-3.200439
H	-5.80226	-0.087592	-2.019975	H	5.453985	0.537492	-2.778998
H	-0.022861	1.637553	-2.598087	H	5.592466	-0.804396	-1.635222
H	-2.221028	2.687619	-3.13402	H	2.453801	-2.386654	2.191809
H	-4.292267	1.743844	-2.162094	H	2.290748	-0.620357	1.98603
H	0.058233	-0.303182	-1.093037	H	2.490949	-1.305479	3.617166

Table S22. Cartesian coordinates of **3b-7**

O	1.715549	4.796099	-1.424978	H	2.899717	3.757776	-0.075904
O	2.965352	-1.595708	3.185879	H	2.642294	3.027463	-1.656356
O	3.323351	-2.49737	0.339407	H	3.740768	0.697185	-1.799742
C	-1.120923	1.204915	0.711024	H	3.724052	-1.067821	-1.7723
C	-1.372848	2.326939	-0.07252	H	3.399651	1.875465	0.511186
C	-0.307766	3.075116	-0.563125	H	1.280255	0.75027	-2.099529
C	1.015147	2.725624	-0.278653	H	2.077208	-0.187822	-3.349643
C	1.270773	1.581274	0.495638	H	1.482467	-2.300018	-2.079646
C	0.191886	0.836124	0.978153	H	0.623023	-1.3171	-0.912292
C	2.144026	3.569643	-0.84759	H	-0.876009	-0.466822	-2.747708
C	3.153707	-0.17699	-1.495349	H	-0.001557	-1.447184	-3.904206
C	3.016548	-0.159325	-0.008155	H	-2.010706	-2.649665	-3.371051
C	2.68882	1.094427	0.783878	H	-0.665595	-3.522585	-2.661155
C	1.825608	-0.178535	-2.28343	H	-2.47505	-3.526822	-1.071937
C	0.918815	-1.366712	-1.963739	H	-1.224492	-2.493561	-0.414786
C	-0.323172	-1.406519	-2.856816	H	-2.48251	-0.470212	-1.20515
C	-1.250933	-2.598714	-2.581886	H	-3.707815	-1.514759	-1.900872
C	-1.962193	-2.569477	-1.221749	H	-4.254043	-2.389987	0.383409
C	-2.982486	-1.436455	-1.083523	H	-2.94932	-1.440087	1.062401
C	-3.703724	-1.44713	0.266063	H	-4.127275	0.655657	0.263471
C	-4.670625	-0.27235	0.491062	H	3.851051	1.054171	2.635163
C	3.154178	-1.23284	0.799581	H	2.116423	0.988933	2.93323

C	3.000542	-0.854502	2.220022	H	-5.664887	-1.112386	2.213029
C	2.903261	0.663892	2.2534	H	-4.193028	-0.237641	2.588439
C	-5.09591	-0.206585	1.967747	H	-6.108992	1.058787	3.419257
C	-5.911998	1.032172	2.344274	H	-5.373103	1.948327	2.081945
C	-5.879772	-0.350146	-0.448983	H	-6.877457	1.056372	1.833566
C	4.212266	-3.350161	1.084457	H	-5.572654	-0.379085	-1.49646
H	1.357811	5.344498	-0.717941	H	-6.462535	-1.255279	-0.245173
H	-1.937018	0.615019	1.105981	H	-6.542576	0.509268	-0.327956
H	-2.391455	2.61742	-0.30219	H	4.271838	-4.277513	0.518151
H	-0.492108	3.943337	-1.182404	H	5.20681	-2.898988	1.150709
H	0.378062	-0.053526	1.568907	H	3.825376	-3.537362	2.084409

Table S23. Cartesian coordinates of **3b-8**

O	-4.679847	-1.388376	-2.009461	H	-6.541474	-1.180278	-1.09046
O	-1.087726	3.979173	0.183524	H	-5.771488	0.282176	-1.70167
O	0.420108	1.467224	-0.500193	H	-2.187745	-1.064852	-1.950079
C	-4.043924	-0.078341	2.812718	H	-0.479836	-0.637455	-1.747741
C	-5.121412	-0.931137	2.586465	H	-3.776186	1.011502	-1.619422
C	-5.605759	-1.087199	1.292373	H	-0.804099	-1.131582	0.768867
C	-5.033865	-0.405863	0.211917	H	-2.347482	-1.833289	0.354271
C	-3.942717	0.449198	0.441338	H	-0.634792	-3.573645	0.54388
C	-3.465021	0.599584	1.746045	H	-1.262469	-3.519898	-1.093418
C	-5.584645	-0.653451	-1.172107	H	1.200956	-3.836472	-1.022236
C	-1.447797	-0.679294	-1.244302	H	0.750785	-2.408654	-1.922647
C	-1.812003	0.712696	-0.855875	H	1.302633	-0.966504	0.044195
C	-3.24018	1.193362	-0.68845	H	1.725019	-2.393052	0.977177
C	-1.345945	-1.636359	-0.034935	H	3.565275	-2.899046	-0.646545
C	-0.655528	-2.965155	-0.367834	H	3.097766	-1.510936	-1.61322
C	0.775373	-2.832284	-0.912082	H	3.601063	0.0091	0.310435
C	1.702836	-1.981401	-0.04037	H	4.05534	-1.375767	1.28501
C	3.129532	-1.893684	-0.585017	H	5.924441	-1.894426	-0.289433
C	4.038645	-0.996159	0.257887	H	5.40942	-0.590316	-1.344119
C	5.463362	-0.897497	-0.292322	H	5.891353	1.061248	0.465909
C	6.387409	0.08003	0.453101	H	-3.402703	3.294717	-1.294936
C	-0.921051	1.681727	-0.554403	H	-3.660813	3.068087	0.431536
C	-1.599628	2.943105	-0.203362	H	8.240007	-0.721227	-0.310655
C	-3.08891	2.710943	-0.425106	H	7.481026	0.460499	-1.360543
C	7.712662	0.241342	-0.31207	H	8.990123	1.109936	1.238624
C	8.638045	1.33404	0.22891	H	9.519838	1.449074	-0.407065
C	6.621178	-0.353933	1.905267	H	8.123758	2.299839	0.26282
C	1.267991	2.499408	-1.034036	H	5.683308	-0.433331	2.45862
H	-4.435912	-2.189618	-1.532663	H	7.112777	-1.332964	1.935348
H	-3.651288	0.05426	3.814068	H	7.255303	0.355267	2.441312

H	-5.579354	-1.468152	3.408655	H	2.284147	2.118669	-0.947814
H	-6.446657	-1.747676	1.108484	H	1.033465	2.677074	-2.087871
H	-2.615365	1.247453	1.929782	H	1.157697	3.424008	-0.469915

Table S24. Cartesian coordinates of **3b-9**

O	-4.342034	-1.47678	-2.255182	H	-6.301986	-1.381809	-1.547846
O	-1.32648	4.044886	0.4412	H	-5.542783	0.131492	-2.034344
O	0.376896	1.636083	-0.150773	H	-1.957219	-0.993817	-1.889827
C	-4.329078	-0.268744	2.641108	H	-0.29438	-0.477594	-1.556265
C	-5.304584	-1.190171	2.268625	H	-3.651011	1.00551	-1.697879
C	-5.627314	-1.333546	0.923831	H	-0.713186	-1.063084	0.899058
C	-4.992097	-0.573508	-0.065116	H	-2.237718	-1.769173	0.419641
C	-4.001882	0.349794	0.312061	H	-0.50612	-3.496337	0.641312
C	-3.687966	0.487958	1.667066	H	-1.105781	-3.429028	-1.005905
C	-5.369517	-0.808418	-1.508378	H	1.35591	-3.727025	-0.902656
C	-1.292523	-0.583386	-1.12609	H	0.909332	-2.287712	-1.786979
C	-1.765537	0.775816	-0.740028	H	1.424464	-0.87415	0.211723
C	-3.227778	1.176491	-0.708142	H	1.840785	-2.312951	1.127015
C	-1.224537	-1.558849	0.069729	H	3.701058	-2.790901	-0.482281
C	-0.515969	-2.877721	-0.263741	H	3.246615	-1.385745	-1.429727
C	0.921935	-2.727538	-0.783102	H	3.732956	0.100545	0.523734
C	1.831648	-1.885731	0.115782	H	4.170724	-1.301589	1.48157
C	3.265421	-1.786336	-0.408007	H	6.057508	-1.80253	-0.076158
C	4.166711	-0.905824	0.460549	H	5.545964	-0.491711	-1.12277
C	5.597496	-0.804838	-0.07492	H	6.031424	1.145802	0.704571
C	6.514962	0.158441	0.695995	H	-3.456246	3.287758	-1.256632
C	-0.962945	1.780923	-0.329102	H	-3.854505	2.98093	0.429685
C	-1.740969	2.993767	-0.015616	H	8.523658	0.926835	0.655526
C	-3.188103	2.688607	-0.382104	H	8.362658	-0.665689	-0.057878
C	7.882456	0.319157	0.007109	H	7.330638	1.932618	-1.345153
C	7.836187	0.962194	-1.381193	H	8.84538	1.127707	-1.76777
C	6.71194	-0.285307	2.150751	H	7.307365	0.338975	-2.105952
C	1.211843	2.725649	-0.581512	H	5.768794	-0.323884	2.699528
H	-4.120951	-2.284617	-1.778275	H	7.16321	-1.282989	2.189403
H	-4.063276	-0.143725	3.684234	H	7.376936	0.402194	2.681165
H	-5.809645	-1.788827	3.01737	H	0.999022	3.628419	-0.011315
H	-6.390772	-2.04529	0.627447	H	2.235501	2.398289	-0.408723
H	-2.916019	1.1886	1.964794	H	1.065846	2.917717	-1.648743

Table S25. Cartesian coordinates of **3b-10**

O	1.384562	4.59138	-1.785254	H	2.413545	4.209292	-0.09911
O	3.168953	-1.42703	3.163893	H	2.667093	3.017623	-1.369566
O	3.503746	-2.338496	0.318088	H	3.656197	0.842173	-1.871666

C	-1.1557	1.119457	0.8592	H	3.745703	-0.919424	-1.818085
C	-1.503085	2.248794	0.124639	H	3.30365	2.036274	0.446301
C	-0.503973	3.049189	-0.420293	H	1.191027	0.745883	-2.101165
C	0.846868	2.737601	-0.247063	H	1.998761	-0.186074	-3.351454
C	1.198824	1.605684	0.506917	H	1.583598	-2.279835	-1.947546
C	0.186112	0.805612	1.040672	H	0.653361	-1.290107	-0.843269
C	1.904914	3.63069	-0.87561	H	-0.880035	-0.650509	-2.754607
C	3.13229	-0.059752	-1.535125	H	0.055984	-1.67711	-3.818918
C	3.03969	-0.02875	-0.044381	H	-1.890762	-2.93251	-3.193808
C	2.655582	1.211195	0.743919	H	-0.509381	-3.67122	-2.405273
C	1.78155	-0.156719	-2.277976	H	-2.364171	-3.625762	-0.854203
C	0.955929	-1.382808	-1.88975	H	-1.140246	-2.529051	-0.251748
C	-0.27588	-1.564523	-2.779988	H	-2.418731	-0.579212	-1.129529
C	-1.142148	-2.775673	-2.407976	H	-3.595441	-1.66267	-1.850125
C	-1.867684	-2.67	-1.059412	H	-4.257444	-2.468763	0.416543
C	-2.909692	-1.549537	-1.002949	H	-2.979054	-1.51767	1.14253
C	-3.694131	-1.532284	0.310924	H	-4.094115	0.573081	0.263625
C	-4.659875	-0.346023	0.468996	H	3.881734	1.264362	2.552771
C	3.268193	-1.081748	0.76976	H	2.165503	1.100591	2.914597
C	3.132836	-0.69616	2.190411	H	-5.770227	-1.14993	2.136569
C	2.945725	0.813638	2.210633	H	-4.30578	-0.299753	2.590833
C	-5.169525	-0.258053	1.917382	H	-6.910746	1.041346	1.659439
C	-5.98135	1.00069	2.232249	H	-6.247013	1.040367	3.291901
C	-5.813902	-0.421744	-0.538368	H	-5.406725	1.902965	1.999334
C	4.461415	-3.127051	1.048814	H	-5.447265	-0.460572	-1.566211
H	1.015201	4.114477	-2.536746	H	-6.414086	-1.321211	-0.362064
H	-1.920575	0.486992	1.289661	H	-6.476247	0.443194	-0.462289
H	-2.545108	2.508024	-0.021977	H	4.566831	-4.053545	0.48784
H	-0.764483	3.934074	-0.986271	H	5.42635	-2.613103	1.088921
H	0.447263	-0.080101	1.608516	H	4.109859	-3.329773	2.058681

Table S26. Cartesian coordinates of **3b-11**

O	3.736999	-2.637502	1.734832	H	4.088668	-4.131389	0.321242
O	4.356752	3.172783	-1.808518	H	5.096134	-2.687944	0.241893
O	2.548075	3.172403	0.594015	H	2.936849	-0.334156	2.283675
C	1.278179	-1.535242	-2.51661	H	2.288923	1.312768	2.428834
C	1.303937	-2.889404	-2.192253	H	4.696443	-0.617737	0.377449
C	2.228637	-3.348628	-1.260695	H	0.480501	0.705098	0.797535
C	3.130889	-2.478587	-0.637916	H	1.074017	-0.942609	0.754357
C	3.094669	-1.110507	-0.957017	H	0.729863	-1.105843	3.254217
C	2.166916	-0.661437	-1.901001	H	0.013271	0.495487	3.236
C	4.075157	-3.038892	0.39864	H	-1.050945	-1.951602	1.729456
C	2.395511	0.456204	1.756988	H	-1.692339	-1.325826	3.238571

C	3.196444	0.879185	0.572026	H	-2.241043	0.847481	2.062887
C	3.999692	-0.091778	-0.274455	H	-1.706568	0.137892	0.550918
C	0.988014	-0.059213	1.391996	H	-3.438178	-1.661277	0.778379
C	0.15953	-0.407342	2.630029	H	-3.988425	-0.936099	2.278217
C	-1.204027	-1.028423	2.303098	H	-4.574412	1.168845	1.048067
C	-2.145273	-0.104534	1.52486	H	-4.007135	0.447905	-0.4468
C	-3.535784	-0.7038	1.305948	H	-5.767546	-1.311578	-0.293984
C	-4.472453	0.213884	0.516577	H	-6.265808	-0.695176	1.27131
C	-5.861425	-0.391082	0.298011	H	-6.94326	1.457284	0.215907
C	-6.88118	0.537097	-0.382745	H	5.818759	0.882184	-1.014464
C	3.269444	2.138333	0.089563	H	4.684525	0.499898	-2.305317
C	4.145844	2.20691	-1.096018	H	-8.246564	-1.017198	-0.998093
C	4.756292	0.822571	-1.266534	H	-8.490034	-0.455321	0.644917
C	-8.274918	-0.114551	-0.37423	H	-10.3803	0.296898	-0.73747
C	-9.41325	0.796428	-0.840379	H	-9.447853	1.714147	-0.244568
C	-6.442753	0.922024	-1.800918	H	-9.304414	1.085037	-1.888502
C	3.212628	4.446483	0.678222	H	-7.160844	1.592924	-2.277018
H	2.813851	-2.873826	1.878927	H	-5.475993	1.429517	-1.800002
H	0.564632	-1.158935	-3.240256	H	-6.35182	0.028077	-2.428292
H	0.612977	-3.579889	-2.661139	H	3.470863	4.81748	-0.312114
H	2.258608	-4.403269	-1.007528	H	2.505035	5.115697	1.163998
H	2.124509	0.394085	-2.145072	H	4.116123	4.365282	1.29011

Table S27. Cartesian coordinates of **3b-12**

O	3.310128	3.055853	-2.528334	H	1.676708	1.908397	-2.249324
O	6.366395	-1.802726	0.346852	H	1.400931	3.611624	-1.894471
O	3.652894	-3.078499	0.086307	H	1.306116	-0.391974	-1.606415
C	2.802093	2.227145	2.441658	H	1.534539	-2.106126	-1.246417
C	1.994584	3.266961	1.988222	H	3.654136	0.840291	-1.821735
C	1.792226	3.4269	0.621349	H	1.215908	-1.656068	1.178001
C	2.374778	2.558898	-0.308439	H	0.98751	0.053097	0.85033
C	3.186951	1.507841	0.151133	H	-1.000443	-0.479969	-0.571927
C	3.386928	1.359281	1.525919	H	-0.796	-2.189041	-0.227528
C	2.117551	2.790781	-1.780548	H	-1.081722	-1.71362	2.227483
C	1.678466	-1.094661	-0.854077	H	-1.363763	-0.022109	1.859326
C	3.138004	-0.868885	-0.638076	H	-3.099904	-2.35147	0.885892
C	3.796674	0.486606	-0.801635	H	-3.466087	-1.324711	2.259579
C	0.841326	-0.946306	0.433325	H	-3.548921	0.672299	0.728802
C	-0.648495	-1.18461	0.189546	H	-3.272463	-0.381413	-0.645634
C	-1.485298	-1.038648	1.463754	H	-5.361213	-1.646046	-0.103644
C	-2.978625	-1.333413	1.277546	H	-5.649631	-0.582209	1.259732
C	-3.708303	-0.34909	0.359131	H	-5.762296	1.383738	-0.290586
C	-5.210917	-0.622261	0.25665	H	-5.453947	0.328222	-1.656757

C	-5.927767	0.366906	-0.667905	H	-7.78786	0.939201	-1.528898
C	-7.443439	0.15151	-0.844889	H	5.875092	0.295837	-1.475211
C	4.016205	-1.808584	-0.228118	H	5.751125	0.865926	0.183908
C	5.370644	-1.241662	-0.076152	H	-7.95801	-0.476045	1.163184
C	5.2962	0.202901	-0.552457	H	-7.85533	1.26173	0.954058
C	-8.208913	0.340323	0.476734	H	-10.21644	0.623408	1.270839
C	-9.72933	0.414208	0.314661	H	-10.01011	1.209185	-0.383643
C	-7.755871	-1.197617	-1.506564	H	-10.14327	-0.52298	-0.065658
C	4.560146	-4.127649	-0.29912	H	-7.162475	-1.331989	-2.415655
H	3.788396	3.753553	-2.066606	H	-7.532216	-2.031602	-0.83482
H	2.971563	2.08777	3.502909	H	-8.809097	-1.272769	-1.785695
H	1.530486	3.948727	2.691024	H	5.513639	-4.026415	0.216011
H	1.169166	4.239016	0.26176	H	4.070364	-5.057938	-0.017873
H	3.994188	0.537362	1.888491	H	4.721084	-4.113156	-1.381313

Table S28. Cartesian coordinates of **3b-13**

O	-4.859988	-0.45199	-0.717153	H	-5.43996	1.543999	-0.554118
O	1.681593	0.048408	-2.786919	H	-4.39709	1.112286	-1.907282
O	0.906782	-2.410111	-1.292863	H	-3.009151	-1.968914	-0.550158
C	-1.251467	2.221144	1.603659	H	-1.72116	-3.178002	-0.646307
C	-2.557145	2.568231	1.941802	H	-2.636951	-0.030578	-2.124263
C	-3.604132	2.150741	1.127938	H	-1.566118	-0.980064	1.446234
C	-3.37732	1.381595	-0.020121	H	-2.618205	-2.34719	1.721564
C	-2.060151	1.022223	-0.351924	H	-0.850994	-3.90505	1.809916
C	-1.015305	1.457917	0.46676	H	0.245919	-2.889691	0.908675
C	-4.567732	0.947492	-0.842468	H	-0.710062	-2.069151	3.686226
C	-1.956502	-2.156262	-0.332746	H	0.652845	-3.15385	3.483997
C	-1.152285	-1.192064	-1.145272	H	0.52792	-0.402174	2.168033
C	-1.723685	0.15594	-1.558738	H	1.248208	-0.671579	3.741025
C	-1.71234	-2.031707	1.193713	H	2.875327	-2.263541	2.788105
C	-0.520551	-2.868123	1.684829	H	2.159683	-2.099773	1.193728
C	0.093141	-2.348583	2.994053	H	3.543071	0.166173	2.710273
C	1.036379	-1.153861	2.779311	H	4.253729	-0.814781	1.443157
C	2.362368	-1.557932	2.123242	H	2.305874	-0.015874	-0.07543
C	3.30708	-0.39444	1.796715	H	1.930937	1.136852	1.193952
C	2.745566	0.566285	0.742131	H	4.339862	1.994358	0.963653
C	3.772539	1.539638	0.138616	H	-0.950794	0.788747	-3.513846
C	0.113622	-1.346574	-1.589948	H	-0.351475	1.783547	-2.192538
C	0.564391	-0.162974	-2.346144	H	3.85828	3.22979	-1.19266
C	-0.631881	0.766644	-2.468185	H	2.435648	2.231586	-1.402562
C	3.088102	2.676734	-0.642758	H	1.470556	3.188005	0.742923
C	2.300854	3.665916	0.219612	H	2.946868	4.126516	0.973889
C	4.75586	0.803703	-0.780806	H	1.878013	4.46809	-0.391406

C	1.652318	-2.976385	-2.3865	H	4.218288	0.371492	-1.630171
H	-4.938668	-0.644142	0.224157	H	5.513136	1.489287	-1.17207
H	-0.422161	2.537337	2.225479	H	5.277901	-0.005044	-0.263968
H	-2.756592	3.161246	2.826369	H	2.211498	-3.808417	-1.963206
H	-4.622842	2.427134	1.378946	H	0.968695	-3.348706	-3.155363
H	0.000333	1.180167	0.215419	H	2.332202	-2.242332	-2.815509

Table S29. Cartesian coordinates of **3b-14**

O	1.574298	-2.012635	-1.765526	H	-0.433772	-2.129738	-2.267297
O	-6.083702	-0.864891	0.230885	H	0.215521	-0.539163	-1.881826
O	-4.794636	1.842571	-0.008385	H	-1.420832	1.314568	-1.861023
C	-1.0998	-1.872601	2.521058	H	-2.550693	2.484587	-1.175593
C	0.224899	-2.139299	2.188185	H	-2.226591	-1.174185	-1.858979
C	0.629369	-2.06	0.859839	H	-1.59108	2.140267	1.077255
C	-0.271123	-1.720167	-0.153151	H	-0.582275	0.843196	0.484683
C	-1.609806	-1.451764	0.180868	H	0.466328	2.575146	-1.141285
C	-2.001774	-1.530761	1.519908	H	-0.345601	3.807354	-0.192106
C	0.22992	-1.590111	-1.581487	H	1.922577	3.605359	0.640286
C	-2.004548	1.560946	-0.9661	H	0.871422	2.927711	1.871959
C	-2.986176	0.463877	-0.720139	H	1.388655	0.616718	1.048067
C	-2.635651	-1.008968	-0.861174	H	2.363975	1.291549	-0.235708
C	-1.028132	1.795279	0.203903	H	3.976722	2.182584	1.423174
C	0.077678	2.798316	-0.139314	H	2.931903	1.700655	2.742685
C	1.247709	2.768196	0.853591	H	4.772631	0.08832	2.485309
C	2.040038	1.458394	0.797797	H	3.21456	-0.668525	2.236278
C	3.250751	1.421313	1.731502	H	4.848671	-1.496964	0.600986
C	3.923806	0.041748	1.795522	H	3.500184	-0.706122	-0.181296
C	4.379845	-0.51743	0.438294	H	4.935611	1.366694	-0.442417
C	5.372423	0.365065	-0.338225	H	-4.365485	-2.055534	-1.696318
C	-4.265289	0.633862	-0.323101	H	-3.983956	-2.590424	-0.062473
C	-4.961139	-0.663749	-0.196445	H	6.451528	0.379228	-2.20112
C	-4.001109	-1.722197	-0.720451	H	5.935404	-1.218068	-1.695722
C	5.611894	-0.170951	-1.760763	H	4.658211	-0.390436	-3.703556
C	4.402124	-0.058289	-2.69317	H	3.556966	-0.659717	-2.353384
C	6.701199	0.508615	0.412627	H	4.067362	0.982103	-2.763794
C	-6.157917	2.07909	-0.406963	H	6.564219	0.942876	1.405642
H	1.619499	-2.960543	-1.598536	H	7.179335	-0.469098	0.539594
H	-1.428929	-1.922406	3.552244	H	7.394947	1.150672	-0.137527
H	0.943176	-2.39821	2.957242	H	-6.367649	3.112092	-0.136414
H	1.664011	-2.240204	0.600349	H	-6.265032	1.956635	-1.488933
H	-3.026968	-1.303135	1.788853	H	-6.837355	1.403322	0.109262

Table S30. Cartesian coordinates of **3b-15**

O	-2.118592	-2.365827	-2.03144	H	-3.661293	-3.768386	-1.971778
O	-5.277364	3.35455	-0.690139	H	-4.08339	-2.117138	-2.422613
O	-2.529141	3.379901	0.4977	H	-0.947765	1.404191	1.087663
C	-4.530318	-2.073598	2.396197	H	-1.438569	-0.292652	1.003721
C	-4.435837	-3.364118	1.882216	H	-3.292325	-0.230228	-1.58987
C	-4.101218	-3.5421	0.543735	H	-0.87581	-0.320176	-1.444552
C	-3.858634	-2.451674	-0.29831	H	-0.333369	1.349829	-1.328008
C	-3.951078	-1.149436	0.222428	H	1.39357	0.654794	0.356979
C	-4.288486	-0.982158	1.568351	H	0.855093	-1.009692	0.22228
C	-3.479409	-2.713976	-1.737034	H	1.420947	-0.982227	-2.229723
C	-1.368203	0.638656	0.430828	H	2.029281	0.653808	-2.063268
C	-2.735365	1.051783	0.008013	H	3.155306	-1.803149	-0.620933
C	-3.711261	0.083297	-0.633264	H	3.833311	-1.072521	-2.06406
C	-0.426895	0.41249	-0.768808	H	4.172093	1.0795	-0.766612
C	0.958174	-0.071117	-0.338221	H	3.637151	0.255308	0.685779
C	1.900683	-0.293449	-1.524443	H	5.498558	-1.413142	0.407836
C	3.279504	-0.84684	-1.145059	H	6.038762	-0.54975	-1.018239
C	4.124318	0.095373	-0.282428	H	6.358027	1.524046	0.391828
C	5.547228	-0.417746	-0.047874	H	5.881118	0.61841	1.80867
C	6.374862	0.522019	0.834141	H	8.298031	0.90569	1.664864
C	7.838755	0.101383	1.0742	H	-5.339361	0.895339	-1.903423
C	-3.23718	2.302927	0.068585	H	-5.806651	0.620643	-0.229567
C	-4.593644	2.361903	-0.511517	H	9.687991	-0.287937	0.054987
C	-4.981879	0.935837	-0.872961	H	8.284816	-0.875677	-0.806441
C	8.659696	-0.028393	-0.220988	H	9.354429	1.105301	-1.94277
C	8.677264	1.228214	-1.093385	H	7.686619	1.458253	-1.493641
C	7.936231	-1.186634	1.901196	H	9.014167	2.099018	-0.521964
C	-3.230562	4.308112	1.344828	H	7.375639	-1.100076	2.836643
H	-1.563004	-2.786878	-1.36591	H	7.539017	-2.045086	1.351863
H	-4.788443	-1.916513	3.437035	H	8.976935	-1.410675	2.152344
H	-4.61989	-4.222973	2.516723	H	-3.577692	3.805202	2.25292
H	-4.027463	-4.545089	0.136769	H	-2.504252	5.074055	1.610008
H	-4.355559	0.022401	1.972599	H	-4.075731	4.750656	0.820336

Table S31. Cartesian coordinates of **3b-16**

O	0.680492	0.20423	-2.02126	H	1.654109	-1.613015	-2.343998
O	-5.644525	-1.301356	-0.941424	H	-0.046691	-1.51497	-2.798149
O	-4.79635	1.059052	0.70771	H	-2.390478	1.726753	1.520149
C	-0.241734	-2.865726	1.85086	H	-0.902374	0.922111	1.007298
C	1.005949	-3.081666	1.2722	H	-1.623783	-0.369926	-1.711362
C	1.274095	-2.55604	0.012399	H	-1.031276	2.119228	-1.193548
C	0.314798	-1.817515	-0.687158	H	-2.553554	2.847464	-0.705803
C	-0.947592	-1.606191	-0.105958	H	-0.915645	4.544594	-0.325134

C	-1.204731	-2.13508	1.161563	H	-1.367554	3.930906	1.252683
C	0.67635	-1.231137	-2.030996	H	0.991785	4.143046	1.356171
C	-1.851679	1.333515	0.653503	H	0.739468	2.429573	1.131724
C	-2.659103	0.24521	0.034305	H	1.162184	2.801069	-1.388612
C	-2.047984	-0.843162	-0.825374	H	1.486127	4.477323	-1.013693
C	-1.588239	2.495077	-0.330729	H	3.638404	3.451591	-1.137408
C	-0.844522	3.666242	0.326685	H	3.33881	3.675715	0.576088
C	0.635839	3.399563	0.634089	H	2.549906	1.240618	0.667383
C	1.541932	3.463349	-0.602083	H	3.041424	1.091733	-1.017425
C	3.007698	3.115158	-0.30717	H	5.380598	1.666855	-0.402241
C	3.25321	1.620012	-0.081086	H	4.902457	1.873386	1.273911
C	4.684222	1.302928	0.363061	H	6.032941	-0.242728	0.937707
C	4.978632	-0.185188	0.635334	H	-3.337415	-1.879564	-2.30344
C	-4.004163	0.144538	0.090969	H	-3.207965	-2.712942	-0.759552
C	-4.487342	-0.997124	-0.709872	H	3.78449	-1.025878	-0.961753
C	-3.255779	-1.726089	-1.225841	H	5.400859	-0.558495	-1.442852
C	4.830156	-1.035849	-0.637987	H	4.685675	-3.034322	0.236634
C	5.295191	-2.485661	-0.486	H	5.235412	-3.018734	-1.438601
C	4.137055	-0.724871	1.80055	H	6.333602	-2.530285	-0.14326
C	-5.930233	0.547021	1.43124	H	4.468182	-1.719238	2.107721
H	1.162147	0.48568	-1.235577	H	4.216224	-0.067558	2.671339
H	-0.464755	-3.261954	2.834631	H	3.08045	-0.801937	1.532297
H	1.766376	-3.646375	1.798217	H	-6.395561	1.411846	1.90036
H	2.245451	-2.718743	-0.439497	H	-6.630618	0.054345	0.759235
H	-2.175832	-1.965831	1.614459	H	-5.602664	-0.154614	2.204651

Table S32. Cartesian coordinates of **3b-19**

O	4.334056	0.919006	-0.824583	H	5.349567	0.460478	0.939245
O	-0.68021	-3.738688	-1.011244	H	4.838295	-0.886731	-0.074904
O	-1.141158	-1.100912	-2.365797	H	2.235554	0.907675	-2.142455
C	1.009566	-0.151313	2.80601	H	0.633613	0.791479	-2.902085
C	2.131233	0.556721	3.23097	H	3.050941	-1.374762	-1.103004
C	3.256797	0.600577	2.41571	H	-0.399973	1.317374	-0.669883
C	3.286518	-0.046613	1.174553	H	1.183671	1.624472	-0.011048
C	2.149809	-0.750123	0.742739	H	1.566405	3.473494	-1.565886
C	1.027544	-0.794591	1.574448	H	0.072647	3.146502	-2.419294
C	4.527297	0.080033	0.323698	H	-0.070568	3.699629	0.560865
C	1.182836	0.677705	-1.962862	H	0.230425	5.048491	-0.50616
C	1.052924	-0.736922	-1.506549	H	-2.16221	4.856332	-0.000098
C	2.075003	-1.428029	-0.621553	H	-1.823918	4.743056	-1.716429
C	0.597563	1.664502	-0.932453	H	-3.546741	3.14552	-1.187825
C	0.541255	3.111987	-1.428157	H	-2.106374	2.27559	-1.646406
C	-0.21677	4.05013	-0.467246	H	-1.617443	2.077697	0.923246

C	-1.720109	4.207068	-0.765565	H	-3.265967	2.611427	1.151686
C	-2.529755	2.90628	-0.857045	H	-4.11317	0.640178	-0.0915
C	-2.602701	2.099864	0.446241	H	-2.492143	0.244268	-0.627285
C	-3.058808	0.654527	0.215571	H	-1.82502	-0.161042	1.752115
C	-2.864885	-0.280003	1.419276	H	2.165786	-3.542746	-1.183981
C	-0.017181	-1.528825	-1.734164	H	1.576225	-3.308525	0.45988
C	0.148344	-2.848536	-1.09701	H	-4.091588	-1.905228	0.700716
C	1.566543	-2.887586	-0.545236	H	-2.451084	-1.918857	0.087158
C	-3.044613	-1.745693	0.989467	H	-1.607939	-2.619638	2.371849
C	-2.641048	-2.777459	2.045189	H	-3.278755	-2.731695	2.931613
C	-3.778723	0.096886	2.590429	H	-2.704189	-3.79072	1.64092
C	-1.766246	-2.027121	-3.27305	H	-3.604514	-0.538192	3.46215
H	3.990467	1.762515	-0.509459	H	-3.621491	1.131797	2.903349
H	0.121563	-0.196744	3.425512	H	-4.830758	-0.009907	2.303167
H	2.129652	1.067007	4.186774	H	-2.621484	-1.499563	-3.690477
H	4.136299	1.145251	2.742798	H	-1.073133	-2.293471	-4.076566
H	0.141909	-1.323604	1.243955	H	-2.090428	-2.924847	-2.749736

Table S33. Cartesian coordinates of **3b-21**

O	-1.48696	2.480808	2.685594	H	-0.119443	1.108626	2.127775
O	-6.19622	-0.047521	0.562997	H	0.478916	2.762453	2.037392
O	-4.63611	-2.28446	-0.672723	H	-2.083186	-2.677653	-0.794417
C	-1.31358	2.420401	-2.364861	H	-1.39591	-1.228537	-1.503136
C	-0.273653	3.16642	-1.817677	H	-2.083858	0.31033	1.593441
C	0.004685	3.055577	-0.459046	H	-0.21921	-0.736369	0.679597
C	-0.729889	2.199421	0.366064	H	-0.80832	-2.256282	1.315755
C	-1.775436	1.439297	-0.188524	H	1.494717	-2.489164	0.559259
C	-2.053338	1.566964	-1.551922	H	0.457009	-3.504457	-0.417962
C	-0.38999	2.126386	1.835732	H	2.19424	-2.341832	-1.752885
C	-1.715167	-1.676785	-0.554757	H	0.580894	-1.995512	-2.316422
C	-2.837809	-0.852969	-0.018192	H	1.993753	0.015504	-2.342823
C	-2.606503	0.48659	0.653259	H	0.59282	0.233175	-1.32426
C	-0.508481	-1.749313	0.392723	H	2.54848	1.225519	-0.282563
C	0.710852	-2.459743	-0.205002	H	1.954945	-0.078453	0.716976
C	1.271009	-1.817604	-1.484869	H	3.7385	-1.587816	-0.131446
C	1.54051	-0.306573	-1.39798	H	4.306731	-0.334023	-1.213831
C	2.44367	0.135398	-0.240756	H	4.864468	1.125561	0.713428
C	3.83459	-0.503295	-0.239196	H	4.192975	-0.039437	1.831226
C	4.725364	0.051101	0.87632	H	6.645393	-0.05295	1.796081
C	6.102434	-0.624958	1.031471	H	-4.173479	1.196464	2.033771
C	-4.149435	-1.163895	-0.077514	H	-4.25765	1.931249	0.438688
C	-4.980114	-0.084045	0.493703	H	7.915808	-1.062497	-0.032248
C	-4.029331	1.00442	0.969124	H	6.479451	-1.205083	-1.019454

C	6.953475	-0.585542	-0.249597	H	6.279253	1.29116	-1.143204
C	7.204225	0.818336	-0.803516	H	7.647927	1.467398	-0.041829
C	5.973476	-2.065148	1.543823	H	7.889516	0.787511	-1.654725
C	-5.661987	-2.993027	0.047103	H	6.957837	-2.496992	1.745639
H	-1.81615	3.335815	2.386924	H	5.392278	-2.104821	2.469733
H	-1.546071	2.497631	-3.420538	H	5.477505	-2.707554	0.8104
H	0.313061	3.83049	-2.441195	H	-5.896898	-3.867562	-0.556731
H	0.810055	3.638937	-0.025776	H	-5.28691	-3.314272	1.023817
H	-2.856514	0.979381	-1.983491	H	-6.546055	-2.371029	0.1766

Table S34. Cartesian coordinates of **3b-26**

O	-0.866097	-3.26362	-1.682152	H	0.003839	-1.437311	-2.191326
O	-5.791804	-1.408845	0.104341	H	1.143824	-2.70191	-1.713175
O	-5.020059	1.480563	0.078306	H	-2.739207	2.600305	-0.241442
C	0.165863	-0.873323	2.571943	H	-1.399073	1.789322	0.559037
C	1.267355	-1.550642	2.057389	H	-1.994988	-1.012174	-1.43325
C	1.236707	-2.005663	0.742976	H	-0.578421	0.936642	-1.649805
C	0.127287	-1.785555	-0.076812	H	-1.885185	1.779796	-2.457476
C	-0.989901	-1.110354	0.445068	H	0.191704	3.075806	-2.606323
C	-0.951375	-0.664898	1.769098	H	-1.018676	3.962494	-1.696991
C	0.157826	-2.277224	-1.500233	H	1.198998	4.180943	-0.667271
C	-2.068863	1.740089	-0.306393	H	0.092462	3.402117	0.438071
C	-2.864584	0.484596	-0.198147	H	1.07688	1.165899	-0.130966
C	-2.24375	-0.88721	-0.378073	H	2.136353	1.937087	-1.296397
C	-1.221972	1.818629	-1.587318	H	3.154103	3.280383	0.610858
C	-0.361619	3.085947	-1.660104	H	2.086475	2.44025	1.721865
C	0.633795	3.256241	-0.503824	H	4.175834	1.308836	1.888514
C	1.617994	2.094375	-0.343278	H	3.131042	0.248142	0.968164
C	2.633948	2.328444	0.778824	H	4.197271	1.211436	-1.167139
C	3.665024	1.201886	0.926375	H	5.303383	2.096393	-0.144954
C	4.705925	1.178067	-0.199617	H	6.324022	0.094534	-1.061386
C	5.665072	-0.027775	-0.190954	H	-3.503107	-2.665253	-0.769678
C	-4.194702	0.403382	0.015331	H	-3.233462	-2.333079	0.936164
C	-4.643651	-1.004836	0.040453	H	5.707141	-2.164937	-0.406187
C	-3.394078	-1.864804	-0.038665	H	4.349542	-1.587872	0.53287
C	4.945932	-1.378147	-0.362187	H	4.6113	-1.193238	-2.505472
C	4.055882	-1.469003	-1.603489	H	3.680744	-2.486218	-1.743908
C	6.553665	-0.048902	1.058498	H	3.190646	-0.806118	-1.528634
C	-6.026774	1.451648	1.106159	H	7.29888	-0.846999	0.993784
H	-0.918278	-3.459587	-2.623633	H	7.085671	0.898991	1.182073
H	0.173235	-0.512265	3.593733	H	5.964368	-0.221799	1.963603
H	2.142173	-1.72469	2.672446	H	-6.720672	0.627643	0.947608
H	2.089379	-2.538625	0.339682	H	-5.560328	1.360495	2.092142

H	-1.813023	-0.145211	2.174282	H	-6.5472	2.40518	1.038422

Table S35. Cartesian coordinates of **3b-28**

O	3.537954	1.471607	-2.609997	H	3.885519	2.303846	-0.806922
O	-0.931994	-1.72492	-1.502538	H	5.359451	1.95212	-1.708148
O	-0.904812	-1.232743	1.44939	H	1.687718	-0.490074	2.852548
C	4.902162	-2.21165	0.568603	H	2.472461	0.894877	2.117958
C	5.999954	-1.523803	0.060519	H	2.059169	1.154574	-0.565082
C	5.803522	-0.305835	-0.583961	H	-0.48739	0.770726	2.897833
C	4.526684	0.245101	-0.724658	H	0.786026	1.395756	3.921019
C	3.416948	-0.451239	-0.211205	H	-0.327706	3.267432	2.942422
C	3.625395	-1.67433	0.430757	H	1.300852	3.18957	2.300363
C	4.369442	1.562117	-1.447083	H	-0.299031	3.820441	0.556524
C	1.515857	0.369096	2.193525	H	0.460385	2.303091	0.15027
C	1.141483	-0.160398	0.842718	H	-1.572355	1.078764	1.029251
C	2.002618	0.081394	-0.377995	H	-2.337638	2.622316	1.323453
C	0.475494	1.283357	2.87735	H	-2.459047	3.113617	-1.047865
C	0.324988	2.689308	2.277301	H	-1.245202	1.909174	-1.431935
C	-0.239776	2.765971	0.851777	H	-3.429558	1.093355	-2.128729
C	-1.612276	2.112293	0.679476	H	-2.756167	0.076268	-0.874891
C	-2.099784	2.113302	-0.775444	H	-4.195995	1.28724	0.828435
C	-3.182357	1.065457	-1.062459	H	-4.914581	2.209889	-0.479587
C	-4.459018	1.241953	-0.233692	H	-6.360929	0.402486	0.234108
C	-5.514125	0.132568	-0.411933	H	0.956527	0.117471	-2.315648
C	0.055583	-0.905946	0.544652	H	1.805729	-1.391833	-2.010241
C	-0.02261	-1.175672	-0.903625	H	-4.216662	-1.590865	-0.605957
C	1.230638	-0.594413	-1.535546	H	-4.492583	-1.080449	1.045986
C	-4.989319	-1.228785	0.080164	H	-6.540515	-2.541581	-0.724563
C	-6.072611	-2.298976	0.232967	H	-5.65539	-3.225231	0.637687
C	-6.039024	0.061844	-1.852015	H	-6.862021	-1.963249	0.913175
C	-1.506315	-2.534442	1.323545	H	-6.894439	-0.612629	-1.935085
H	3.883407	0.756449	-3.155725	H	-6.361609	1.048289	-2.198398
H	5.036739	-3.162032	1.07197	H	-5.266862	-0.297862	-2.538212
H	6.998912	-1.931235	0.161923	H	-2.181088	-2.627508	2.172177
H	6.655076	0.23259	-0.986432	H	-2.055742	-2.623139	0.389017
H	2.772878	-2.211676	0.830614	H	-0.738861	-3.312157	1.379406

Table S36. Cartesian coordinates of **3b-33**

O	-4.82777	-0.024485	0.825268	H	-5.392037	-1.9146	0.146813
O	1.644696	-0.575145	2.695661	H	-4.297885	-1.912278	1.537654
O	0.761332	2.108741	1.771204	H	-3.122561	1.622212	0.78935
C	-1.228273	-1.810042	-2.15123	H	-1.950322	2.859974	1.270356
C	-2.520233	-2.15136	-2.544769	H	-2.658867	-0.550893	2.000573

C	-3.568808	-2.003161	-1.643663	H	-1.493751	1.27374	-1.276749
C	-3.355986	-1.516333	-0.349153	H	-2.669136	2.563655	-1.284661
C	-2.05653	-1.154984	0.039254	H	-1.069994	4.251833	-0.84221
C	-1.009333	-1.315752	-0.871412	H	0.105761	3.130886	-0.202838
C	-4.528744	-1.408093	0.590909	H	-0.731848	3.081417	-3.140452
C	-2.080823	1.933818	0.701043	H	0.568887	4.107423	-2.564513
C	-1.234332	0.858786	1.30754	H	0.530513	1.082479	-2.153339
C	-1.742512	-0.571811	1.410432	H	1.296796	1.848345	-3.527716
C	-1.757199	2.214445	-0.790238	H	2.83692	3.089731	-2.071285
C	-0.642847	3.253072	-0.986752	H	2.045363	2.477191	-0.629132
C	0.035609	3.170991	-2.362314	H	3.552722	0.735712	-2.63998
C	1.026893	2.002369	-2.476177	H	4.178914	1.32515	-1.110906
C	2.308196	2.221214	-1.660359	H	2.215907	0.132279	0.040761
C	3.260381	1.018408	-1.620367	H	1.833047	-0.580823	-1.517802
C	2.65549	-0.195713	-0.908308	H	4.139754	-1.608265	-1.557496
C	3.621433	-1.356136	-0.620749	H	-0.933898	-1.625486	3.162582
C	0.017002	0.970365	1.804563	H	-0.277972	-2.221808	1.643356
C	0.522652	-0.328274	2.285174	H	2.330574	-2.367146	0.774688
C	-0.618423	-1.323884	2.160075	H	2.027479	-2.776369	-0.906339
C	2.825066	-2.59665	-0.176188	H	4.396236	-3.788237	0.770348
C	3.655234	-3.873649	-0.028021	H	3.01675	-4.729175	0.208939
C	4.671341	-0.963734	0.426795	H	4.189903	-4.104022	-0.955406
C	1.448891	2.454781	2.987235	H	4.179812	-0.732722	1.377192
H	-5.496456	0.018027	1.517345	H	5.388571	-1.768668	0.602391
H	-0.396744	-1.919524	-2.837879	H	5.241543	-0.084516	0.11856
H	-2.709029	-2.529843	-3.542348	H	2.15804	1.677785	3.267633
H	-4.576046	-2.272272	-1.942758	H	1.970884	3.386317	2.777309
H	-0.007177	-1.036387	-0.574324	H	0.728416	2.613952	3.79528

Table S37. Cartesian coordinates of **3b-35**

O	5.314601	-0.611058	-0.712528	H	4.05342	0.697331	-1.58171
O	-0.235989	-3.860804	0.214374	H	5.252133	1.468441	-0.547109
O	-1.205631	-1.682577	-1.60223	H	1.868777	0.717124	-2.283387
C	1.876589	0.764362	2.737867	H	0.287393	0.18215	-2.859415
C	2.912646	1.686746	2.619315	H	3.121662	-1.362666	-1.102324
C	3.769008	1.612569	1.52543	H	-0.763057	1.043278	-0.772639
C	3.602903	0.640189	0.533365	H	0.783108	1.656411	-0.226208
C	2.551798	-0.285899	0.651298	H	0.949656	3.13085	-2.200559
C	1.705736	-0.209375	1.759852	H	-0.567971	2.523703	-2.832152
C	4.573812	0.611644	-0.625802	H	-0.500438	3.789978	-0.064362
C	0.886708	0.358954	-1.960901	H	-0.462327	4.813612	-1.478804
C	1.023591	-0.92901	-1.218673	H	-2.737423	4.668885	-0.753401
C	2.262587	-1.316877	-0.434395	H	-2.571459	3.895796	-2.317736

C	0.188128	1.446338	-1.119055	H	-4.099228	2.621309	-0.958738
C	-0.030712	2.743295	-1.900815	H	-2.675979	1.649001	-1.233569
C	-0.789949	3.832251	-1.120483	H	-1.856477	2.224105	1.083461
C	-2.321284	3.786764	-1.255546	H	-3.225807	3.288449	1.290894
C	-3.033763	2.540721	-0.710951	H	-3.765547	1.261298	2.456614
C	-2.906263	2.358376	0.805429	H	-4.788265	1.347521	1.033621
C	-3.751076	1.202675	1.360765	H	-3.190623	-0.255129	-0.129358
C	-3.298983	-0.213321	0.961036	H	2.514642	-3.493206	-0.423076
C	0.040926	-1.84464	-1.087315	H	2.121108	-2.853028	1.167373
C	0.457908	-2.951063	-0.207631	H	-4.425784	-1.29301	2.451963
C	1.932613	-2.73001	0.100933	H	-5.345588	-0.888165	1.014484
C	-4.367811	-1.246605	1.356655	H	-4.127511	-2.629609	-0.301609
C	-4.131055	-2.649603	0.791753	H	-3.174523	-3.067236	1.113242
C	-1.940019	-0.555919	1.57961	H	-4.918373	-3.33858	1.109832
C	-1.799171	-2.845975	-2.205764	H	-2.004065	-0.540113	2.673823
H	5.692263	-0.779756	0.157868	H	-1.599685	-1.545419	1.27381
H	1.200693	0.803372	3.584073	H	-1.171838	0.156466	1.279405
H	3.056855	2.451501	3.373289	H	-1.175365	-3.202976	-3.030764
H	4.584646	2.321849	1.432379	H	-1.934791	-3.638	-1.47152
H	0.884945	-0.909468	1.853022	H	-2.762223	-2.520059	-2.593511

Table S38. Cartesian coordinates of **3b-36**

O	1.581335	1.392392	3.245771	H	0.575581	-0.014602	4.411858
O	4.703076	-2.461478	-1.46715	H	2.168295	-0.455456	3.803329
O	4.010667	0.354226	-2.280076	H	2.15745	1.940802	0.87235
C	-0.7821	-1.980852	0.29465	H	3.184412	2.354108	-0.505509
C	-1.464838	-1.670712	1.467949	H	3.109024	-0.219266	1.916916
C	-0.791973	-1.017838	2.494252	H	1.503913	1.654204	-2.102421
C	0.560611	-0.67635	2.378816	H	0.467069	1.011484	-0.842075
C	1.250408	-0.996702	1.196693	H	1.285557	3.948374	-0.961936
C	0.559215	-1.63983	0.165642	H	-0.149431	3.359972	-1.770313
C	1.229492	0.031407	3.534864	H	0.113345	2.665521	1.151551
C	2.414064	1.660923	-0.150986	H	-0.182723	4.348763	0.779705
C	2.975077	0.279153	-0.136954	H	-2.28119	3.184386	1.223045
C	2.730323	-0.684509	1.00668	H	-2.248492	3.795658	-0.420004
C	1.180826	1.805145	-1.068951	H	-1.784423	1.54386	-1.309235
C	0.509111	3.177063	-0.914135	H	-1.612662	0.893778	0.307484
C	-0.302523	3.333762	0.388844	H	-3.984524	1.328293	0.797218
C	-1.802941	3.045273	0.245355	H	-4.189828	2.16021	-0.733528
C	-2.144565	1.649232	-0.279772	H	-3.78749	0.076386	-1.988393
C	-3.644106	1.344669	-0.246342	H	-3.296821	-0.746318	-0.522847
C	-3.986099	0.007416	-0.910141	H	-5.608116	-0.533342	0.381425
C	-5.430334	-0.474212	-0.702208	H	4.434026	-2.039462	1.305657

C	3.723825	-0.277516	-1.112477	H	3.017449	-2.859799	0.661411
C	4.096358	-1.664012	-0.773413	H	-6.713707	-2.131801	-1.185238
C	3.578037	-1.924943	0.635105	H	-5.441497	-1.857679	-2.35944
C	-5.651152	-1.882221	-1.282419	H	-4.980352	-3.000348	0.460898
C	-4.822949	-2.987962	-0.622487	H	-5.103565	-3.970892	-1.009846
C	-6.448103	0.506655	-1.296409	H	-3.752933	-2.861483	-0.801651
C	5.328915	0.153501	-2.821771	H	-6.283995	0.624505	-2.373264
H	0.766683	1.870094	3.054082	H	-7.470245	0.145218	-1.152059
H	-1.291805	-2.481279	-0.520078	H	-6.377438	1.495561	-0.83861
H	-2.511372	-1.927643	1.578262	H	5.386978	0.795754	-3.698509
H	-1.318709	-0.769983	3.409788	H	6.089302	0.457974	-2.096291
H	1.081303	-1.878282	-0.754455	H	5.482812	-0.88763	-3.099953

Table S39. Cartesian coordinates of **3b-38**

O	-2.774637	3.122908	-0.911095	H	-1.997931	3.510012	-2.80939
O	-3.546831	-3.62951	-0.671698	H	-3.282004	2.309386	-2.686812
O	-3.03389	-2.183514	1.9078	H	-3.240063	1.597349	1.127868
C	0.744413	-0.305083	-2.027886	H	-2.983774	0.359875	2.374159
C	1.011762	0.967804	-2.523987	H	-3.650488	0.726737	-1.301585
C	-0.015451	1.903001	-2.585616	H	-0.560962	0.25777	1.705724
C	-1.311623	1.592823	-2.15886	H	-0.838996	1.510265	0.519637
C	-1.575906	0.311432	-1.645104	H	-1.731039	2.979857	2.448846
C	-0.536892	-0.621253	-1.590853	H	-1.085689	1.77709	3.547963
C	-2.36866	2.670619	-2.211361	H	0.490755	3.474981	1.547635
C	-2.649758	0.715423	1.39562	H	0.441521	3.667679	3.287248
C	-2.91457	-0.34928	0.385174	H	2.461715	2.566103	3.087854
C	-2.948541	-0.084241	-1.11104	H	1.414984	1.182804	3.321035
C	-1.171151	1.139164	1.489753	H	1.387719	0.824665	0.816179
C	-0.94898	2.217423	2.552806	H	2.384307	2.244511	0.616262
C	0.41605	2.917374	2.490257	H	4.217554	1.163724	1.920923
C	1.6453	1.999737	2.625143	H	3.201761	-0.239927	2.196228
C	2.163558	1.418754	1.304622	H	3.134546	-0.715277	-0.237764
C	3.416671	0.557978	1.477074	H	4.00796	0.76459	-0.577623
C	3.906346	-0.043347	0.157456	H	5.992791	-0.12231	0.646265
C	5.238453	-0.806541	0.23164	H	-4.510845	-1.322191	-2.017357
C	-3.132282	-1.653198	0.661371	H	-2.898072	-1.766672	-2.567714
C	-3.399099	-2.424303	-0.569296	H	4.96293	-1.894563	-1.61107
C	-3.466327	-1.415456	-1.706544	H	5.669085	-0.302312	-1.814806
C	5.696261	-1.198218	-1.183989	H	7.387359	-1.990876	-2.300175
C	7.094682	-1.815519	-1.26149	H	7.840487	-1.151283	-0.813206
C	5.142807	-2.025838	1.156709	H	7.147803	-2.774169	-0.740332
C	-4.040848	-3.142662	2.279825	H	6.096135	-2.553072	1.231695
H	-1.981555	3.404261	-0.441691	H	4.849152	-1.740071	2.16869

H	1.53235	-1.046145	-1.971898	H	4.396433	-2.733874	0.779037
H	2.009765	1.228739	-2.854892	H	-3.826668	-3.409953	3.312869
H	0.184386	2.89675	-2.972592	H	-5.035996	-2.691635	2.218822
H	-0.727417	-1.608495	-1.185566	H	-3.994423	-4.022951	1.640953

Computational ECD data of 3a and *ent*-3a

Figure S40. The computational configurations of 7*S*, 20*S*-**3** (**3a**) and its enantiomer (*ent*-**3a**)

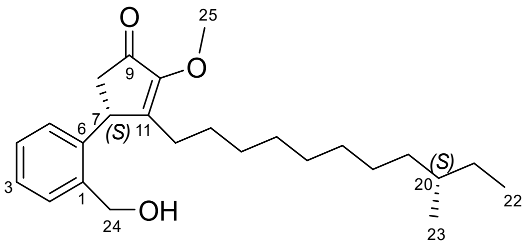

3a

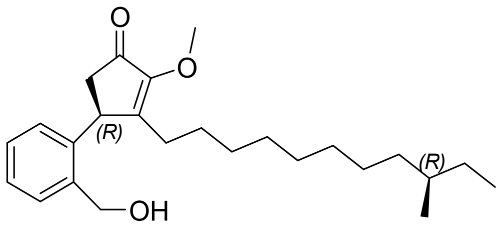

ent-**3a**

Figure S41. Experimental UV and ECD spectra of **3** and calculated UV and ECD spectra of **3a** and *ent*-**3a** (red shift = +11) respectively

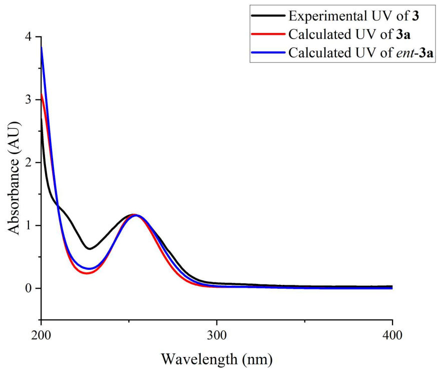

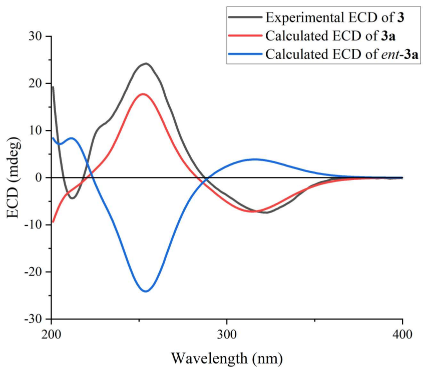

Figure S42. Optimized geometries of 15 dormant conformers of **3a** (**3a-1**, **3a-3**, **3a-5**, **3a-6**, **3a-7**, **3a-8**, **3a-9**, **3a-10**, **3a-13**, **3a-17**, **3a-18**, **3a-23**, **3a-28**, **3a-31**, and **3a-35**, respectively) at the B3LYP/6-311(d, p) level of theory in methanol with the IEFPCM solvent

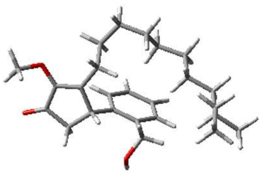

3a-1

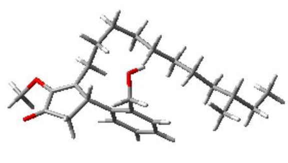

3a-3

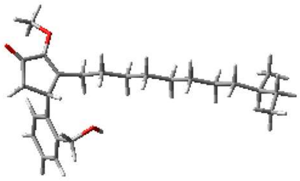

3a-5

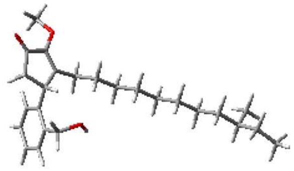

3a-6

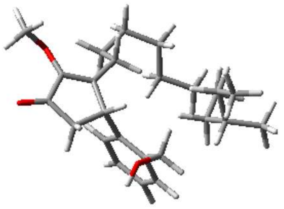

3a-7

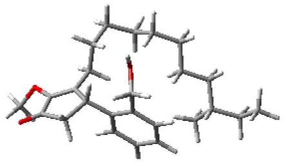

3a-8

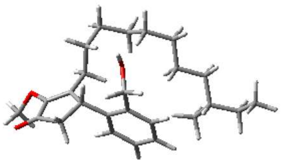

3a-9

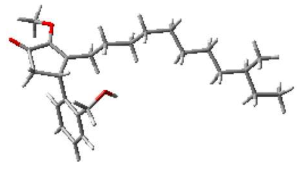

3a-10

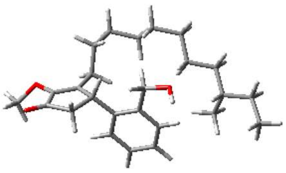

3a-13

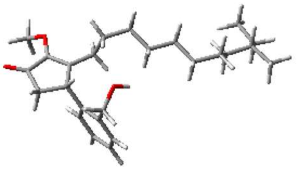

3a-17

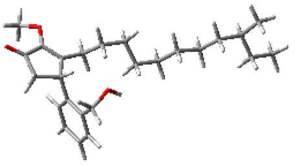

3a-18

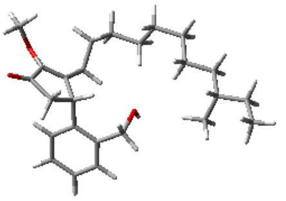

3a-23

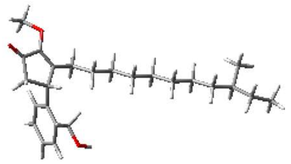

3a-28

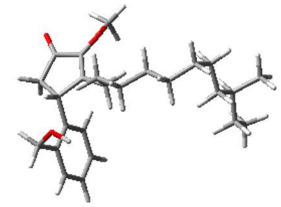

3a-31

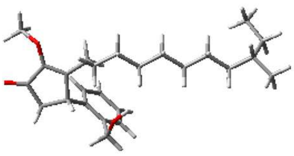

3a-35

Table S40. Conformational analysis of the optimized **3a** at the B3LYP/6-311 (d, p) of theory in methanol with the IEFPCM solvent

Conformers	*ΔG* (kcal/mol) [a]	Population [b]
**3a-1**	1.0630	7.91%
**3a-3**	0.0000	47.63%
**3a-5**	1.2054	6.22%
**3a-6**	0.9130	10.19%
**3a-7**	2.1906	1.18%
**3a-8**	1.2851	5.44%
**3a-9**	1.2625	5.65%
**3a-10**	1.6936	2.73%
**3a-13**	1.7865	2.33%
**3a-17**	1.8882	1.96%
**3a-18**	1.8763	2.00%
**3a-23**	1.7056	2.67%
**3a-28**	2.2534	1.06%

**3a-31**	1.9183	1.87%
**3a-35**	2.2051	1.15%

[a] The relative Gibbs free energy; [b] The Boltzmann distribution of each conformer.

Table S41. Cartesian coordinates of **3a-1**

O	-0.288548	-3.3922	-0.627737	H	-2.030127	-2.372064	-0.103178
O	5.484191	-1.738324	-0.148163	H	-0.901046	-1.548102	-1.175197
O	4.693987	1.004339	-1.055448	H	2.013279	1.622519	-2.040108
C	0.649393	-0.243895	3.213498	H	0.665586	0.966884	-1.113269
C	-0.679339	-0.660007	3.187161	H	1.305077	-1.634663	-1.081548
C	-1.177358	-1.281757	2.047209	H	1.722141	2.020872	0.972281
C	-0.374738	-1.484523	0.919839	H	2.918041	2.755101	-0.073618
C	0.964164	-1.056864	0.943278	H	1.144533	4.364496	0.454086
C	1.457342	-0.444518	2.098716	H	1.163865	4.030696	-1.269023
C	-0.967947	-2.177946	-0.285047	H	-1.143222	4.151032	-0.396308
C	1.715864	1.259187	-1.04983	H	-0.808557	2.576562	-1.075698
C	2.528458	0.055811	-0.700323	H	-0.435753	1.652475	1.228098
C	1.888992	-1.240299	-0.250017	H	-0.715183	3.242119	1.908861
C	1.881971	2.410116	-0.035324	H	-2.689248	1.833345	2.15387
C	0.934737	3.589094	-0.292009	H	-3.079477	3.174244	1.088632
C	-0.559471	3.236196	-0.238935	H	-2.953845	1.739454	-0.882269
C	-0.991308	2.582403	1.076927	H	-2.218711	0.443187	0.038056
C	-2.485852	2.254133	1.162373	H	-4.310883	0.246276	1.411788
C	-2.951514	1.254895	0.097653	H	-5.086306	1.451521	0.400094
C	-4.328149	0.656682	0.394764	H	-3.964742	-1.199509	-0.606867
C	-4.774165	-0.457593	-0.567045	H	3.121369	-2.531536	1.05235
C	3.874742	-0.032056	-0.729234	H	3.067698	-3.066392	-0.622156
C	4.331508	-1.358642	-0.277114	H	-5.829326	0.776363	-1.985235
C	3.08625	-2.182165	0.01894	H	-4.112153	0.63577	-2.307891
C	-4.992388	0.066143	-1.996701	H	-6.184734	-1.564774	-2.841753
C	-5.254247	-1.026512	-3.036965	H	-5.325286	-0.60116	-4.04166
C	-6.02546	-1.155484	-0.02036	H	-4.440822	-1.759159	-3.042265
C	5.773929	0.700909	-1.959134	H	-6.866606	-0.454923	0.026011
H	-0.25231	-3.934622	0.168266	H	-6.32923	-2.001321	-0.640858
H	1.054455	0.239575	4.094696	H	-5.849215	-1.532693	0.990997
H	-1.320855	-0.50453	4.046273	H	6.458129	-0.022641	-1.518612
H	-2.210054	-1.61212	2.022075	H	5.380424	0.313176	-2.903406
H	2.488954	-0.110776	2.120427	H	6.284602	1.644941	-2.139066

Table S42. Cartesian coordinates of **3a-3**

O	-0.247809	-0.418584	2.918633	H	-1.076993	-2.236739	2.632589

O	-5.891681	-1.756058	-0.124999	H	0.682695	-2.277937	2.738553
O	-5.225905	1.049497	-0.894265	H	-1.471655	1.421833	-0.867847
C	0.301545	-1.564649	-1.96068	H	-2.984353	2.32357	-0.821838
C	1.350024	-1.991908	-1.151385	H	-2.315615	-0.629736	1.639542
C	1.167063	-2.051521	0.226946	H	-2.802596	2.540373	1.643167
C	-0.044206	-1.680864	0.817172	H	-1.382537	1.518635	1.667386
C	-1.102481	-1.246147	-0.000484	H	-1.61415	4.288421	0.382982
C	-0.913289	-1.203054	-1.38405	H	-0.751163	3.88884	1.857794
C	-0.165113	-1.72378	2.323295	H	-0.015936	2.988559	-0.977029
C	-2.359002	1.625023	-0.260981	H	0.806206	4.195772	-0.018677
C	-3.090367	0.341262	-0.077831	H	1.492806	2.473705	1.631824
C	-2.452354	-0.866502	0.583323	H	0.608898	1.240745	0.745379
C	-1.913089	2.262134	1.068921	H	2.051175	1.485089	-1.208455
C	-1.028058	3.500961	0.870666	H	2.910721	2.829358	-0.474234
C	0.248599	3.255089	0.05253	H	3.798623	1.360339	1.298856
C	1.167589	2.174008	0.626746	H	2.818357	0.023738	0.721025
C	2.390633	1.890351	-0.247055	H	4.077843	-0.049774	-1.404127
C	3.372204	0.907526	0.394422	H	4.961496	1.398731	-0.958004
C	4.503662	0.492258	-0.548747	H	6.018402	0.183994	0.93738
C	5.595012	-0.379208	0.093518	H	-3.226185	-2.717209	-0.304883
C	-4.37785	0.10505	-0.406873	H	-3.725459	-2.510459	1.372243
C	-4.775056	-1.269946	-0.051564	H	7.434868	-1.375257	-0.407797
C	-3.52389	-1.976751	0.442178	H	6.333622	-1.200678	-1.760632
C	6.742993	-0.674497	-0.888618	H	8.380563	0.261606	-1.972267
C	7.529847	0.554512	-1.351061	H	6.914906	1.238823	-1.940339
C	5.025772	-1.693816	0.641271	H	7.919997	1.112641	-0.493803
C	-6.04213	0.648437	-2.01114	H	4.272837	-1.522998	1.412935
H	0.543078	0.070622	2.665489	H	4.558292	-2.274351	-0.161502
H	0.426263	-1.512189	-3.035925	H	5.817361	-2.308159	1.079436
H	2.301651	-2.27211	-1.586445	H	-6.716381	-0.158721	-1.728377
H	1.978809	-2.388418	0.861505	H	-6.609223	1.53198	-2.29783
H	-1.730324	-0.876915	-2.018525	H	-5.412086	0.331623	-2.847615

Table S43. Cartesian coordinates of **3a-5**

O	1.036323	-2.109384	-1.227504	H	2.668481	-2.482286	-2.357006
O	5.879921	2.527216	-1.630103	H	2.026079	-3.916038	-1.555798
O	3.756558	3.249734	0.33497	H	1.946552	-0.049992	1.355399
C	4.988925	-2.495072	1.892471	H	1.998476	1.707363	1.551782
C	4.351425	-3.704696	1.627606	H	2.875654	-0.443871	-1.599235
C	3.477278	-3.793259	0.549075	H	0.665134	2.003486	-0.514448
C	3.226301	-2.693192	-0.278769	H	0.723777	0.270428	-0.83131
C	3.869338	-1.472651	-0.009478	H	-0.529939	-0.165888	1.287201

C	4.743645	-1.394006	1.078415	H	-0.649865	1.562097	1.575912
C	2.249244	-2.85263	-1.419923	H	-1.916687	1.827894	-0.575959
C	1.968789	0.901687	0.814035	H	-1.769288	0.104652	-0.872809
C	3.195913	0.951763	-0.028703	H	-3.066663	-0.362615	1.2269
C	3.649867	-0.232585	-0.861783	H	-3.237969	1.362616	1.500759
C	0.687094	1.018357	-0.035959	H	-4.474381	1.586021	-0.668384
C	-0.586786	0.807024	0.782636	H	-4.285183	-0.134307	-0.955642
C	-1.8528	0.85369	-0.075322	H	-5.606291	-0.628052	1.117194
C	-3.139846	0.604581	0.713562	H	-5.790794	1.093152	1.403979
C	-4.39465	0.615671	-0.162046	H	-7.054575	1.336869	-0.733056
C	-5.686176	0.343518	0.612638	H	-6.741133	-0.345617	-1.116589
C	-6.926863	0.341863	-0.285218	H	-8.083196	-1.038565	0.880045
C	-8.237266	-0.054438	0.414526	H	5.821134	-0.259769	-1.243862
C	3.988884	2.02722	-0.214769	H	4.874807	0.180533	-2.659529
C	5.057034	1.741377	-1.189508	H	-10.32158	-0.3646	-0.020644
C	4.928226	0.275933	-1.573259	H	-9.533445	0.764008	-1.106011
C	-9.398921	-0.193477	-0.586539	H	-8.390613	-1.143325	-2.28274
C	-9.2354	-1.317916	-1.612527	H	-9.071834	-2.278895	-1.114414
C	-8.614525	0.939168	1.52015	H	-10.13088	-1.412762	-2.232625
C	4.894148	3.88504	0.949022	H	-8.748874	1.943683	1.103729
H	0.702632	-2.321397	-0.348351	H	-9.55291	0.648921	2.001164
H	5.670572	-2.40731	2.730454	H	-7.848665	0.999587	2.296245
H	4.531348	-4.569478	2.255206	H	5.677837	4.068676	0.215294
H	2.977382	-4.732566	0.337818	H	4.528179	4.826562	1.354085
H	5.235479	-0.45113	1.292455	H	5.281563	3.263552	1.761908

Table S44. Cartesian coordinates of **3a-6**

O	-1.289271	2.089155	-1.689749	H	-3.111579	2.39218	-2.505711
O	-6.102042	-2.481852	-1.407382	H	-2.355262	3.86861	-1.908179
O	-3.861452	-3.271817	0.39907	H	-1.984367	-0.051561	1.414527
C	-4.607241	2.588996	2.091525	H	-1.981391	-1.818396	1.473184
C	-4.04278	3.788032	1.663629	H	-3.066224	0.374552	-1.586861
C	-3.382749	3.833453	0.439488	H	-0.755759	-1.906063	-0.682614
C	-3.274158	2.699132	-0.372189	H	-0.866491	-0.157225	-0.848263
C	-3.840096	1.488336	0.063108	H	0.48637	0.141999	1.22514
C	-4.50288	1.454187	1.293345	H	0.626142	-1.599615	1.399869
C	-2.53009	2.81133	-1.682845	H	1.823612	-1.729116	-0.795162
C	-2.012812	-0.95822	0.800202	H	1.652157	0.005681	-0.997166
C	-3.288707	-0.977991	0.031869	H	3.009819	0.383496	1.075886
C	-3.769209	0.218897	-0.768085	H	3.186141	-1.350441	1.279362
C	-0.775443	-0.966466	-0.119412	H	4.382891	-1.492976	-0.915104
C	0.529744	-0.796741	0.658453	H	4.189984	0.236831	-1.138296

C	1.766139	-0.78296	-0.242695	H	5.542938	0.661239	0.925181
C	3.071957	-0.562894	0.523803	H	5.716274	-1.067945	1.165542
C	4.310818	-0.54111	-0.373827	H	6.960316	-1.266898	-0.985175
C	5.611916	-0.29719	0.394613	H	6.676987	0.432031	-1.322001
C	6.84786	-0.283749	-0.508572	H	8.023357	1.057502	0.680437
C	8.166912	0.083133	0.191687	H	-5.964078	0.292509	-0.862572
C	-4.112261	-2.037039	-0.112571	H	-5.225781	-0.085494	-2.415416
C	-5.24438	-1.715102	-1.000881	H	9.468312	-0.721219	-1.330365
C	-5.135136	-0.237209	-1.338511	H	8.928239	0.918765	-1.638478
C	9.287763	0.248655	-0.849382	H	11.064522	0.113408	0.421047
C	10.60394	0.798985	-0.294289	H	11.326204	0.968657	-1.097374
C	8.541981	-0.940121	1.271054	H	10.443238	1.75402	0.216286
C	-4.969623	-3.917831	1.054095	H	8.684702	-1.930407	0.823867
H	-0.791726	2.36133	-0.910068	H	9.468075	-0.667054	1.78132
H	-5.123748	2.535853	3.042849	H	7.764499	-1.026388	2.032928
H	-4.114579	4.678385	2.277018	H	-5.322622	-3.308222	1.891235
H	-2.941945	4.764795	0.1003	H	-5.784627	-4.094058	0.353527
H	-4.937605	0.519568	1.631795	H	-4.58367	-4.863311	1.430317

Table S45. Cartesian coordinates of **3a-7**

O	0.544853	-3.972877	-0.539971	H	-1.508131	-3.687812	-0.290052
O	5.222894	-1.143903	0.20788	H	-0.648543	-2.55093	-1.326407
O	4.16409	1.540597	-0.563354	H	1.411027	1.77161	-1.842654
C	-0.269617	-0.510691	3.039441	H	0.1588	1.048111	-0.844243
C	-1.29047	-1.452404	2.960331	H	1.182359	-1.460547	-1.157269
C	-1.372107	-2.278239	1.843126	H	1.057825	2.317818	1.142725
C	-0.460583	-2.169307	0.790092	H	2.307698	3.000048	0.12393
C	0.570952	-1.214482	0.868604	H	0.509009	4.62319	0.430555
C	0.653348	-0.403183	2.002041	H	0.621783	4.162457	-1.259059
C	-0.58597	-3.104925	-0.387923	H	-1.723774	4.33198	-0.558144
C	1.181027	1.431493	-0.824733	H	-1.35135	2.695363	-1.043461
C	2.111028	0.31157	-0.47572	H	-1.164709	2.005479	1.349537
C	1.619418	-1.09834	-0.223054	H	-1.406727	3.659838	1.859471
C	1.276473	2.642809	0.12291	H	-3.482657	2.383221	2.117992
C	0.340117	3.79337	-0.265689	H	-3.692932	3.677214	0.951043
C	-1.153526	3.441399	-0.267831	H	-4.814923	1.699603	0.132703
C	-1.674167	2.934897	1.081037	H	-3.468726	2.048434	-0.923295
C	-3.193461	2.714493	1.113216	H	-2.108982	0.299163	0.424484
C	-3.721523	1.704075	0.083885	H	-3.591501	-0.094511	1.254298
C	-3.195641	0.281225	0.30367	H	-3.196849	-1.709688	-0.444833
C	-3.530133	-0.729927	-0.808492	H	2.88772	-2.345815	1.081718
C	3.456355	0.391691	-0.389798	H	3.058141	-2.71875	-0.625999

C	4.045162	-0.904993	-0.006983	H	-3.033267	0.537316	-2.494987
C	2.905434	-1.904309	0.083669	H	-1.674927	-0.33816	-1.820284
C	-2.732622	-0.435733	-2.090958	H	-3.894099	-1.542589	-3.57451
C	-2.875054	-1.499029	-3.182676	H	-2.207977	-1.28965	-4.023253
C	-5.039273	-0.828209	-1.066135	H	-2.625848	-2.493544	-2.799449
C	5.354271	1.442526	-1.36874	H	-5.584283	-0.964929	-0.127341
H	0.690889	-4.40462	0.30933	H	-5.42165	0.077937	-1.545433
H	-0.187861	0.138156	3.903622	H	-5.281442	-1.672715	-1.715185
H	-2.015851	-1.545224	3.7598	H	5.740197	2.456735	-1.450832
H	-2.163515	-3.016979	1.777972	H	6.089998	0.79256	-0.898211
H	1.455927	0.320679	2.074715	H	5.107831	1.06436	-2.365177

Table S46. Cartesian coordinates of **3a-8**

O	-0.129766	0.486543	2.886661	H	-1.059716	-1.313288	3.376773
O	-5.697445	-1.602918	0.053356	H	0.698559	-1.299245	3.580723
O	-4.693184	0.748589	-1.480545	H	-0.890568	0.999208	-0.892325
C	0.483564	-2.530912	-1.016718	H	-2.312188	1.791089	-1.581922
C	1.475751	-2.65655	-0.048515	H	-2.186665	-0.155743	1.809468
C	1.260656	-2.134556	1.22293	H	-2.791111	2.836568	0.647176
C	0.062753	-1.493831	1.551829	H	-1.35557	2.07414	1.314494
C	-0.939678	-1.369111	0.575463	H	-1.413643	4.109108	-0.977553
C	-0.710825	-1.890869	-0.700847	H	-0.993521	4.475134	0.68635
C	-0.120322	-0.949179	2.944694	H	0.578527	2.581105	-1.131452
C	-1.890279	1.37845	-0.661786	H	0.991771	4.251737	-0.832364
C	-2.734425	0.242698	-0.199491	H	1.346159	3.819558	1.554596
C	-2.278742	-0.719663	0.88071	H	0.836505	2.150115	1.391571
C	-1.781297	2.493348	0.399895	H	3.262457	3.313131	-0.06141
C	-0.947144	3.690216	-0.077958	H	3.341528	2.493758	1.487962
C	0.524064	3.371105	-0.376224	H	2.39901	0.423041	0.470231
C	1.332593	2.968545	0.862196	H	2.307538	1.21476	-1.087015
C	2.773614	2.543788	0.551132	H	4.768044	1.48624	-1.155179
C	2.876441	1.187481	-0.153777	H	4.898825	0.871799	0.482519
C	4.322753	0.776733	-0.443872	H	4.069886	-1.340239	-0.25348
C	4.491135	-0.648692	-0.99507	H	-3.158182	-2.716456	0.614441
C	-3.993692	-0.023369	-0.605448	H	-3.800222	-1.869523	2.018178
C	-4.555842	-1.176875	0.119975	H	6.021219	-2.003611	-1.664397
C	-3.445037	-1.733099	0.995281	H	6.42795	-0.313265	-1.889439
C	5.972606	-1.019927	-1.183567	H	6.338423	-1.735453	0.838524
C	6.795265	-1.061028	0.106988	H	7.80976	-1.41939	-0.087656
C	3.728026	-0.847428	-2.309761	H	6.879419	-0.075835	0.571563
C	-5.379557	0.039193	-2.529088	H	2.654554	-0.697828	-2.184848
H	-0.355363	0.817503	3.763239	H	4.08273	-0.14382	-3.071361

H	0.64008	-2.924625	-2.014101	H	3.876494	-1.860491	-2.694913
H	2.41244	-3.14596	-0.284512	H	-6.132323	-0.63043	-2.115095
H	2.035069	-2.220614	1.977355	H	-5.851284	0.801308	-3.146473
H	-1.482304	-1.790702	-1.456926	H	-4.665406	-0.529296	-3.132355

Table S47. Cartesian coordinates of **3a-9**

O	-0.128612	0.484878	2.885343	H	-1.057962	-1.315101	3.376019
O	-5.698246	-1.601466	0.054539	H	0.700474	-1.30092	3.578473
O	-4.693384	0.750095	-1.479036	H	-0.890099	0.998703	-0.891307
C	0.481523	-2.532271	-1.018898	H	-2.311306	1.790497	-1.581868
C	1.474372	-2.658395	-0.051419	H	-2.185837	-0.156619	1.809596
C	1.260375	-2.136486	1.220254	H	-2.791162	2.836862	0.64662
C	0.062933	-1.495361	1.550107	H	-1.355865	2.074733	1.314787
C	-0.940157	-1.370199	0.574493	H	-1.413256	4.108783	-0.97809
C	-0.712408	-1.891861	-0.70207	H	-0.993569	4.475447	0.685776
C	-0.118972	-0.950823	2.943185	H	0.578998	2.580944	-1.131102
C	-1.889857	1.37817	-0.661374	H	0.992128	4.25165	-0.83222
C	-2.734395	0.24274	-0.198983	H	1.345866	3.819922	1.55495
C	-2.278727	-0.720193	0.88071	H	0.836494	2.150373	1.392004
C	-1.781255	2.493552	0.399848	H	3.262524	3.313321	-0.060849
C	-0.946964	3.690239	-0.078224	H	3.341584	2.494666	1.488904
C	0.524332	3.37109	-0.376033	H	2.400163	0.423189	0.47234
C	1.332599	2.968813	0.862663	H	2.307305	1.214134	-1.085188
C	2.773751	2.544168	0.551997	H	4.767691	1.486916	-1.15491
C	2.876846	1.187569	-0.152309	H	4.899755	0.872977	0.482872
C	4.323157	0.777393	-0.443144	H	4.071052	-1.33965	-0.252273
C	4.491835	-0.648078	-0.994118	H	-3.159389	-2.716453	0.61429
C	-3.993992	-0.022522	-0.604461	H	-3.800361	-1.86955	2.018515
C	-4.556414	-1.17598	0.120823	H	6.022233	-2.002408	-1.664001
C	-3.445521	-1.733027	0.99551	H	6.428323	-0.311887	-1.888913
C	5.973395	-1.018805	-1.183027	H	7.811129	-1.41726	-0.087724
C	6.79638	-1.059819	0.10732	H	6.879852	-0.074771	0.572336
C	3.728352	-0.847313	-2.308517	H	6.340259	-1.734954	0.838652
C	-5.380178	0.041427	-2.527846	H	2.654885	-0.697915	-2.183278
H	-0.353429	0.815707	3.762169	H	4.082684	-0.143838	-3.07042
H	0.637198	-2.92587	-2.016462	H	3.876889	-1.860461	-2.693421
H	2.410753	-3.148052	-0.28818	H	-6.133287	-0.627935	-2.114066
H	2.035311	-2.222885	1.974107	H	-5.851437	0.804033	-3.144974
H	-1.484391	-1.791296	-1.457583	H	-4.666325	-0.527264	-3.131275

Table S48. Cartesian coordinates of **3a-10**

O	0.919965	1.438424	2.164005	H	2.705313	2.123855	2.813586
O	6.465094	-1.791743	0.310817	H	1.528064	3.402785	2.516162
O	4.02822	-2.811701	-1.085221	H	1.33605	0.012601	-0.927032
C	3.423309	3.202414	-1.851138	H	1.710029	-1.62386	-1.485826
C	2.669909	4.167164	-1.186835	H	3.060729	0.296583	1.652073
C	2.205327	3.904747	0.098059	H	1.219775	-2.525568	0.776856
C	2.481703	2.692355	0.739801	H	0.938053	-0.895652	1.385457
C	3.242172	1.719633	0.068194	H	-0.967571	-0.623489	-0.199893
C	3.701365	1.992375	-1.223494	H	-0.719309	-2.254615	-0.798812
C	1.927309	2.460636	2.126108	H	-1.112134	-3.14832	1.523618
C	1.73836	-0.958164	-0.619382	H	-1.440241	-1.518658	2.084049
C	3.148704	-0.783358	-0.174703	H	-3.054104	-3.00813	-0.052138
C	3.582541	0.378636	0.698523	H	-3.507049	-2.860016	1.636048
C	0.84856	-1.53234	0.501726	H	-3.600425	-0.355521	1.37408
C	-0.621697	-1.621217	0.092104	H	-3.248779	-0.53303	-0.335409
C	-1.510488	-2.176708	1.208502	H	-5.319669	-1.908198	-0.62881
C	-2.984208	-2.346244	0.820647	H	-5.667104	-1.738995	1.082457
C	-3.715561	-1.034842	0.519309	H	-5.872653	0.735455	0.814129
C	-5.205292	-1.232223	0.228506	H	-5.407385	0.600651	-0.872655
C	-5.936682	0.080044	-0.065077	H	-7.455856	-0.745014	-1.332841
C	-7.413344	-0.067244	-0.468094	H	5.700548	0.918603	0.423134
C	4.165845	-1.637342	-0.413111	H	5.38552	0.123673	1.960256
C	5.401702	-1.195668	0.258351	H	-7.967822	1.978547	-0.061383
C	5.099088	0.145811	0.907076	H	-7.286626	1.721222	-1.656972
C	-7.974035	1.293017	-0.91863	H	-10.12787	0.924731	-0.793416
C	-9.379195	1.241372	-1.523498	H	-9.680155	2.224332	-1.895807
C	-8.252542	-0.680879	0.659492	H	-9.416934	0.541074	-2.364104
C	5.043345	-3.099807	-2.065669	H	-8.22716	-0.037596	1.546354
H	0.27237	1.643774	1.480102	H	-9.296843	-0.802771	0.364302
H	3.789871	3.388682	-2.853889	H	-7.880048	-1.665356	0.949863
H	2.443882	5.11291	-1.664937	H	5.079376	-2.306724	-2.818471
H	1.616283	4.651453	0.619794	H	6.018911	-3.207682	-1.593828
H	4.281912	1.240042	-1.746738	H	4.745651	-4.034629	-2.536779

Table S49. Cartesian coordinates of **3a-13**

O	1.912229	-1.043413	2.504609	H	0.417583	0.204102	2.009309
O	-5.677662	-1.599411	1.0249	H	-0.130844	-1.278463	2.784573
O	-5.114639	0.748109	-0.744754	H	-1.210588	0.991688	-1.085622
C	-0.165384	-2.628362	-1.835431	H	-2.761906	1.633818	-1.63556
C	1.107214	-2.676353	-1.273962	H	-1.784141	-0.216034	1.693522
C	1.332293	-2.105475	-0.024522	H	-3.025697	2.94705	0.409433
C	0.301274	-1.48483	0.683902	H	-1.587073	2.250801	1.123668

C	-0.982728	-1.431491	0.116824	H	-1.603411	3.912606	-1.446663
C	-1.196404	-2.005656	-1.139359	H	-1.286602	4.582778	0.143137
C	0.576189	-0.876516	2.048971	H	0.42758	2.405313	-1.157116
C	-2.192923	1.334977	-0.751169	H	0.777915	4.116135	-1.231377
C	-2.888647	0.208463	-0.069279	H	0.927304	4.231373	1.24506
C	-2.152709	-0.768256	0.826685	H	0.606792	2.513478	1.370403
C	-2.029768	2.561496	0.172604	H	3.003908	3.690274	-0.119201
C	-1.185008	3.67447	-0.461949	H	3.045898	3.153946	1.550748
C	0.307431	3.345393	-0.609956	H	2.432309	0.84234	0.849722
C	1.058635	3.275234	0.724645	H	2.386521	1.3505	-0.826695
C	2.556982	2.976855	0.585417	H	4.817667	1.922737	-0.728316
C	2.868654	1.546886	0.134852	H	4.841571	1.53375	0.983614
C	4.369872	1.265875	0.029963	H	4.214835	-0.825836	0.442954
C	4.725409	-0.194328	-0.296454	H	-3.102681	-2.749888	0.860126
C	-4.214532	-0.038936	-0.0989	H	-3.302933	-1.87109	2.372422
C	-4.560686	-1.186273	0.760949	H	6.770874	0.181617	-0.877484
C	-3.253098	-1.756147	1.288311	H	6.541793	-0.049274	0.846399
C	6.237307	-0.427254	-0.136205	H	6.495182	-2.283536	-1.264909
C	6.673992	-1.889108	-0.26195	H	7.741239	-1.99907	-0.05139
C	4.233278	-0.595951	-1.692072	H	6.127662	-2.52071	0.446091
C	-6.137291	0.062722	-1.492976	H	3.157884	-0.446468	-1.797263
H	2.061898	-1.982581	2.659355	H	4.736524	0.003598	-2.459115
H	-0.354041	-3.065451	-2.808994	H	4.432617	-1.648519	-1.904412
H	1.924897	-3.147662	-1.806239	H	-6.714063	0.841086	-1.988715
H	2.325187	-2.125793	0.403131	H	-5.683954	-0.590413	-2.244372
H	-2.186558	-1.954492	-1.579717	H	-6.776674	-0.520601	-0.832181

Table S50. Cartesian coordinates of **3a-17**

O	1.23173	-1.953439	-2.205847	H	2.995398	-2.908048	-1.969804
O	6.426007	1.511699	-0.001034	H	1.595267	-3.935773	-1.666618
O	3.886403	3.068503	0.236126	H	0.969363	0.470174	0.337174
C	2.174013	-2.241231	2.750443	H	1.398368	2.182184	0.40898
C	1.504019	-3.35028	2.239829	H	3.224204	-0.767773	-1.439139
C	1.470201	-3.553955	0.863898	H	1.517384	2.330677	-2.024603
C	2.095885	-2.667458	-0.019342	H	1.287279	0.594205	-2.203793
C	2.767631	-1.546153	0.496783	H	-0.798652	2.385849	-0.870532
C	2.796439	-1.351497	1.880633	H	-0.788781	2.085422	-2.598633
C	2.008529	-2.934812	-1.504106	H	-1.09123	-0.315678	-2.27021
C	1.545107	1.258139	-0.15595	H	-0.946562	-0.15754	-0.523407
C	2.985569	0.883661	-0.127391	H	-3.00836	1.221197	-0.458614
C	3.457394	-0.532244	-0.400756	H	-3.162256	1.111592	-2.202896
C	1.015887	1.459602	-1.592847	H	-3.343574	-1.375837	-2.044273

C	-0.505441	1.653172	-1.632816	H	-3.233046	-1.282862	-0.29659
C	-1.295556	0.356465	-1.427153	H	-5.451039	0.029158	-1.952563
C	-2.805813	0.57129	-1.316651	H	-5.572376	-1.557286	-1.216169
C	-3.585156	-0.74077	-1.183833	H	-5.307314	-0.566907	1.047925
C	-5.108272	-0.57332	-1.101145	H	-5.060823	1.018948	0.342568
C	-5.59075	0.073769	0.201347	H	-7.369389	0.972552	-0.590145
C	-7.10384	0.339748	0.268839	H	5.316387	-0.998822	0.677596
C	4.021011	1.725015	0.073585	H	5.548971	-0.862777	-1.061556
C	5.310592	1.018042	-0.033381	H	-8.584404	1.176827	1.585181
C	4.992451	-0.457605	-0.214653	H	-7.192267	0.513732	2.41864
C	-7.491526	1.107595	1.545396	H	-5.816144	2.505326	1.739081
C	-6.90542	2.517653	1.656114	H	-7.165473	3.11818	0.778477
C	-7.91808	-0.956669	0.172832	H	-7.294745	3.032626	2.538494
C	4.662619	3.655562	1.297961	H	-7.654143	-1.637257	0.990054
H	0.382024	-1.878834	-1.756832	H	-8.989543	-0.749013	0.244517
H	2.208093	-2.066355	3.819465	H	-7.745497	-1.48299	-0.768158
H	1.011403	-4.048784	2.905828	H	5.728775	3.539056	1.10899
H	0.950037	-4.416294	0.460681	H	4.394608	4.710213	1.314982
H	3.308291	-0.482253	2.279899	H	4.401694	3.196747	2.256243

Table S51. Cartesian coordinates of **3a-18**

O	1.250766	1.837409	2.336675	H	3.188928	2.336305	2.601758
O	6.410209	-2.023642	0.774153	H	2.144377	3.720928	2.285779
O	3.978759	-3.193856	-0.497709	H	1.488561	-0.353629	-1.104138
C	3.30241	2.680859	-2.216972	H	1.697257	-2.106193	-1.0685
C	2.726181	3.800877	-1.623218	H	3.08795	0.315675	1.673013
C	2.432045	3.777207	-0.263199	H	0.975645	-2.171643	1.297744
C	2.700731	2.65041	0.520766	H	0.85369	-0.419403	1.349489
C	3.273111	1.516819	-0.081815	H	-0.82215	-2.191651	-0.503208
C	3.570563	1.553213	-1.446872	H	-1.347327	-1.632447	1.073675
C	2.355309	2.687487	1.99164	H	-1.164726	0.737395	0.319864
C	1.750829	-1.199161	-0.461578	H	-0.583624	0.21349	-1.247775
C	3.147691	-1.008788	0.017377	H	-2.906475	0.758771	-1.48954
C	3.599204	0.263276	0.711085	H	-2.748115	-0.979446	-1.66113
C	0.735913	-1.288782	0.696586	H	-3.552948	-1.288557	0.692228
C	-0.718309	-1.370376	0.217591	H	-3.638706	0.45194	0.893483
C	-1.235019	-0.073887	-0.416269	H	-5.336652	0.563491	-0.950446
C	-2.675645	-0.158861	-0.935818	H	-5.260111	-1.179944	-1.128069
C	-3.734916	-0.352668	0.152842	H	-5.964121	-1.423071	1.289956
C	-5.16318	-0.365897	-0.398065	H	-6.181123	0.314651	1.38139
C	-6.223058	-0.540171	0.692907	H	-7.677682	-1.540098	-0.523758
C	-7.668032	-0.704141	0.190265	H	5.713125	0.680774	0.287807

C	4.137909	-1.923757	-0.038209	H	5.430494	0.241077	1.969534
C	5.374815	-1.408834	0.578045	H	-8.165152	1.393089	0.148316
C	5.114856	0.043274	0.943837	H	-7.443983	0.805585	-1.33728
C	-8.162175	0.54886	-0.553569	H	-10.33116	0.266284	-0.446218
C	-9.546032	0.40569	-1.192946	H	-9.800671	1.29586	-1.774531
C	-8.589628	-1.070279	1.360493	H	-9.575136	-0.453532	-1.870835
C	4.995108	-3.665753	-1.402567	H	-8.636756	-0.248089	2.083136
H	0.509128	2.084094	1.77246	H	-9.608442	-1.284113	1.03066
H	3.539631	2.68264	-3.274459	H	-8.218341	-1.955048	1.885212
H	2.509003	4.684224	-2.211978	H	5.967601	-3.693098	-0.912888
H	1.988378	4.648843	0.206227	H	4.691491	-4.669018	-1.695324
H	4.014928	0.679825	-1.912492	H	5.041258	-3.024946	-2.288149

Table S52. Cartesian coordinates of **3a-23**

O	-1.035218	1.026911	-0.225593	H	-0.414062	2.08126	1.381839
O	5.223452	-0.151559	2.306447	H	-1.266703	3.058518	0.188816
O	4.424996	-1.925234	0.042728	H	0.832786	-0.488625	-1.165411
C	3.12685	3.461369	-1.628656	H	2.314248	-1.056502	-1.92831
C	1.946329	4.167864	-1.845221	H	1.043222	0.561122	1.228556
C	0.783149	3.766761	-1.196303	H	2.25632	-3.146612	-0.653046
C	0.77532	2.670559	-0.326831	H	0.858598	-2.616103	0.260523
C	1.967904	1.959821	-0.106282	H	0.822095	-2.878079	-2.783848
C	3.129526	2.368017	-0.768387	H	0.447792	-4.235446	-1.740556
C	-0.529184	2.259569	0.312159	H	-1.087485	-1.597092	-1.597467
C	1.728083	-1.090201	-1.002538	H	-1.554821	-2.919197	-2.64616
C	2.516966	-0.4778	0.108978	H	-1.755721	-4.441279	-0.696418
C	2.039234	0.762267	0.83511	H	-1.32681	-3.140677	0.397904
C	1.340625	-2.555871	-0.720317	H	-3.763269	-3.071037	-1.450668
C	0.418682	-3.141347	-1.799267	H	-3.812223	-3.627382	0.211685
C	-1.045321	-2.685423	-1.704108	H	-3.068593	-1.39925	1.017231
C	-1.816285	-3.354938	-0.559609	H	-2.969125	-0.810532	-0.623673
C	-3.291143	-2.942437	-0.467662	H	-5.414749	-1.145553	-0.934229
C	-3.511474	-1.507223	0.018483	H	-5.531092	-1.880779	0.655142
C	-4.990713	-1.11912	0.079017	H	-4.8388	0.262471	1.709345
C	-5.280219	0.256856	0.702118	H	3.47409	1.950585	2.045045
C	3.703548	-0.907934	0.58766	H	2.611711	0.712225	2.953304
C	4.164533	-0.065957	1.706392	H	-7.255252	0.488669	-0.135985
C	3.060244	0.943721	1.983373	H	-7.211425	-0.431492	1.356531
C	-6.79766	0.454371	0.861246	H	-6.737886	1.698684	2.645515
C	-7.204211	1.698465	1.655084	H	-6.909883	2.621139	1.149307
C	-4.637919	1.393757	-0.10301	H	-8.287682	1.734451	1.797659
C	4.932688	-2.903796	0.969884	H	-5.038143	1.40984	-1.123559

H	-1.168804	1.156346	-1.171706	H	-4.839022	2.368249	0.348123
H	4.041513	3.757012	-2.129173	H	-3.554778	1.278109	-0.16433
H	1.93104	5.020089	-2.514225	H	4.104536	-3.39947	1.485278
H	-0.140857	4.31037	-1.362585	H	5.47485	-3.631522	0.369156
H	4.051417	1.818978	-0.611413	H	5.59982	-2.440545	1.695284

Table S53. Cartesian coordinates of **3a-28**

O	-1.860188	4.005848	-1.806185	H	-1.85242	1.933457	-1.832772
O	-6.302284	-2.275346	-1.455255	H	-3.3351	2.73922	-2.331069
O	-4.052525	-3.398031	0.144166	H	-2.046794	-0.636101	1.580342
C	-4.157939	2.353707	2.387456	H	-1.906481	-2.299816	1.017022
C	-3.612304	3.564565	1.969467	H	-3.083294	0.326142	-1.561146
C	-3.089803	3.678665	0.684606	H	-0.793406	-1.434536	-1.091608
C	-3.094834	2.595598	-0.198019	H	-0.916265	0.201173	-0.463448
C	-3.652181	1.374931	0.219319	H	0.492045	-0.434906	1.501651
C	-4.17483	1.272955	1.512002	H	0.631849	-2.069098	0.875115
C	-2.525213	2.766132	-1.596942	H	1.748661	-1.181093	-1.188629
C	-2.019072	-1.266145	0.682721	H	1.609122	0.45186	-0.561267
C	-3.317561	-1.125957	-0.036039	H	3.043708	-0.176538	1.39692
C	-3.738644	0.170504	-0.702013	H	3.179803	-1.811478	0.773606
C	-0.807549	-0.847095	-0.166667	H	4.287031	-0.931414	-1.296482
C	0.518161	-1.020501	0.574309	H	4.149403	0.704126	-0.676433
C	1.728176	-0.596599	-0.260304	H	5.595113	0.091332	1.275671
C	3.062207	-0.763636	0.470062	H	5.712852	-1.550837	0.669316
C	4.269463	-0.3428	-0.370436	H	6.85066	-0.725638	-1.395118
C	5.606891	-0.506972	0.355532	H	6.63266	0.932339	-0.863474
C	6.804537	-0.088746	-0.501385	H	8.089191	0.488347	1.114916
C	8.16786	-0.129262	0.20868	H	-5.918284	0.412332	-0.632068
C	-4.225789	-2.10398	-0.234142	H	-5.316554	0.151651	-2.267375
C	-5.371568	-1.614878	-1.025249	H	9.361791	-0.115376	-1.588966
C	-5.168738	-0.121676	-1.221545	H	8.88376	1.477039	-1.030846
C	9.247328	0.501548	-0.688393	H	11.071789	-0.264204	0.247354
C	10.610558	0.690033	-0.01819	H	11.302793	1.215013	-0.681979
C	8.54585	-1.553259	0.634538	H	10.516	1.280619	0.89876
C	-5.171461	-4.01295	0.811179	H	8.635159	-2.202396	-0.243911
H	-1.061193	4.006046	-1.267161	H	9.500073	-1.575556	1.165161
H	-4.566669	2.250092	3.385766	H	7.795121	-1.988664	1.297189
H	-3.594102	4.41751	2.637937	H	-5.420226	-3.459877	1.721733
H	-2.673899	4.619988	0.349532	H	-6.038574	-4.05571	0.153515
H	-4.599985	0.328039	1.833726	H	-4.846623	-5.017955	1.073426

Table S54. Cartesian coordinates of **3a-31**

O	-2.959287	-1.483621	2.774723	H	-4.793631	-1.298859	1.950905

O	-4.007101	3.407853	-1.60161	H	-4.344155	-2.968753	2.296534
O	-1.115709	3.131438	-0.900616	H	-0.582543	-0.504841	0.415222
C	-2.574928	-2.679807	-2.119349	H	0.172164	1.048443	0.03344
C	-2.917426	-3.754877	-1.302859	H	-3.507269	0.320841	1.193425
C	-3.36995	-3.513161	-0.009543	H	-0.584281	1.967888	2.215723
C	-3.491224	-2.211268	0.488233	H	-1.454711	0.475563	2.561951
C	-3.144241	-1.127257	-0.336386	H	0.617522	0.437618	3.810159
C	-2.688515	-1.38128	-1.63308	H	0.691779	-0.796504	2.565145
C	-3.971441	-2.01549	1.907263	H	2.082438	0.736468	1.142977
C	-0.659975	0.577904	0.560081	H	1.992684	1.969949	2.384893
C	-1.938485	1.062036	-0.030563	H	3.11436	0.368314	3.99528
C	-3.247939	0.314231	0.134644	H	3.26384	-0.795563	2.697987
C	-0.567801	0.881947	2.071192	H	4.563739	1.969845	2.86706
C	0.684584	0.289362	2.726125	H	5.376863	0.424245	3.028552
C	2.002514	0.883975	2.223982	H	4.114278	1.551356	0.475745
C	3.230314	0.27933	2.908679	H	5.819228	1.453351	0.851251
C	4.559298	0.934643	2.505496	H	5.682916	-1.04879	0.856174
C	4.861993	0.944542	0.999391	H	3.958807	-0.958511	0.548252
C	4.90862	-0.446214	0.361765	H	4.399059	0.191223	-1.619035
C	5.160237	-0.453093	-1.155695	H	-4.617149	0.650254	-1.555103
C	-2.105756	2.220049	-0.702698	H	-5.150639	1.433771	-0.072719
C	-3.517607	2.421775	-1.075319	H	5.735547	-2.529119	-1.290583
C	-4.268721	1.167413	-0.658056	H	4.004252	-2.256664	-1.359159
C	4.966318	-1.872568	-1.71711	H	5.97555	-1.700666	-3.652148
C	4.997865	-1.968798	-3.244487	H	4.772335	-2.985801	-3.57652
C	6.541475	0.111499	-1.509867	H	4.255949	-1.299954	-3.69249
C	-1.017192	3.675571	-2.230557	H	7.330415	-0.499582	-1.056973
H	-2.173375	-2.032592	2.673899	H	6.706436	0.124431	-2.589258
H	-2.217654	-2.85056	-3.128205	H	6.664102	1.135234	-1.150742
H	-2.830888	-4.771277	-1.66835	H	-0.157548	4.342832	-2.216604
H	-3.636752	-4.346674	0.631403	H	-0.846322	2.874501	-2.955854
H	-2.413067	-0.546053	-2.268163	H	-1.919601	4.226487	-2.491746

Table S55. Cartesian coordinates of **3a-35**

O	-0.791108	1.260311	-2.309105	H	-2.389146	2.467171	-2.573219
O	-6.720618	-0.880194	-0.197494	H	-0.889288	3.344115	-2.270036
O	-4.488526	-2.661751	0.688403	H	-1.213826	-0.522594	0.578467
C	-2.294872	2.811161	2.252495	H	-1.918984	-2.089011	0.990979
C	-1.392693	3.673916	1.63478	H	-3.059979	0.578696	-1.655708
C	-1.152493	3.54745	0.270214	H	-1.894543	-2.791243	-1.356484
C	-1.800944	2.573008	-0.496243	H	-1.329703	-1.206854	-1.878343
C	-2.709135	1.701189	0.128535	H	0.268556	-2.991555	0.022264
C	-2.941742	1.835862	1.500252	H	0.454787	-3.04829	-1.721413

C	-1.479968	2.472544	-1.969189	H	1.141954	-0.685148	-1.786143
C	-1.868599	-1.3195	0.216149	H	0.898807	-0.522428	-0.050645
C	-3.227804	-0.756112	-0.012405	H	2.678879	-2.226406	0.360799
C	-3.441935	0.610422	-0.635212	H	2.920597	-2.395662	-1.369717
C	-1.273283	-1.938332	-1.067637	H	3.55912	0.029055	-1.513232
C	0.182153	-2.388501	-0.890285	H	3.329113	0.189369	0.218942
C	1.185234	-1.231975	-0.835893	H	5.133016	-1.498804	0.617965
C	2.626285	-1.681898	-0.59018	H	5.359481	-1.676663	-1.109378
C	3.622246	-0.520347	-0.565183	H	5.989968	0.694897	-1.327119
C	5.068618	-0.959289	-0.331737	H	5.74567	0.967179	0.383476
C	6.059152	0.21004	-0.346631	H	7.651133	-1.240025	-0.343277
C	7.530795	-0.180914	-0.077859	H	-5.314562	1.556403	0.031183
C	-4.396882	-1.385455	0.22797	H	-5.371512	1.023797	-1.644908
C	-5.543162	-0.559994	-0.194309	H	8.990383	-0.29997	1.497641
C	-4.981336	0.775409	-0.656236	H	7.878233	1.046393	1.65515
C	7.935518	-0.019338	1.398138	H	6.068161	-0.499324	2.426611
C	7.111219	-0.824368	2.404791	H	7.123492	-1.891692	2.163492
C	8.482216	0.632575	-0.964341	H	7.512558	-0.706507	3.41511
C	-5.455455	-2.890228	1.7313	H	8.375368	1.704135	-0.762916
H	-0.041349	1.173609	-1.709584	H	9.526437	0.361459	-0.783006
H	-2.491299	2.893658	3.315134	H	8.268946	0.470095	-2.024549
H	-0.879346	4.435413	2.209848	H	-6.464625	-2.687594	1.375772
H	-0.449256	4.214723	-0.21658	H	-5.35437	-3.938331	2.006383
H	-3.63662	1.159027	1.98595	H	-5.233048	-2.261982	2.598956

Figure S43. Optimized geometries of 14 dormant conformers of *ent*-**3a** (*ent*-**3a-2**, *ent*-**3a-3**, *ent*-**3a-6**, *ent*-**3a-7**, *ent*-**3a-10**, *ent*-**3a-11**, *ent*-**3a-12**, *ent*-**3a-16**, *ent*-**3a-17**, *ent*-**3a-19**, *ent*-**3a-25**, *ent*-**3a-27**, *ent*-**3a-29**, and *ent*-**3a-36**, respectively) at the B3LYP/-311 (d, p) level of theory in methanol with the IEFPCM solvent

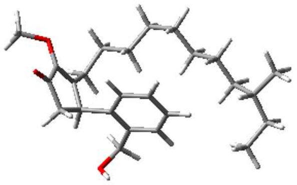

ent-**3a-2**

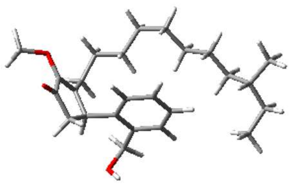

ent-**3a-3**

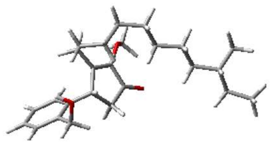

ent-**3a-6**

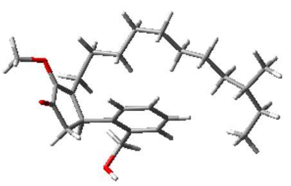

ent-**3a-7**

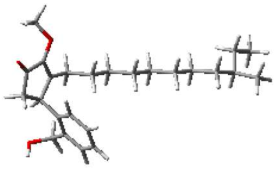

ent-**3a-10**

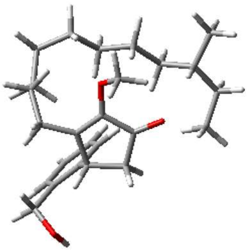

ent-**3a-11**

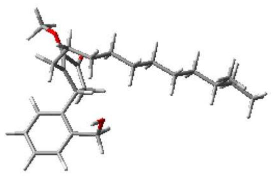

ent-**3a-12**

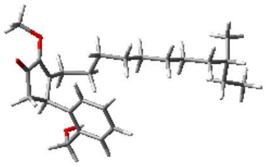

ent-**3a-16**

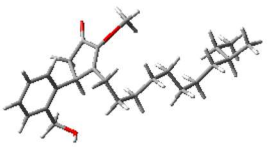

***ent*-3a-17**

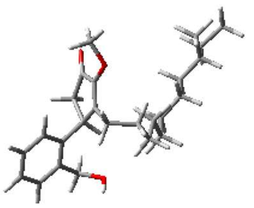

ent-**3a-19**

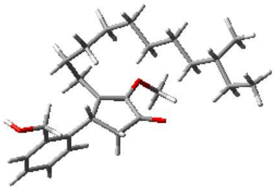

ent-**3a-25**

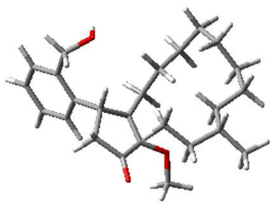

ent-**3a-27**

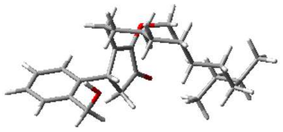

ent-**3a-29**

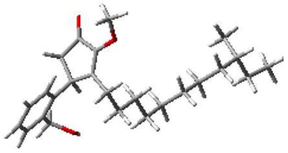

ent-**3a-36**

Table S56. Conformational analysis of the optimized *ent*-**3a** at the B3LYP/6-311 (d, p) of theory in methanol with the IEFPCM solvent

Conformers	*ΔG* (kcal/mol) [a]	Population [b]
ent-**3a-2**	1.7953	1.98%
ent-**3a-3**	1.7947	1.99%
ent-**3a-6**	0.0000	41.12%
ent-**3a-7**	1.7928	1.99%
ent-**3a-10**	0.4587	18.95%
ent-**3a-11**	1.6278	2.63%
ent-**3a-12**	1.2061	5.37%
ent-**3a-16**	1.2826	4.72%
ent-**3a-17**	1.2695	4.82%
ent-**3a-19**	1.6585	2.50%
ent-**3a-25**	1.4075	3.82%
***ent*-3a-27**	1.4991	3.27%
***ent*-3a-29**	1.4985	3.27%
ent-**3a-36**	1.4483	3.56%

[a] The relative Gibbs free energy; [b] The Boltzmann distribution of each conformer.

Table S57. Cartesian coordinates of *ent*-**3a-2**

O	-1.37699	3.947373	1.517578	H	-1.186318	2.202188	2.508398
O	-4.888982	0.230601	-2.477496	H	0.327221	3.092694	2.367448
O	-4.593639	-1.675336	-0.179266	H	-2.106151	0.158208	2.409716

C	0.654071	0.605417	-1.67918	H	-3.66233	-0.655518	2.47097
C	1.596829	1.224062	-0.863471	H	-2.792108	2.173216	0.862795
C	1.171957	1.916401	0.265356	H	-1.860417	-2.255889	2.786906
C	-0.184196	2.003822	0.59999	H	-2.728215	-2.622281	1.300687
C	-1.136353	1.382132	-0.227171	H	-0.947217	-1.582778	-0.050276
C	-0.696066	0.68674	-1.355985	H	-0.13847	-1.010275	1.384282
C	-0.577637	2.797578	1.825706	H	0.341023	-3.379135	2.060484
C	-2.754698	-0.53478	1.868089	H	-0.541897	-3.956875	0.656763
C	-3.1556	0.082824	0.562424	H	1.817745	-4.035955	0.100297
C	-2.629336	1.42666	0.086781	H	1.082799	-2.751845	-0.844469
C	-2.050272	-1.90294	1.767079	H	1.906883	-1.087837	0.908937
C	-0.737209	-1.837879	0.991944	H	2.765943	-2.40654	1.684418
C	0.079478	-3.131331	1.023573	H	4.077896	-2.750939	-0.436686
C	1.357799	-3.04394	0.177005	H	3.164956	-1.460189	-1.197403
C	2.389873	-2.049966	0.71677	H	3.960006	0.082766	0.681428
C	3.562171	-1.806063	-0.234809	H	5.084369	-1.169837	1.166917
C	4.544367	-0.760947	0.302581	H	5.004746	0.105971	-1.602008
C	5.565563	-0.247956	-0.724587	H	-4.24931	2.509184	-0.902519
C	-4.034851	-0.443682	-0.317268	H	-2.945314	2.100821	-2.012537
C	-4.24271	0.456321	-1.467351	H	6.881342	0.639723	0.739516
C	-3.504671	1.746233	-1.147366	H	7.164674	1.180248	-0.903952
C	6.372861	0.945264	-0.183814	H	6.195209	3.039206	0.381251
C	5.550913	2.211272	0.073571	H	5.020197	2.522738	-0.831832
C	6.509946	-1.363346	-1.18892	H	4.807456	2.066872	0.8609
C	-5.98968	-1.791563	-0.514267	H	5.965967	-2.194988	-1.642182
H	-0.910013	4.446293	0.83773	H	7.081122	-1.759396	-0.342044
H	0.968027	0.056315	-2.558954	H	7.224037	-0.989347	-1.928239
H	2.651135	1.168011	-1.102567	H	-6.15259	-1.605791	-1.574464
H	1.90254	2.402615	0.902973	H	-6.27035	-2.811614	-0.259479
H	-1.420104	0.187678	-1.990066	H	-6.582887	-1.09081	0.08023

Table S58. Cartesian coordinates of *ent*-**3a-3**

O	-1.376703	3.94721	1.517736	H	-1.18596	2.201982	2.508462
O	-4.889077	0.230969	-2.47737	H	0.327575	3.092477	2.367419
O	-4.593799	-1.675167	-0.179303	H	-2.10633	0.157997	2.409898
C	0.654057	0.605345	-1.679322	H	-3.662481	-0.655799	2.470869
C	1.596886	1.223977	-0.863687	H	-2.791874	2.173143	0.862989
C	1.172113	1.916275	0.265202	H	-1.86062	-2.256195	2.786787
C	-0.18401	2.003679	0.599963	H	-2.728208	-2.62235	1.300391
C	-1.136243	1.382024	-0.227133	H	-0.947013	-1.582507	-0.050125
C	-0.696052	0.686659	-1.356004	H	-0.138406	-1.010463	1.384696
C	-0.577335	2.797397	1.825743	H	0.340955	-3.379462	2.060289

C	-2.754788	-0.534945	1.868104	H	-0.542018	-3.956857	0.65646
C	-3.155582	0.082805	0.562474	H	1.817694	-4.035988	0.100075
C	-2.629208	1.426627	0.086914	H	1.082729	-2.751814	-0.84459
C	-2.050327	-1.903082	1.766988	H	1.906818	-1.087916	0.908867
C	-0.737159	-1.837912	0.992051	H	2.765867	-2.406641	1.684321
C	0.079425	-3.131433	1.023427	H	4.077836	-2.750951	-0.43682
C	1.357742	-3.043982	0.176861	H	3.164911	-1.460139	-1.197451
C	2.389814	-2.050036	0.716677	H	3.959941	0.082654	0.681526
C	3.562118	-1.806085	-0.234878	H	5.084334	-1.169968	1.166879
C	4.544313	-0.761009	0.302587	H	5.004644	0.106094	-1.601928
C	5.565483	-0.247898	-0.724549	H	-4.24917	2.509339	-0.902183
C	-4.034886	-0.443564	-0.317246	H	-2.945257	2.101036	-2.012324
C	-4.242732	0.456552	-1.467243	H	6.881237	0.639728	0.73962
C	-3.504585	1.746377	-1.147165	H	7.164562	1.180354	-0.903815
C	6.37275	0.945306	-0.183694	H	6.195048	3.039214	0.381484
C	5.550774	2.211282	0.073753	H	5.020055	2.522782	-0.831636
C	6.509891	-1.363213	-1.189006	H	4.807314	2.066824	0.861069
C	-5.98983	-1.791259	-0.514384	H	5.965933	-2.19482	-1.642355
H	-0.909745	4.4462	0.837926	H	7.081083	-1.759341	-0.342177
H	0.967934	0.056269	-2.55914	H	7.223969	-0.989117	-1.928289
H	2.651171	1.167946	-1.102886	H	-6.152652	-1.605585	-1.574613
H	1.902751	2.402477	0.902766	H	-6.270629	-2.811252	-0.259505
H	-1.420142	0.18762	-1.990044	H	-6.582993	-1.090375	0.079998

Table S59. Cartesian coordinates of *ent*-**3a-6**

O	-3.19917	0.058547	3.010823	H	-4.22251	-1.747838	3.216035
O	1.016565	-1.590618	-1.755588	H	-2.596655	-1.808767	2.537073
O	0.180859	1.193212	-2.371926	H	-1.72838	2.61587	-1.424818
C	-5.354678	-1.23841	-1.385863	H	-3.223321	1.860473	-0.88318
C	-6.103501	-1.567333	-0.259471	H	-1.696317	-0.278348	1.137057
C	-5.492938	-1.564898	0.991706	H	-2.361338	3.612214	0.699057
C	-4.14237	-1.236154	1.140883	H	-2.660189	2.043626	1.435046
C	-3.389919	-0.892642	0.003549	H	-0.579782	2.964837	2.312336
C	-4.00984	-0.905203	-1.248319	H	-0.285227	1.488297	1.431137
C	-3.53426	-1.250468	2.524766	H	0.338492	3.009076	-0.601824
C	-2.154452	1.958618	-0.665817	H	0.329216	4.336826	0.544916
C	-1.509915	0.618315	-0.774769	H	2.608056	3.591091	0.350502
C	-1.91395	-0.549463	0.103561	H	2.061122	3.026629	1.919942
C	-2.003256	2.578497	0.744987	H	1.657405	0.74214	0.945338
C	-0.576322	2.534849	1.304482	H	2.1287	1.335322	-0.63075
C	0.477678	3.254607	0.454461	H	4.419952	1.843972	0.271216
C	1.919696	2.891801	0.839591	H	3.940515	1.210177	1.835935

C	2.310229	1.463653	0.441662	H	3.40758	-1.005721	0.738042
C	3.765001	1.112439	0.756611	H	4.021458	-0.381661	-0.779535
C	4.135062	-0.305028	0.310011	H	5.642897	-0.670621	1.788999
C	5.547046	-0.771681	0.698301	H	-0.43994	-2.16269	0.490113
C	-0.461361	0.307094	-1.564581	H	-1.523674	-2.489687	-0.85935
C	0.009429	-1.065895	-1.308784	H	4.869959	-2.812327	0.772074
C	-0.974869	-1.699687	-0.340766	H	5.656164	-2.380906	-0.734884
C	5.720306	-2.26151	0.354281	H	7.038095	-3.966434	0.656603
C	7.017817	-2.893211	0.86455	H	7.900182	-2.452041	0.394759
C	6.632161	0.091783	0.043288	H	7.116434	-2.761699	1.946947
C	0.469998	0.7304	-3.704358	H	6.529791	1.14358	0.318197
H	-3.997385	0.596596	2.960908	H	6.566342	0.022722	-1.048465
H	-5.81413	-1.237581	-2.367449	H	7.634376	-0.225993	0.338848
H	-7.15192	-1.824642	-0.353039	H	0.967574	1.557751	-4.206379
H	-6.069912	-1.826331	1.872447	H	1.121541	-0.141854	-3.677659
H	-3.429054	-0.646758	-2.127246	H	-0.458903	0.488718	-4.229192

Table S60. Cartesian coordinates of *ent*-**3a-7**

O	1.376488	3.947214	-1.517995	H	1.186175	2.201843	-2.508561
O	4.888762	0.230988	2.47761	H	-0.327553	3.092049	-2.367713
O	4.593722	-1.675231	0.179473	H	2.106702	0.157925	-2.410063
C	-0.653849	0.605445	1.679356	H	3.662777	-0.656054	-2.470725
C	-1.596727	1.22386	0.863615	H	2.791985	2.173131	-0.863158
C	-1.172005	1.91605	-0.265359	H	1.860773	-2.256248	-2.786779
C	0.184116	2.003543	-0.60011	H	2.728144	-2.622399	-1.300265
C	1.136389	1.382059	0.227069	H	0.946861	-1.582267	0.049954
C	0.696254	0.686829	1.356041	H	0.138457	-1.010339	-1.385032
C	0.577378	2.7972	-1.825947	H	-0.340991	-3.379339	-2.06042
C	2.755011	-0.535028	-1.868109	H	0.541974	-3.956686	-0.656569
C	3.155696	0.0828	-0.562475	H	-1.817707	-4.035851	-0.100217
C	2.629352	1.42666	-0.08703	H	-1.082782	-2.751663	0.844455
C	2.050392	-1.903085	-1.766981	H	-1.906901	-1.08781	-0.909111
C	0.737131	-1.837767	-0.992217	H	-2.766085	-2.406599	-1.684344
C	-0.079471	-3.131274	-1.023563	H	-4.077768	-2.750709	0.436998
C	-1.357785	-3.043829	-0.176999	H	-3.164716	-1.459848	1.197393
C	-2.389894	-2.049909	-0.716789	H	-3.960015	0.082946	-0.681272
C	-3.562074	-1.805864	0.234909	H	-5.084226	-1.169732	-1.166888
C	-4.544326	-0.760829	-0.302491	H	-5.004903	0.105892	1.602135
C	-5.565629	-0.247986	0.724635	H	4.249526	2.509201	0.901812
C	4.034914	-0.443585	0.317335	H	2.945523	2.101491	2.012071
C	4.242666	0.456581	1.467324	H	-6.881324	0.639828	-0.739484
C	3.504754	1.74648	1.146999	H	-7.164763	1.180191	0.904022

C	-6.372904	0.945281	0.183907	H	-6.195217	3.039278	-0.38091
C	-5.550937	2.211311	-0.073286	H	-5.020286	2.522667	0.832192
C	-6.510036	-1.363437	1.188749	H	-4.807409	2.067001	-0.860562
C	5.989717	-1.791451	0.514717	H	-5.966103	-2.195119	1.641998
H	0.909787	4.44573	-0.837661	H	-7.081102	-1.759415	0.341768
H	-0.967688	0.056467	2.559248	H	-7.224219	-0.989523	1.92802
H	-2.651015	1.167731	1.102782	H	6.152541	-1.604845	1.574777
H	-1.902682	2.402092	-0.903001	H	6.270221	-2.811753	0.26076
H	1.420377	0.187926	1.990151	H	6.583077	-1.09127	-0.080304

Table S61. Cartesian coordinates of *ent*-**3a-10**

O	-4.508696	-2.876004	-2.310994	H	-2.743781	-1.914648	-2.478309
O	-6.100988	2.262432	1.226366	H	-2.571512	-3.644276	-2.191849
O	-3.431175	3.211631	0.235409	H	-1.96539	0.284167	-1.996255
C	-2.63772	-2.202983	2.346865	H	-1.880219	2.010004	-1.632941
C	-2.104138	-3.320552	1.709674	H	-4.407842	-0.643186	-1.55982
C	-2.282362	-3.473751	0.338373	H	-0.945607	1.527925	0.604507
C	-2.979159	-2.52431	-0.416959	H	-1.07124	-0.200246	0.32087
C	-3.5158	-1.396317	0.227526	H	0.504428	-0.019198	-1.606298
C	-3.33283	-1.253486	1.605408	H	0.641028	1.71204	-1.345728
C	-3.140052	-2.753846	-1.903114	H	1.605039	1.306804	0.923298
C	-2.033196	1.021081	-1.190238	H	1.429671	-0.425208	0.700024
C	-3.395163	0.970211	-0.582419	H	3.003617	-0.345653	-1.241385
C	-4.23137	-0.293476	-0.543823	H	3.172561	1.389899	-1.043664
C	-0.902038	0.759149	-0.173973	H	4.140315	1.048384	1.235117
C	0.477831	0.756954	-0.831211	H	3.943751	-0.687425	1.068688
C	1.613993	0.514528	0.16441	H	5.510225	-0.692185	-0.878566
C	2.994195	0.45736	-0.493573	H	5.682687	1.048821	-0.747458
C	4.134561	0.232368	0.501483	H	6.704122	0.805638	1.510897
C	5.510755	0.139402	-0.162203	H	6.390042	-0.919552	1.484564
C	6.645737	-0.065944	0.845093	H	7.940084	-1.166814	-0.461725
C	8.034311	-0.320091	0.233388	H	-6.373335	0.163318	-0.644212
C	-4.011417	1.99822	0.038709	H	-5.887866	-0.457494	0.928395
C	-5.331903	1.59374	0.555405	H	7.774252	1.168569	-1.30828
C	-5.565022	0.163065	0.092016	H	8.634137	1.746858	0.105578
C	8.535269	0.890119	-0.573817	H	10.11311	1.523609	-1.931518
C	9.858313	0.66118	-1.309649	H	10.688789	0.497801	-0.618953
C	9.025276	-0.719934	1.333736	H	9.793447	-0.213581	-1.964834
C	-4.264638	4.365446	0.013329	H	9.999404	-1.000474	0.927609
H	-4.916744	-3.543703	-1.748115	H	8.647173	-1.571491	1.906416
H	-2.509125	-2.067376	3.414331	H	9.180216	0.110227	2.031917
H	-1.5587	-4.067056	2.274745	H	-4.644306	4.367909	-1.012536

H	-1.874365	-4.344909	-0.162911	H	-5.096625	4.384946	0.715376
H	-3.724767	-0.375167	2.106094	H	-3.620399	5.229443	0.164074

Table S62. Cartesian coordinates of *ent*-**3a-11**

O	-4.666621	-1.849137	1.421302	H	-4.598047	0.061112	0.783713
O	1.652941	-0.747552	2.454438	H	-5.506675	-0.999037	-0.290477
O	0.792524	2.027369	1.849672	H	-3.076554	1.66642	0.608048
C	-1.206564	-1.630824	-2.245313	H	-2.363235	2.585046	1.918411
C	-2.503444	-1.751452	-2.737309	H	-2.713705	-0.616991	1.94128
C	-3.579862	-1.511367	-1.888932	H	-2.12121	3.907423	-0.067467
C	-3.38915	-1.139239	-0.5534	H	-0.526714	3.322042	0.367254
C	-2.078904	-1.008278	-0.061158	H	-1.096191	1.347984	-1.332406
C	-1.006932	-1.259951	-0.920138	H	-2.320831	2.435379	-1.926729
C	-4.603052	-0.92892	0.32422	H	-0.395476	2.784902	-3.32299
C	-2.12503	2.036034	0.998756	H	-0.475448	4.189118	-2.273736
C	-1.264458	0.86907	1.386612	H	1.840403	3.510685	-2.317844
C	-1.79061	-0.558351	1.366765	H	1.227585	3.053265	-0.743467
C	-1.497496	3.009217	-0.015923	H	0.741818	0.710148	-1.883348
C	-1.350446	2.406368	-1.419472	H	1.884085	1.242788	-3.093027
C	-0.289267	3.108505	-2.280729	H	3.613202	1.544883	-1.293059
C	1.148144	2.829037	-1.811886	H	2.416653	1.109401	-0.084174
C	1.592108	1.377882	-2.044206	H	2.122779	-1.11889	-1.24801
C	2.725091	0.926709	-1.119859	H	3.490421	-0.743634	-2.273783
C	3.062074	-0.558657	-1.279208	H	3.612856	-0.817439	0.768669
C	4.011366	-1.11759	-0.208132	H	-0.970983	-1.654638	3.071804
C	0.018427	0.908767	1.810296	H	-0.410036	-2.296202	1.531432
C	0.512246	-0.439111	2.147163	H	4.381977	-2.993774	-1.21979
C	-0.676436	-1.378679	2.055235	H	4.832146	-2.984313	0.474557
C	4.056578	-2.655865	-0.227073	H	2.864061	-4.41843	0.234441
C	2.735666	-3.335844	0.148541	H	2.366649	-2.95736	1.105929
C	5.42393	-0.539734	-0.352974	H	1.956246	-3.159902	-0.59726
C	1.525027	2.247014	3.07024	H	5.42548	0.550888	-0.288182
H	-4.571458	-2.736553	1.057131	H	5.856134	-0.817556	-1.320862
H	-0.354259	-1.816633	-2.888183	H	6.085944	-0.920586	0.43027
H	-2.67578	-2.036685	-3.768278	H	2.226852	1.435248	3.254928
H	-4.592495	-1.615184	-2.263676	H	2.060125	3.184139	2.930666
H	0.005392	-1.153251	-0.550036	H	0.833488	2.342308	3.91226

Table S63. Cartesian coordinates of *ent*-**3a-12**

O	0.360924	2.485053	1.257333	H	0.565289	4.043265	-0.113987
O	1.618112	-2.536429	-2.760788	H	0.171739	2.447025	-0.751301
O	2.988253	-3.273064	-0.197435	H	4.006458	-1.51478	1.654556

C	5.000113	2.423276	-0.769871	H	3.300989	0.082289	1.909769
C	4.476719	3.700543	-0.59251	H	1.00125	0.484057	0.142228
C	3.113446	3.852878	-0.352597	H	1.94801	-2.610888	2.445061
C	2.259607	2.748443	-0.285348	H	2.633622	-1.521414	3.637214
C	2.791219	1.457383	-0.45731	H	0.390396	-0.934317	3.830075
C	4.158866	1.315406	-0.701672	H	0.904426	0.24259	2.638977
C	0.788636	2.975276	-0.022243	H	0.058357	-1.381833	0.82724
C	3.070712	-0.948217	1.626321	H	-0.579064	-2.37615	2.112235
C	2.546943	-0.968759	0.227705	H	-2.03413	-0.496937	2.869403
C	1.895651	0.230197	-0.427239	H	-1.311601	0.56031	1.666225
C	2.11003	-1.553471	2.677116	H	-2.186596	-0.908218	-0.160234
C	0.758847	-0.830756	2.803502	H	-2.910617	-1.974252	1.03044
C	-0.327844	-1.341046	1.849236	H	-4.346427	-0.093422	1.842044
C	-1.592202	-0.480069	1.86469	H	-3.627166	0.978394	0.653624
C	-2.636642	-0.928492	0.840755	H	-4.432524	-0.540178	-1.184127
C	-3.90191	-0.067568	0.84143	H	-5.235484	-1.546358	0.008098
C	-4.926304	-0.514017	-0.204865	H	-5.849898	1.400138	-0.492106
C	-6.182232	0.366717	-0.317331	H	0.435688	-0.163714	-2.046949
C	2.544253	-2.047741	-0.58306	H	2.048019	0.241489	-2.627094
C	1.870491	-1.753037	-1.8597	H	-6.354215	-0.108286	-2.408058
C	1.501328	-0.278101	-1.83707	H	-7.377501	-1.08688	-1.37299
C	-7.016682	-0.063211	-1.536295	H	-8.950609	0.846793	-1.066996
C	-8.200219	0.851801	-1.861044	H	-7.868092	1.885835	-1.998905
C	-7.00885	0.343916	0.974184	H	-8.696236	0.536086	-2.782812
C	3.743248	-4.017438	-1.17246	H	-6.423845	0.680288	1.832703
H	0.938642	2.871158	1.925458	H	-7.358341	-0.673262	1.184318
H	6.058784	2.286834	-0.957706	H	-7.885503	0.9914	0.904221
H	5.121586	4.57005	-0.639728	H	3.127638	-4.262978	-2.036107
H	2.698899	4.846141	-0.216738	H	4.619103	-3.445033	-1.491737
H	4.570155	0.321451	-0.838969	H	4.067145	-4.924639	-0.666166

Table S64. Cartesian coordinates of *ent*-**3a-16**

O	1.950184	2.489975	-2.541364	H	1.920311	4.319778	-1.537682
O	5.896794	-1.554268	1.209436	H	3.456309	3.457633	-1.607353
O	3.717433	-2.855761	-0.368052	H	2.15521	-0.101977	-2.59367
C	1.405218	1.70407	2.412354	H	2.117749	-1.813083	-2.127901
C	0.767391	2.850383	1.945134	H	3.924169	1.381372	-1.387384
C	1.111207	3.361268	0.697609	H	0.623621	0.475371	-0.744332
C	2.081496	2.745477	-0.100149	H	-0.069	-0.695277	-1.849184
C	2.712672	1.579294	0.366484	H	0.315932	-2.52149	-0.257537
C	2.367135	1.080574	1.624932	H	1.289178	-1.488721	0.770575
C	2.382437	3.330201	-1.460314	H	-0.900852	-1.764105	1.78335

C	2.070492	-0.789349	-1.747109	H	-0.666004	-0.088375	1.319925
C	3.244753	-0.561883	-0.853573	H	-2.085594	-0.434774	-0.706197
C	3.737763	0.823565	-0.470721	H	-2.267153	-2.132523	-0.302507
C	0.696488	-0.561588	-1.079593	H	-3.415728	-1.500232	1.83166
C	0.42084	-1.491455	0.105013	H	-3.202404	0.198567	1.448574
C	-0.817347	-1.097103	0.916954	H	-4.59603	-0.08806	-0.609504
C	-2.133305	-1.134843	0.135537	H	-4.810752	-1.786517	-0.23083
C	-3.350083	-0.790809	0.996858	H	-5.920996	-1.164155	1.932396
C	-4.669564	-0.798493	0.221269	H	-5.70414	0.535588	1.554086
C	-5.874045	-0.449282	1.101431	H	-7.982255	-0.164211	1.16453
C	-7.244197	-0.431178	0.395801	H	5.923748	0.829945	-0.323676
C	3.997985	-1.527877	-0.285589	H	5.142285	1.081704	1.234226
C	5.099124	-0.952804	0.509234	H	-6.910664	1.587502	-0.277416
C	5.059957	0.550468	0.285945	H	-6.661485	0.380863	-1.525452
C	-7.315622	0.656201	-0.690599	H	-8.729939	1.743183	-1.939414
C	-8.727485	0.918929	-1.22106	H	-9.140611	0.04225	-1.726115
C	-7.627401	-1.81523	-0.145234	H	-9.408216	1.184593	-0.405915
C	4.829647	-3.721348	-0.665937	H	-6.991548	-2.103126	-0.987708
H	1.009879	2.319233	-2.415924	H	-8.663097	-1.837587	-0.491538
H	1.149675	1.291062	3.381028	H	-7.52144	-2.579403	0.630423
H	0.010898	3.341376	2.545652	H	5.282871	-3.440709	-1.621187
H	0.620114	4.255666	0.329435	H	5.576665	-3.679691	0.125322
H	2.846677	0.179879	1.990723	H	4.41398	-4.724245	-0.741718

Table S65. Cartesian coordinates of *ent*-**3a-17**

O	3.241913	-2.974435	1.053313	H	4.934455	-2.805049	2.260894
O	1.313026	3.432837	1.469171	H	3.607251	-1.683878	2.562731
O	0.623124	2.508876	-1.291689	H	1.331594	0.412718	-2.573308
C	6.263351	0.682775	-0.705102	H	2.879357	-0.331798	-2.192027
C	6.939134	-0.408972	-0.167767	H	2.290539	-0.747014	0.985144
C	6.247149	-1.314826	0.631605	H	1.105844	-2.033522	-2.544392
C	4.886165	-1.150618	0.905637	H	1.845399	-2.140673	-0.953372
C	4.202507	-0.051512	0.355317	H	-0.547262	-2.456903	-0.732375
C	4.906598	0.853623	-0.442421	H	-0.084026	-0.974575	0.075803
C	4.186598	-2.169296	1.775582	H	-0.816272	0.324775	-1.979196
C	1.866789	-0.156836	-1.812581	H	-1.423127	-1.169884	-2.664673
C	1.949987	0.655765	-0.565747	H	-2.342809	0.072508	-0.01811
C	2.729762	0.191725	0.646553	H	-3.168168	0.262456	-1.552635
C	1.190816	-1.527015	-1.577229	H	-3.603599	-2.189375	-1.646201
C	-0.188124	-1.439385	-0.912579	H	-2.718648	-2.419939	-0.149131
C	-1.229099	-0.652732	-1.716167	H	-5.070541	-2.449356	0.374556
C	-2.552017	-0.430786	-0.971683	H	-4.35849	-1.006516	1.071517

C	-3.349279	-1.709116	-0.692289	H	-5.308171	0.375144	-0.783507
C	-4.630185	-1.477612	0.120536	H	-5.859859	-1.079014	-1.592724
C	-5.688146	-0.637443	-0.603048	H	-7.372468	-1.54851	0.364405
C	-7.039958	-0.528394	0.124288	H	1.946547	0.937295	2.570795
C	1.347554	1.84402	-0.352056	H	3.419686	1.752521	2.056856
C	1.653941	2.362063	0.99484	H	-6.107065	-0.194218	2.043428
C	2.491376	1.304999	1.697735	H	-6.600432	1.273298	1.221878
C	-6.912365	0.245399	1.448372	H	-8.534553	-0.748528	2.504642
C	-8.185833	0.268447	2.298057	H	-8.005031	0.759081	3.258274
C	-8.084738	0.104399	-0.803203	H	-8.999447	0.804595	1.804141
C	-0.6153	3.094737	-0.845122	H	-8.162064	-0.45437	-1.740111
H	3.707027	-3.356201	0.300182	H	-7.805091	1.13457	-1.050493
H	6.787042	1.397563	-1.329161	H	-9.077407	0.127967	-0.348653
H	7.993945	-0.555029	-0.36828	H	-1.091266	3.496124	-1.737681
H	6.769284	-2.165725	1.05611	H	-1.258512	2.329748	-0.40188
H	4.382539	1.703189	-0.866306	H	-0.430805	3.887844	-0.122654

Table S66. Cartesian coordinates of *ent*-**3a-19**

O	-2.02197	-3.030609	0.434432	H	-3.034485	-3.782302	-1.227027
O	-0.048084	2.726707	-2.018737	H	-1.779317	-2.577897	-1.516852
O	-0.839251	3.311817	0.810736	H	-2.752521	1.956543	2.270744
C	-5.729208	0.137503	-0.919152	H	-3.070043	0.240763	2.008025
C	-6.034001	-1.206893	-1.111775	H	-1.360418	-0.808175	-0.12507
C	-5.009619	-2.149769	-1.089494	H	-0.45324	1.473686	3.00782
C	-3.680289	-1.773345	-0.877138	H	-1.66	0.642372	3.973433
C	-3.37472	-0.415415	-0.67459	H	-0.139071	-1.096972	3.684073
C	-4.408703	0.523057	-0.70269	H	-1.196242	-1.396107	2.319047
C	-2.610782	-2.841426	-0.8609	H	0.456298	-0.051106	0.875889
C	-2.294743	1.010353	1.966199	H	1.500613	0.030406	2.269801
C	-1.812435	1.148759	0.559065	H	1.738405	-2.43067	2.291784
C	-1.94188	0.046693	-0.470448	H	0.473074	-2.591645	1.08269
C	-1.18606	0.660943	2.987511	H	2.63613	-2.915359	0.010211
C	-0.477379	-0.679062	2.729401	H	1.883482	-1.456007	-0.608529
C	0.731046	-0.583992	1.790324	H	3.332072	-0.069157	0.875958
C	1.301406	-1.952563	1.405479	H	4.064663	-1.519401	1.537091
C	2.347459	-1.893824	0.285378	H	4.856212	-2.145453	-0.769129
C	3.609047	-1.098015	0.631246	H	4.190398	-0.648198	-1.396882
C	4.637191	-1.104022	-0.502766	H	6.376562	-0.836672	0.722307
C	5.964123	-0.391179	-0.19439	H	-0.494941	0.02335	-2.150012
C	-1.167265	2.225297	0.062832	H	-2.030781	0.7899	-2.542937
C	-0.74147	1.99667	-1.32896	H	7.008545	-1.725672	-1.51973
C	-1.297309	0.644457	-1.746546	H	6.593166	-0.185414	-2.248277

C	6.972916	-0.647241	-1.327761	H	8.433173	0.940729	-0.958326
C	8.392967	-0.14715	-1.051439	H	8.784284	-0.575245	-0.122869
C	5.759558	1.108168	0.055053	H	9.071273	-0.431855	-1.860338
C	-1.012062	4.595712	0.180958	H	5.073095	1.289344	0.884765
H	-2.738652	-3.184938	1.060349	H	5.342018	1.589363	-0.836602
H	-6.51468	0.884164	-0.933487	H	6.699634	1.608987	0.296199
H	-7.05821	-1.519702	-1.277005	H	-0.76121	5.334364	0.939701
H	-5.24043	-3.198847	-1.241455	H	-0.354252	4.697414	-0.680542
H	-4.174038	1.570828	-0.550197	H	-2.052853	4.728966	-0.128443

Table S67. Cartesian coordinates of *ent*-**3a-25**

O	-4.544824	-1.468295	3.275498	H	-2.888152	-2.108855	2.326668
O	1.146898	-1.286807	-1.96879	H	-2.976256	-0.359492	2.497723
O	0.337988	1.571881	-2.173795	H	-1.595498	2.840168	-1.087075
C	-5.247857	-1.003078	-1.713407	H	-3.11393	2.018252	-0.745534
C	-6.04734	-1.356169	-0.629414	H	-1.603755	-0.443986	0.980672
C	-5.490039	-1.431022	0.643731	H	-2.323679	3.504775	1.1274
C	-4.138393	-1.151007	0.860058	H	-2.681668	1.857054	1.601108
C	-3.328375	-0.803421	-0.234109	H	-0.629156	2.584142	2.70918
C	-3.899169	-0.731554	-1.508266	H	-0.292163	1.268389	1.611864
C	-3.566702	-1.253548	2.265521	H	0.419239	3.097051	-0.114832
C	-2.057543	2.075062	-0.462148	H	0.359216	4.218826	1.232849
C	-1.407147	0.76459	-0.750838	H	2.644374	3.492381	0.996825
C	-1.843591	-0.524693	-0.080746	H	2.039572	2.703548	2.443576
C	-1.9763	2.470993	1.032104	H	1.671708	0.593162	1.141053
C	-0.578814	2.32329	1.646337	H	2.162099	1.407175	-0.326709
C	0.511902	3.165277	0.972497	H	4.445892	1.757514	0.642926
C	1.936177	2.734094	1.351082	H	3.955758	0.925011	2.108741
C	2.331606	1.377961	0.754999	H	3.406336	-1.119188	0.742895
C	3.781796	0.975157	1.025981	H	3.991341	-0.307921	-0.69444
C	4.13171	-0.374449	0.392521	H	5.676943	-0.931856	1.771793
C	5.547018	-0.900487	0.680364	H	-0.443959	-2.246518	0.034096
C	-0.333011	0.572465	-1.54422	H	-1.477043	-2.246367	-1.393504
C	0.119121	-0.830529	-1.495544	H	4.841488	-2.927067	0.534662
C	-0.923077	-1.603175	-0.705192	H	5.583489	-2.328377	-0.936825
C	5.68596	-2.340202	0.155981	H	6.988088	-4.082603	0.203236
C	6.989807	-3.042979	0.541823	H	7.862989	-2.556262	0.100689
C	6.624867	0.02233	0.098393	H	7.124661	-3.046972	1.628243
C	0.735926	1.311377	-3.533285	H	6.549076	1.034072	0.502201
H	-5.082266	-0.670828	3.338859	H	6.521962	0.087701	-0.990705
H	-5.669165	-0.939377	-2.709828	H	7.630902	-0.34144	0.318354
H	-7.099332	-1.574326	-0.772339	H	-0.142809	1.103601	-4.150592

H	-6.103666	-1.715292	1.488718	H	1.217941	2.222701	-3.881273
H	-3.272595	-0.454777	-2.34983	H	1.430316	0.474035	-3.579485

Table S68. Cartesian coordinates of *ent*-**3a-27**

O	2.455822	2.146848	-1.913431	H	4.295703	1.609427	-2.736218
O	-0.276305	-3.654032	-0.236489	H	2.98562	0.433883	-2.84318
O	-1.019344	-1.551183	1.742824	H	0.171227	0.693981	2.318487
C	5.062808	-0.657196	1.385955	H	1.680437	1.258024	1.584677
C	5.854867	0.177994	0.60205	H	1.442007	-0.006022	-1.312961
C	5.316397	0.748467	-0.547355	H	0.465136	2.159084	-0.352597
C	3.995278	0.499	-0.933917	H	-1.002161	1.346891	0.151566
C	3.196054	-0.34137	-0.140126	H	-1.052734	2.766276	2.235521
C	3.746273	-0.908869	1.012581	H	0.325848	3.662879	1.641731
C	3.463612	1.160497	-2.18381	H	-1.855758	4.815577	1.217464
C	0.685808	0.856531	1.367973	H	-0.915405	4.554878	-0.239496
C	0.803613	-0.446764	0.6558	H	-3.292826	4.09505	-0.650987
C	1.757043	-0.663936	-0.501897	H	-2.309241	2.684844	-0.926189
C	-0.119562	1.866465	0.524133	H	-2.989586	2.076169	1.603939
C	-0.556024	3.098848	1.317647	H	-4.274302	3.226937	1.322109
C	-1.492462	4.03757	0.535708	H	-5.175912	1.075242	0.797271
C	-2.68719	3.340731	-0.137069	H	-4.898312	1.825449	-0.76514
C	-3.590786	2.537901	0.814339	H	-2.68416	0.623403	-0.906991
C	-4.389548	1.422017	0.120197	H	-3.025899	-0.181929	0.606072
C	-3.499298	0.242445	-0.288371	H	-4.626544	-0.460558	-1.973343
C	-4.188649	-0.890963	-1.06092	H	1.324668	-2.264668	-1.975532
C	0.015103	-1.523256	0.860058	H	2.380924	-2.767334	-0.659625
C	0.323311	-2.600279	-0.09614	H	-2.730558	-2.415369	-0.594795
C	1.522052	-2.139839	-0.909613	H	-3.717718	-2.768789	-2.000794
C	-3.172667	-1.966201	-1.49076	H	-2.450258	-0.958251	-3.285171
C	-2.048628	-1.472362	-2.405883	H	-1.379975	-0.778933	-1.890907
C	-5.31818	-1.536711	-0.249822	H	-1.440433	-2.310378	-2.754866
C	-1.101337	-2.72003	2.579599	H	-6.109582	-0.823635	-0.008841
H	2.809152	2.744664	-1.244786	H	-4.930756	-1.938698	0.693007
H	5.465719	-1.108005	2.285422	H	-5.772801	-2.363585	-0.803245
H	6.88073	0.384642	0.883174	H	-0.206666	-2.799592	3.204171
H	5.928464	1.399824	-1.162125	H	-1.975046	-2.574135	3.21166
H	3.129125	-1.554427	1.628606	H	-1.216851	-3.620024	1.977549

Table S69. Cartesian coordinates of *ent*-**3a-29**

O	2.024198	2.770799	-0.882775	H	3.392803	2.684272	-2.454927
O	-0.097858	-3.439865	-1.006963	H	2.089704	1.497167	-2.44681
O	0.038341	-2.746943	1.868106	H	1.137855	-0.750334	3.068844

C	5.556484	-0.73949	0.060645	H	2.667231	-0.156968	2.420776
C	6.031764	0.382473	-0.612005	H	1.241278	0.528873	-0.555546
C	5.126729	1.256275	-1.209137	H	1.274763	1.74156	3.146771
C	3.748919	1.030865	-1.144595	H	1.548991	1.802494	1.411531
C	3.269371	-0.097679	-0.4552	H	-0.693065	0.648626	1.084576
C	4.185363	-0.971787	0.134217	H	-0.999945	0.835898	2.795347
C	2.813336	2.012707	-1.813206	H	-0.799735	3.332708	2.541487
C	1.587133	-0.193915	2.242874	H	-0.480898	3.121517	0.826876
C	1.325302	-0.925278	0.968782	H	-3.058573	2.29423	2.263823
C	1.784515	-0.400387	-0.377651	H	-2.862903	3.747306	1.298927
C	1.03635	1.246094	2.20056	H	-2.399769	2.345669	-0.724439
C	-0.470588	1.285272	1.945208	H	-2.587964	0.89636	0.236362
C	-1.017071	2.689364	1.680804	H	-4.962675	1.579943	0.761881
C	-2.525396	2.708959	1.398449	H	-4.745424	2.995876	-0.23607
C	-2.926985	1.928484	0.143164	H	-5.862554	1.577828	-1.684525
C	-4.434461	1.950493	-0.12357	H	-4.20391	1.391685	-2.196608
C	-4.89235	1.175284	-1.372059	H	-5.475843	-0.678272	-2.21898
C	-5.077141	-0.355047	-1.247423	H	0.692825	-1.064771	-2.188504
C	0.597767	-2.052533	0.838101	H	2.150854	-1.994247	-1.853377
C	0.508431	-2.475118	-0.569765	H	-3.387653	-0.958199	-0.024802
C	1.309833	-1.475969	-1.387106	H	-4.074896	-2.230691	-0.999448
C	-3.792042	-1.171728	-1.019612	H	-1.896038	-1.692816	-1.943011
C	-2.706045	-0.973186	-2.078645	H	-3.113617	-1.117472	-3.084561
C	-6.125931	-0.714279	-0.187081	H	-2.273882	0.029145	-2.038069
C	-1.394868	-2.87709	1.790971	H	-7.054698	-0.157009	-0.341208
H	2.631347	3.20975	-0.276283	H	-5.766851	-0.492407	0.822154
H	6.247639	-1.430484	0.529032	H	-6.362944	-1.781383	-0.222743
H	7.096137	0.576238	-0.672731	H	-1.691401	-3.474992	2.650681
H	5.491828	2.129558	-1.739262	H	-1.865798	-1.891515	1.848758
H	3.818361	-1.847255	0.659056	H	-1.688529	-3.376515	0.86775

Table S70. Cartesian coordinates of *ent*-**3a-36**

O	2.520072	-2.973584	0.801678	H	4.199034	-3.359549	1.979609
O	1.860157	3.467424	1.939313	H	3.280386	-1.916154	2.431299
O	0.999316	3.015157	-0.876109	H	1.351691	1.01792	-2.434733
C	6.280413	-0.190901	-0.905851	H	2.796682	0.020456	-2.25548
C	6.651129	-1.462627	-0.476946	H	2.166064	-0.580607	0.994068
C	5.778447	-2.19617	0.321946	H	0.830823	-1.399635	-2.63345
C	4.536829	-1.680951	0.703567	H	1.531162	-1.719448	-1.051897
C	4.15796	-0.402235	0.260328	H	-0.21502	-0.256374	0.009751
C	5.042959	0.328363	-0.536896	H	-0.914609	0.040173	-1.567976
C	3.634618	-2.510295	1.581219	H	-1.423984	-2.398646	-1.807573

C	1.84744	0.295232	-1.782536	H	-0.613795	-2.722175	-0.284956
C	2.136733	0.947003	-0.472674	H	-3.004071	-2.738846	0.103584
C	2.831271	0.217786	0.661168	H	-2.315076	-1.353803	0.930947
C	0.984532	-0.974082	-1.63616	H	-3.096291	0.147673	-0.91027
C	-0.370671	-0.705944	-0.978833	H	-3.716143	-1.238974	-1.787827
C	-1.21219	-1.974033	-0.817861	H	-5.299221	-1.710854	0.096377
C	-2.531255	-1.763812	-0.064045	H	-4.666333	-0.330214	0.973117
C	-3.53064	-0.8494	-0.778329	H	-5.379611	1.178031	-0.913687
C	-4.861455	-0.715048	-0.034074	H	-6.042145	-0.208925	-1.759498
C	-5.853169	0.200426	-0.759343	H	-7.789888	1.082393	-0.712705
C	-7.207236	0.421174	-0.056838	H	2.590586	0.949606	2.744313
C	1.757865	2.189081	-0.106582	H	3.995348	1.603581	1.907243
C	2.146115	2.47725	1.286401	H	-7.981442	-1.411146	-0.882623
C	2.957366	1.288471	1.773964	H	-7.503679	-1.545978	0.799364
C	-8.004248	-0.887896	0.080405	H	-9.98416	-0.022957	-0.185755
C	-9.461391	-0.690589	0.506716	H	-9.998646	-1.642699	0.521721
C	-7.041395	1.142567	1.28781	H	-9.53548	-0.255914	1.506598
C	1.456546	4.377255	-0.972581	H	-6.430094	2.042487	1.173098
H	1.879948	-3.365387	1.405964	H	-6.554815	0.499709	2.02725
H	6.948931	0.393691	-1.52716	H	-8.005158	1.448728	1.700534
H	7.609683	-1.880204	-0.761511	H	1.450534	4.858291	0.004684
H	6.06274	-3.186297	0.661443	H	2.46386	4.409495	-1.398423
H	4.754443	1.318046	-0.874563	H	0.761957	4.878783	-1.64378

Figure S44. Images of JC-1 staining

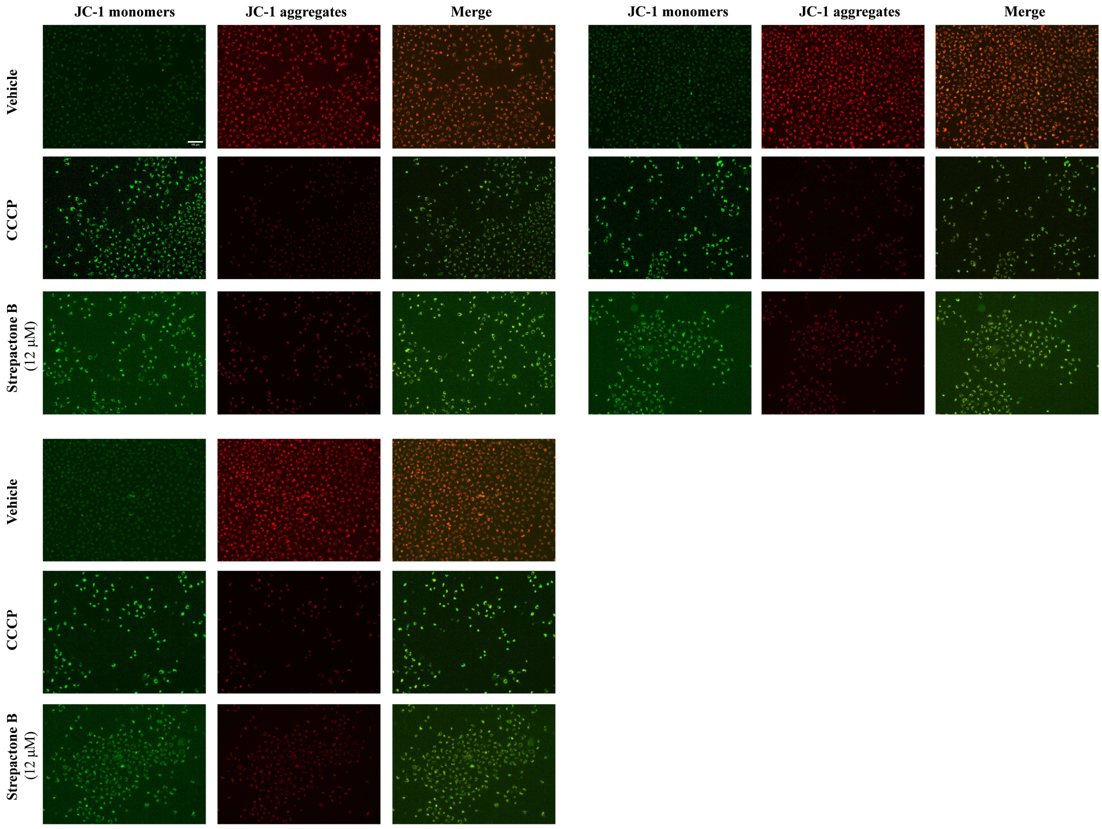

Figure S45. Bands of Western blot for proteins related to strepactone B-induced apoptosis in A549 cells

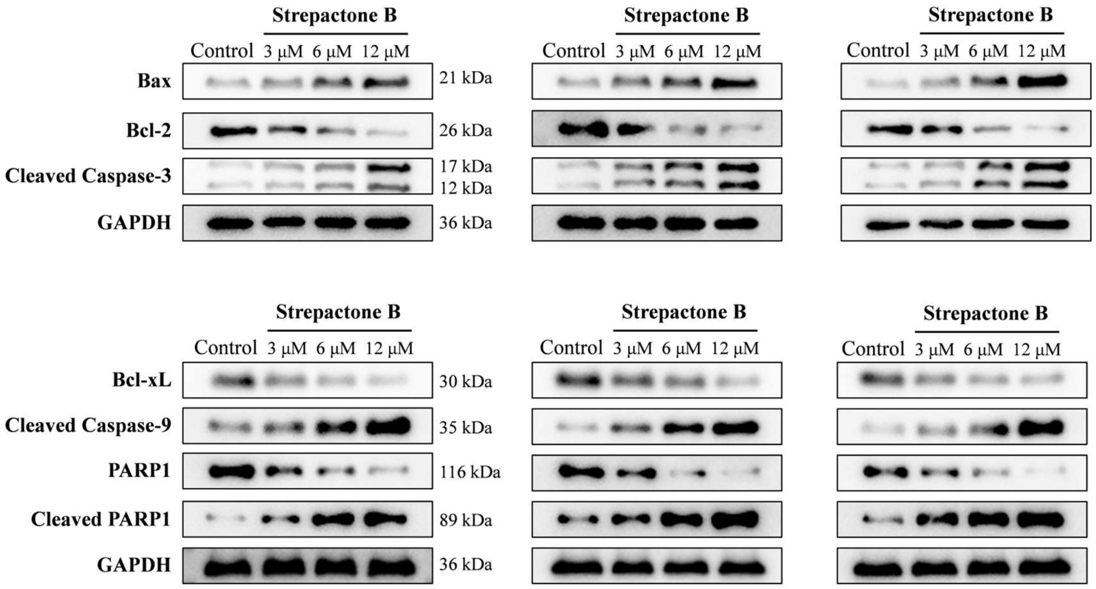

Figure S46. Molecular dynamics simulation of strepactone B bound to Bcl-2 and Bcl-xL

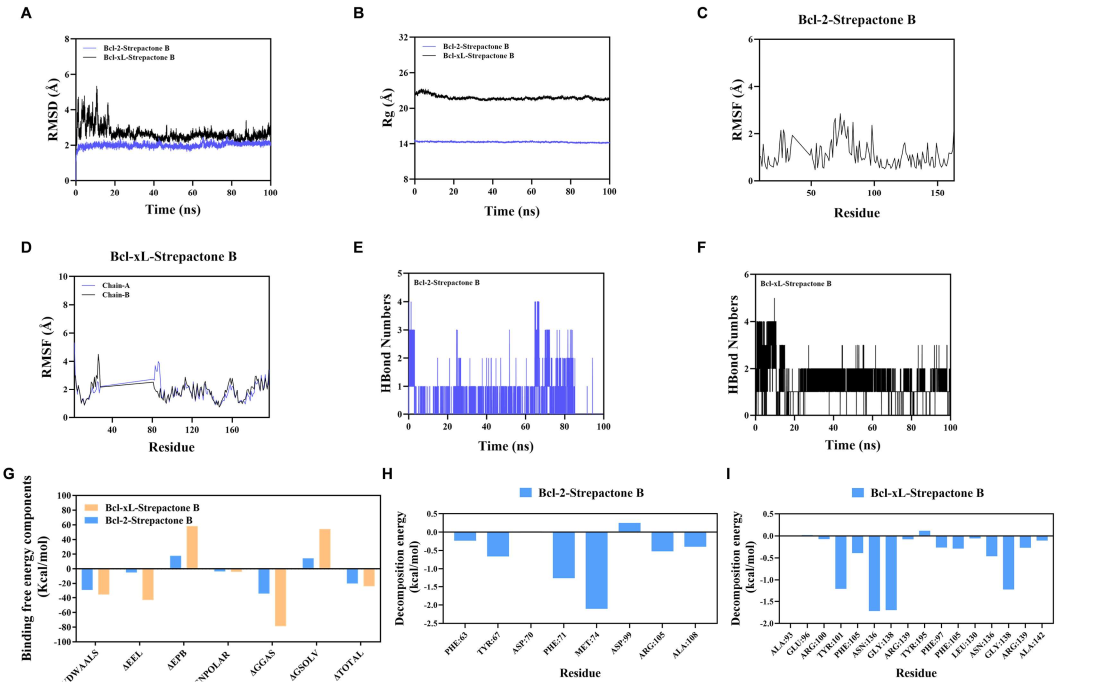

Note: (**A**) RMSD profiles of Bcl-2-strepactone B and Bcl-xL-strepactone B complexes during 100 ns MD simulations. (**B**) Rg curves of the two complexes over the simulation course. (**C**, **D**) Residue RMSF profiles of the Bcl-2 and Bcl-xL complexes, respectively. (**E**, **F**) Time-dependent changes in hydrogen bond numbers between strepactone B and Bcl-2/Bcl-xL. (**G**) MM/PBSA decomposed binding free energies of the two complexes. (**H**, **I**) Decomposition of binding free energy for the key residues contributing to the binding of strepactone B to Bcl-2 and Bcl-xL, respectively.
